# Large-scale phenotyping of patients with long COVID post-hospitalization reveals mechanistic subtypes of disease

**DOI:** 10.1038/s41590-024-01778-0

**Published:** 2024-04-08

**Authors:** Felicity Liew, Claudia Efstathiou, Sara Fontanella, Matthew Richardson, Ruth Saunders, Dawid Swieboda, Jasmin K. Sidhu, Stephanie Ascough, Shona C. Moore, Noura Mohamed, Jose Nunag, Clara King, Olivia C. Leavy, Omer Elneima, Hamish J. C. McAuley, Aarti Shikotra, Amisha Singapuri, Marco Sereno, Victoria C. Harris, Linzy Houchen-Wolloff, Neil J. Greening, Nazir I. Lone, Matthew Thorpe, A. A. Roger Thompson, Sarah L. Rowland-Jones, Annemarie B. Docherty, James D. Chalmers, Ling-Pei Ho, Alexander Horsley, Betty Raman, Krisnah Poinasamy, Michael Marks, Onn Min Kon, Luke S. Howard, Daniel G. Wootton, Jennifer K. Quint, Thushan I. de Silva, Antonia Ho, Christopher Chiu, Ewen M. Harrison, William Greenhalf, J. Kenneth Baillie, Malcolm G. Semple, Lance Turtle, Rachael A. Evans, Louise V. Wain, Christopher Brightling, Ryan S. Thwaites, Peter J. M. Openshaw, PHOSP-COVID collaborative group, Kathryn Abel, H. Adamali, Davies Adeloye, Oluwaseun Adeyemi, Rita Adrego, Laura Aguilar Jimenez, Shanaz Ahmad, N. Ahmad Haider, Rubina Ahmed, Nyarko Ahwireng, Mark Ainsworth, Asma Alamoudi, Mariam Ali, M. Aljaroof, Louise Allan, Richard Allen, Lisa Allerton, Lynne Allsop, Ann Marie Allt, Paula Almeida, Bashar Al-Sheklly, Danny Altmann, Maria Alvarez Corral, Shannon Amoils, David Anderson, Charalambos Antoniades, Gill Arbane, Ava Maria Arias, Cherie Armour, Lisa Armstrong, Natalie Armstrong, David Arnold, H. Arnold, A. Ashish, Andrew Ashworth, M. Ashworth, Shahab Aslani, Hosanna Assefa-Kebede, Paul Atkin, Catherine Atkin, Raminder Aul, Hnin Aung, Liam Austin, Cristina Avram, Nikos Avramidis, A. Ayoub, Marta Babores, Rhiannon Baggott, J. Bagshaw, David Baguley, Elisabeth Bailey, J. Kenneth Baillie, Steve Bain, Majda Bakali, M. Bakau, E. Baldry, Molly Baldwin, David Baldwin, Clive Ballard, Amita Banerjee, Dongchun Bang, R. E. Barker, Laura Barman, Perdita Barran, Shaney Barratt, Fiona Barrett, Donna Basire, Neil Basu, Michelle Bates, A. Bates, R. Batterham, Helen Baxendale, Gabrielle Baxter, Hannah Bayes, M. Beadsworth, Paul Beckett, M. Beggs, M. Begum, Paul Beirne, Murdina Bell, Robert Bell, Kaytie Bennett, Eva Beranova, Areti Bermperi, Anthony Berridge, Colin Berry, Sarah Betts, Emily Bevan, Kamaldeep Bhui, Michelle Bingham, K. Birchall, Lettie Bishop, Karen Bisnauthsing, John Blaikely, Angela Bloss, Annette Bolger, Charlotte Bolton, J. Bonnington, A. Botkai, Charlotte Bourne, Michelle Bourne, Kate Bramham, Lucy Brear, G. Breen, Jonathon Breeze, Katie Breeze, Andrew Briggs, E. Bright, Simon Brill, K. Brindle, Lauren Broad, Andrew Broadley, Claire Brookes, Mattew Broome, Vanessa Brown, M. Brown, Jo Brown, J. Brown, Ammani Brown, Angela Brown, M. Brown, Jeremy Brown, Terry Brugha, Nigel Brunskill, M. Buch, Phil Buckley, Anda Bularga, Ed Bullmore, Jenny Bunker, L. Burden, Tracy Burdett, David Burn, G. Burns, A. Burns, John Busby, Robyn Butcher, Al-Tahoor Butt, S. Byrne, P. Cairns, P. C. Calder, Ellen Calvelo, H. Carborn, Bethany Card, Caitlin Carr, Liesel Carr, G. Carson, Penny Carter, Anna Casey, M. Cassar, Jonathon Cavanagh, Manish Chablani, Trudie Chalder, James D. Chalmers, Rachel Chambers, Flora Chan, K. M. Channon, Kerry Chapman, Amanda Charalambou, N. Chaudhuri, A. Checkley, Jin Chen, Yutung Cheng, Luke Chetham, Caroline Childs, Edwin Chilvers, H. Chinoy, A. Chiribiri, K. Chong-James, N. Choudhury, Gaunab Choudhury, Phillip Chowienczyk, C. Christie, Melanie Chrystal, Cameron Clark, David Clark, Jude Clarke, S. Clohisey, G. Coakley, Zach Coburn, S. Coetzee, Joby Cole, Chris Coleman, Florence Conneh, David Connell, Bronwen Connolly, Lynda Connor, Amanda Cook, Shirley Cooper, B. Cooper, Josh Cooper, Donna Copeland, Tracey Cosier, Eamon Coughlan, Martina Coulding, C. Coupland, E. Cox, Thelma Craig, P. Crisp, Daniele Cristiano, Michael Crooks, Andy Cross, Isabel Cruz, P. Cullinan, D. Cuthbertson, Luke Daines, Matthhew Dalton, Patrick Daly, Alison Daniels, P. Dark, Joanne Dasgin, C. David, Anthony David, Ffyon Davies, Ellie Davies, Kim Davies, Gareth Davies, Gwyneth Davies, Melanie Davies, Joy Dawson, Camilla Dawson, Enya Daynes, Anthony De Soyza, Bill Deakin, Andrew Deans, C. Deas, Joanne Deery, Sylviane Defres, Amanda Dell, K. Dempsey, Emma Denneny, J. Dennis, A. Dewar, Ruvini Dharmagunawardena, Nawar Diar-Bakerly, Caroline Dickens, A. Dipper, Sarah Diver, Shalin Diwanji, Myles Dixon, R. Djukanovic, Hannah Dobson, S. L. Dobson, Annemarie B. Docherty, A. Donaldson, T. Dong, N. Dormand, Andrew Dougherty, Rachael Dowling, Stephen Drain, Katharine Draxlbauer, Katie Drury, Pearl Dulawan, A. Dunleavy, Sarah Dunn, Catherine Dupont, Joanne Earley, Nicholas Easom, Carlos Echevarria, Sarah Edwards, C. Edwardson, Anne Elliott, K. Elliott, Yvette Ellis, Anne Elmer, Hosni El-Taweel, Teriann Evans, Ranuromanana Evans, D. Evans, R. Evans, H. Evans, Jonathon Evans, Cerys Evenden, Lynsey Evison, Laura Fabbri, Sara Fairbairn, Alexandra Fairman, K. Fallon, David Faluyi, Clair Favager, Tamanah Fayzan, James Featherstone, T. Felton, V. Ferreira, J. Finch, Selina Finney, J. Finnigan, L. Finnigan, Helen Fisher, S. Fletcher, Rachel Flockton, Margaret Flynn, H. Foot, David Foote, Amber Ford, D. Forton, Eva Fraile, C. Francis, Richard Francis, Susan Francis, Anew Frankel, Emily Fraser, Rob Free, N. French, X. Fu, Jonathon Fuld, J. Furniss, Lucie Garner, N. Gautam, John Geddes, J. George, P. George, Michael Gibbons, Rhyan Gill, Mandy Gill, L. Gilmour, F. Gleeson, Jodie Glossop, Sarah Glover, Nicola Goodman, Camelia Goodwin, Bibek Gooptu, Hussain Gordon, T. Gorsuch, M. Greatorex, Paul Greenhaff, William Greenhalf, Alan Greenhalgh, John Greenwood, Rebecca Gregory, Heidi Gregory, D. Grieve, Denise Griffin, L. Griffiths, Anne-Marie Guerdette, Beatriz Guillen-Guio, Mahitha Gummadi, Ayushman Gupta, Sambasivarao Gurram, Elspeth Guthrie, Zoe Guy, Kate Hadley, Ahmed Haggar, Kera Hainey, Brigid Hairsine, Pranab Haldar, Lucy Hall, Ian Hall, Mark Halling-Brown, R. Hamil, Alyson Hancock, Kia Hancock, Neil Hanley, Sulaimaan Haq, Hayley Hardwick, Tim Hardy, E. Hardy, Beverley Hargadon, Kate Harrington, Edward Harris, Ewen Harrison, Paul Harrison, Nicholas Hart, Alice Harvey, Matt Harvey, M. Harvie, L. Haslam, Claire Hastie, May Havinden-Williams, Jenny Hawkes, Nancy Hawkings, Jill Haworth, A. Hayday, Matthew Haynes, J. Hazeldine, Tracy Hazelton, Liam Heaney, Cheryl Heeley, Jonathon Heeney, M. Heightman, Simon Heller, Max Henderson, Helen Henson, L. Hesselden, Melanie Hewitt, Victoria Highett, T. Hillman, T. Hiwot, Ling-Pei Ho, Michaela Hoare, Amy Hoare, J. Hockridge, Philip Hogarth, Ailsa Holbourn, Sophie Holden, L. Holdsworth, D. Holgate, Maureen Holland, Leah Holloway, Katie Holmes, Megan Holmes, B. Holroyd-Hind, L. Holt, Anil Hormis, Alexander Horsley, Akram Hosseini, M. Hotopf, Luke S. Howard, Kate Howard, Alice Howell, E. Hufton, Rachel Ann Hughes, Joan Hughes, Alun Hughes, Amy Humphries, Nathan Huneke, E. Hurditch, John Hurst, Masud Husain, Tracy Hussell, John Hutchinson, W. Ibrahim, F. Ilyas, Julie Ingham, L. Ingram, Diana Ionita, Karen Isaacs, Khalida Ismail, T. Jackson, Joseph Jacob, W. Y. James, W. Jang, Claire Jarman, Ian Jarrold, Hannah Jarvis, Roman Jastrub, Bhagy Jayaraman, Gisli Jenkins, P. Jezzard, Kasim Jiwa, C. Johnson, Simon Johnson, Desmond Johnston, Caroline Jolley, S. Jones, H. Jones, L. Jones, Ian Jones, G. Jones, Heather Jones, Mark Jones, Don Jones, Sherly Jose, Thomas Kabir, G. Kaltsakas, Vicky Kamwa, N. Kanellakis, Sabina Kaprowska, Zunaira Kausar, Natalie Keenan, S. Kelly, G. Kemp, Steven Kerr, Helen Kerslake, Angela Key, Fasih Khan, Kamlesh Khunti, Susan Kilroy, Bernie King, Clara King, Lucy Kingham, Jill Kirk, Paaig Kitterick, Paul Klenerman, Lucy Knibbs, Sean Knight, Abigail Knighton, Onn Min Kon, S. Kon, Samantha Kon, Ania Korszun, Ivan Koychev, Claire Kurasz, Prathiba Kurupati, C. Laing, Hanan Lamlum, G. Landers, Claudia Langenberg, Lara Lavelle-Langham, Allan Lawrie, Cathy Lawson, Claire Lawson, Alison Layton, A. Lea, Ju Hee Lee, Elvina Lee, D. Lee, Karen Leitch, Rebecca Lenagh, Victoria Lewis, Joanne Lewis, Keir Lewis, D. Lewis, N. Lewis-Burke, X. Li, Tessa Light, Liz Lightstone, W. Lilaonitkul, Lai Lim, S. Linford, Anne Lingford-Hughes, M. Lipman, Kamal Liyanage, Arwel Lloyd, S. Logan, D. Lomas, Nazir I. Lone, Ronda Loosley, Janet Lord, Harpreet Lota, Wayne Lovegrove, Daniel Lozano-Rojas, Alice Lucey, Gardiner Lucy, E. Lukaschuk, Alison Lye, Ceri Lynch, S. MacDonald, G. MacGowan, Irene Macharia, J. Mackie, L. Macliver, S. Madathil, Gladys Madzamba, Nick Magee, Murphy Magtoto, N. Mairs, N. Majeed, E. Major, Flora Malein, M. Malim, Georgia Mallison, William Man, S. Mandal, K. Mangion, C. Manisty, R. Manley, Katherine March, Stefan Marciniak, Philip Marino, Myril Mariveles, Michael Marks, Elizabeth Marouzet, Sophie Marsh, M. Marshall, B. Marshall, Jane Martin, Adrian Martineau, L. M. Martinez, Nick Maskell, Darwin Matila, Wadzanai Matimba-Mupaya, Laura Matthews, Angeline Mbuyisa, Steve McAdoo, Hamish McAllister-Williams, Paul McArdle, Anne McArdle, Danny McAulay, Gerry McCann, W. McCormick, Jacqueline McCormick, P. McCourt, Celeste McCracken, Lorcan McGarvey, C. McGee, K. Mcgee, Jade McGinness, K. McGlynn, Andrew McGovern, Heather McGuinness, I. B. McInnes, Jerome McIntosh, Emma McIvor, Katherine McIvor, Laura McLeavey, Aisling McMahon, Michael McMahon, L. McMorrow, Teresa Mcnally, M. McNarry, J. McNeill, Alison McQueen, H. McShane, Chloe Mears, Clare Megson, Sharon Megson, P. Mehta, J. Meiring, Lucy Melling, Mark Mencias, R. Menke, Daniel Menzies, Marta Merida Morillas, Alice Michael, Benedict Michael, C. A. Miller, Lea Milligan, Nicholas Mills, Clare Mills, George Mills, L. Milner, S. Misra, Jane Mitchell, Abdelrahman Mohamed, S. Mohammed, Philip Molyneaux, Will Monteiro, Silvia Moriera, Anna Morley, Leigh Morrison, Richard Morriss, A. Morrow, Paul Moss, Alistair Moss, K. Motohashi, N. Msimanga, Elizabeta Mukaetova-Ladinska, Unber Munawar, Jennifer Murira, Uttam Nanda, Heeah Nassa, Mariam Nasseri, Rashmita Nathu, Aoife Neal, Robert Needham, Paula Neill, Stefan Neubauer, D. E. Newby, Helen Newell, J. Newman, Tom Newman, Alex Newton-Cox, T. E. Nichols, Tim Nicholson, Christos Nicolaou, Debby Nicoll, Athanasios Nikolaidis, C. Nikolaidou, C. M. Nolan, Matthew Noonan, C. Norman, Petr Novotny, Kimon Ntotsis, Lorenza Nwafor, Uchechi Nwanguma, Joseph Nyaboko, Linda O’Brien, C. O’Brien, Natasha Odell, Kate O’Donnell, Godwin Ogbole, G. Ogg, Olaoluwa Olaosebikan, Catherine Oliver, Zohra Omar, D. P. O’Regan, Lorna Orriss-Dib, Lynn Osborne, Rebecca Osbourne, Marlies Ostermann, Charlotte Overton, J. Owen, J. Oxton, Jamie Pack, Edmund Pacpaco, Stella-Maria Paddick, Sharon Painter, Erola Pairo-Castineira, Ashkan Pakzad, Sue Palmer, Padmasayee Papineni, K. Paques, Kerry Paradowski, Manish Pareek, Dhruv Parekh, H. Parfrey, Carmen Pariante, S. Parker, M. Parkes, J. Parmar, Sheetal Patale, Manish Patel, B. Patel, Suhani Patel, Dibya Pattenadk, M. Pavlides, Sheila Payne, Lorraine Pearce, John Pearl, Dan Peckham, Jessica Pendlebury, Yanchun Peng, Chris Pennington, Ida Peralta, Emma Perkins, Z. Peterkin, Tunde Peto, Nayia Petousi, John Petrie, Paul Pfeffer, Janet Phipps, S. Piechnik, John Pimm, Karen Piper Hanley, Riinu Pius, Hannah Plant, S. Plein, Tatiana Plekhanova, Megan Plowright, Krisnah Poinasamy, Oliver Polgar, L. Poll, Julie Porter, Joanna Porter, Sofiya Portukhay, Natassia Powell, A. Prabhu, James Pratt, Andrea Price, Claire Price, Carly Price, L. Price, D. Price, L. Price, Anne Prickett, I. Propescu, J. Propescu, Sabrina Prosper, S. Pugmire, Sheena Quaid, Jackie Quigley, Jennifer K. Quint, H. Qureshi, I. N. Qureshi, K. Radhakrishnan, Najib Rahman, Markus Ralser, Betty Raman, Hazel Ramos, Albert Ramos, Jade Rangeley, Bojidar Rangelov, Liz Ratcliffe, Phillip Ravencroft, Konrad Rawlik, Anne Reddington, R. Reddy, A. Reddy, Heidi Redfearn, Dawn Redwood, Annabel Reed, Meryl Rees, Tabitha Rees, Karen Regan, Will Reynolds, Carla Ribeiro, A. Richards, Emma Richardson, M. Richardson, Pilar Rivera-Ortega, K. Roberts, Elizabeth Robertson, Leanne Robinson, Emma Robinson, Lisa Roche, C. Roddis, J. Rodger, Natalie Rogers, Gavin Ross, Alexandra Ross, Jennifer Rossdale, Anthony Rostron, Anna Rowe, J. Rowland, M. J. Rowland, A. Rowland, Sarah L. Rowland-Jones, Maura Roy, K. Roy, Igor Rudan, Richard Russell, Emily Russell, Gwen Saalmink, Ramsey Sabit, Beth Sage, T. Samakomva, Nilesh Samani, A. A. Samat, Claire Sampson, Katherine Samuel, Reena Samuel, Z. B. Sanders, Amy Sanderson, Elizabeth Sapey, Dinesh Saralaya, Jack Sargant, Carol Sarginson, T. Sass, Naveed Sattar, Kathryn Saunders, Peter Saunders, Ruth Saunders, Laura Saunders, Heather Savill, W. Saxon, Avan Sayer, J. Schronce, William Schwaeble, Janet Scott, Kathryn Scott, Nick Selby, Terri Ann Sewell, Kamini Shah, Ajay Shah, P. Shah, Manu Shankar-Hari, M. Sharma, Claire Sharpe, Michael Sharpe, Sharlene Shashaa, Alison Shaw, Victoria Shaw, Karen Shaw, Aziz Sheikh, Sarah Shelton, Liz Shenton, K. Shevket, J. Short, Sulman Siddique, Salman Siddiqui, J. Sidebottom, Louise Sigfrid, Gemma Simons, Neil Simpson, John Simpson, Ananga Singapuri, Suver Singh, Claire Singh, Sally Singh, D. Sissons, J. Skeemer, Katie Slack, David Smith, Nikki Smith, Andrew Smith, Jacqui Smith, Laurie Smith, Susan Smith, M. Soares, Teresa Solano, Reanne Solly, A. R. Solstice, Tracy Soulsby, David Southern, D. Sowter, Mark Spears, Lisa Spencer, Fabio Speranza, Louise Stadon, Stefan Stanel, R. Steeds, N. Steele, Mike Steiner, David Stensel, G. Stephens, Lorraine Stephenson, M. Stern, Iain Stewart, R. Stimpson, Sue Stockdale, J. Stockley, Wendy Stoker, Roisin Stone, Will Storrar, Andrew Storrie, Kim Storton, E. Stringer, Sophia Strong-Sheldrake, Natalie Stroud, Christian Subbe, Catherine Sudlow, Zehra Suleiman, Charlotte Summers, C. Summersgill, Debbie Sutherland, D. L. Sykes, R. Sykes, Nick Talbot, Ai Lyn Tan, Lawrence Tarusan, Vera Tavoukjian, Jessica Taylor, Abigail Taylor, Chris Taylor, John Paul Taylor, Amelie Te, H. Tedd, Caroline Tee, J. Teixeira, Helen Tench, Sarah Terry, Susannah Thackray-Nocera, Favas Thaivalappil, B. Thamu, David Thickett, David Thomas, S. Thomas, Caradog Thomas, Andrew Thomas, T. Thomas-Woods, A. A. Roger Thompson, Tamika Thompson, T. Thornton, Matthew Thorpe, Jo Tilley, N. Tinker, Gerlynn Tiongson, Martin Tobin, Johanne Tomlinson, C. Tong, Mark Toshner, R. Touyz, T. Treibel, K. A. Tripp, Drupad Trivedi, E. M. Tunnicliffe, Alison Turnbull, Kim Turner, Sarah Turner, Victoria Turner, E. Turner, Sharon Turney, Helena Turton, Jacinta Ugoji, R. Ugwuoke, Rachel Upthegrove, Jonathon Valabhji, Maximina Ventura, Joanne Vere, Carinna Vickers, Ben Vinson, Ioannis Vogiatzis, Elaine Wade, Phillip Wade, Tania Wainwright, Lilian Wajero, Sinead Walder, Samantha Walker, S. Walker, E. Wall, Tim Wallis, Sarah Walmsley, Simon Walsh, J. A. Walsh, Louise Warburton, T. J. C. Ward, Katie Warwick, Helen Wassall, Samuel Waterson, L. Watson, Ekaterina Watson, James Watson, M. Webster, J. Weir McCall, H. Welch, Carly Welch, B. Welsh, Simon Wessely, Sophie West, Heather Weston, Helen Wheeler, Sonia White, Victoria Whitehead, J. Whitney, S. Whittaker, Beverley Whittam, V. Whitworth, Andrew Wight, James Wild, Martin Wilkins, Dan Wilkinson, Nick Williams, N. Williams, B. Williams, Jenny Williams, S. A. Williams-Howard, Michelle Willicombe, Gemma Willis, James Willoughby, Ann Wilson, Imogen Wilson, Daisy Wilson, Nicola Window, M. Witham, Rebecca Wolf-Roberts, Chloe Wood, F. Woodhead, Janet Woods, Dan Wootton, J. Wormleighton, J. Worsley, David Wraith, Caroline Wrey Brown, C. Wright, S. Wright, Louise Wright, J. Wyles, Inez Wynter, C. Xie, Moucheng Xu, Najira Yasmin, S. Yasmin, Tom Yates, Kay Por Yip, Susan Young, Bob Young, A. Young, A. J. Yousuf, Amira Zawia, Lisa Zeidan, Bang Zhao, Bang Zheng, O. Zongo, ISARIC investigators, Kayode Adeniji, Daniel Agranoff, Ken Agwuh, Katie A. Ahmed, Dhiraj Ail, Erin L. Aldera, Ana Alegria, Beatrice Alex, Sam Allen, Petros Andrikopoulos, Brian Angus, Jane A. Armstrong, Abdul Ashish, Milton Ashworth, Innocent G. Asiimwe, Dougal Atkinson, Benjamin Bach, J. Kenneth Baillie, Siddharth Bakshi, Wendy S. Barclay, Shahedal Bari, Gavin Barlow, Samantha L. Barlow, Stella Barnass, Nicholas Barrett, Christopher Bassford, Sneha Basude, David Baxter, Michael Beadsworth, Jolanta Bernatoniene, John Berridge, Colin Berry, Nicola Best, Debby Bogaert, Laura Booth, Pieter Bothma, Benjamin Brennan, Robin Brittain-Long, Katie Bullock, Naomi Bulteel, Tom Burden, Andrew Burtenshaw, Nicola Carlucci, Gail Carson, Vikki Caruth, Emily Cass, Benjamin W. A. Catterall, David Chadwick, Duncan Chambler, Meera Chand, Kanta Chechi, Nigel Chee, Jenny Child, Srikanth Chukkambotla, Richard Clark, Tom Clark, Jordan J. Clark, Emily A. Clarke, Sara Clohisey, Sarah Cole, Paul Collini, Marie Connor, Graham S. Cooke, Louise Cooper, Catherine Cosgrove, Audrey Coutts, Helen Cox, Jason Cupitt, Maria-Teresa Cutino-Moguel, Ana da Silva Filipe, Jo Dalton, Paul Dark, Christopher Davis, Chris Dawson, Thushan de Silva, Samir Dervisevic, Oslem Dincarslan, Alejandra Doce Carracedo, Annemarie B. Docherty, Cara Donegan, Lorna Donelly, Phil Donnison, Chloe Donohue, Gonçalo dos Santos Correia, Sam Douthwaite, Thomas M. Drake, Andrew Drummond, Marc-Emmanuel Dumas, Chris Dunn, Jake Dunning, Ingrid DuRand, Ahilanadan Dushianthan, Tristan Dyer, Philip Dyer, Angela Elliott, Cariad Evans, Anthony Evans, Chi Eziefula, Cameron J. Fairfield, Angie Fawkes, Chrisopher Fegan, Lorna Finch, Adam Finn, Lewis W. S. Fisher, Lisa Flaherty, Tom Fletcher, Terry Foster, Duncan Fullerton, Carrol Gamble, Isabel Garcia-Dorival, Atul Garg, Sanjeev Garg, Tammy Gilchrist, Michelle Girvan, Effrossyni Gkrania-Klotsas, Jo Godden, Arthur Goldsmith, Clive Graham, Tassos Grammatikopoulos, Christopher A. Green, William Greenhalf, Julian Griffin, Fiona Griffiths, Philip Gunning, Rishi K. Gupta, Katarzyna Hafezi, Sophie Halpin, Hayley Hardwick, Elaine Hardy, Ewen M. Harrison, Janet Harrison, Catherine Hartley, Stuart Hartshorn, Daniel Harvey, Peter Havalda, Daniel B. Hawcutt, Ross Hendry, Antonia Y. W. Ho, Maria Hobrok, Luke Hodgson, Karl Holden, Anthony Holmes, Peter W. Horby, Anil Hormis, Joanne Howard, Samreen Ijaz, Clare Jackson, Michael Jacobs, Susan Jain, Paul Jennings, Rebecca L. Jensen, Christopher B. Jones, Trevor R. Jones, Agilan Kaliappan, Vidya Kasipandian, Seán Keating, Stephen Kegg, Michael Kelsey, Jason Kendall, Caroline Kerrison, Ian Kerslake, Shadia Khandaker, Say Khoo, Katharine King, Robyn T. Kiy, Paul Klenerman, Stephen R. Knight, Susan Knight, Oliver Koch, Gouri Koduri, George Koshy, Chrysa Koukorava, Shondipon Laha, Eva Lahnsteiner, Steven Laird, Annette Lake, Suzannah Lant, Susan Larkin, Diane Latawiec, Lara Lavelle-Langham, Andrew Law, James Lee, Gary Leeming, Daniella Lefteri, Tamas Leiner, Lauren Lett, Matthew Lewis, Sonia Liggi, Patrick Lillie, Wei Shen Lim, James Limb, Vanessa Linnett, Jeff Little, Lucia A. Livoti, Mark Lyttle, Louise MacGillivray, Alan Maclean, Michael MacMahon, Emily MacNaughton, Maria Mancini, Ravish Mankregod, Laura Marsh, Lynn Maslen, Hannah Massey, Huw Masson, Elijah Matovu, Nicole Maziere, Sarah McCafferty, Katherine McCullough, Sarah E. McDonald, Sarah McDonald, Laurence McEvoy, Ruth McEwen, John McLauchlan, Kenneth A. Mclean, Manjula Meda, Alexander J. Mentzer, Laura Merson, Soeren Metelmann, Alison M. Meynert, Nahida S. Miah, Joanna Middleton, Gary Mills, Jane Minton, Joyce Mitchell, Kavya Mohandas, Quen Mok, James Moon, Elinoor Moore, Shona C. Moore, Patrick Morgan, Kirstie Morrice, Craig Morris, Katherine Mortimore, Samuel Moses, Mbiye Mpenge, Rohinton Mulla, Derek Murphy, Lee Murphy, Michael Murphy, Ellen G. Murphy, Thapas Nagarajan, Megan Nagel, Mark Nelson, Lisa Norman, Lillian Norris, Lucy Norris, Mahdad Noursadeghi, Michael Olanipekun, Wilna Oosthuyzen, Peter J. M. Openshaw, Anthonia Osagie, Matthew K. O’Shea, Marlies Ostermann, Igor Otahal, Mark Pais, Massimo Palmarini, Carlo Palmieri, Selva Panchatsharam, Danai Papakonstantinou, Padmasayee Papineni, Hassan Paraiso, Brij Patel, Natalie Pattison, William A. Paxton, Rebekah Penrice-Randal, Justin Pepperell, Mark Peters, Mandeep Phull, Jack Pilgrim, Stefania Pintus, Riinu Pius, Tim Planche, Daniel Plotkin, Georgios Pollakis, Frank Post, Nicholas Price, David Price, Tessa Prince, Rachel Prout, Nikolas Rae, Andrew Rambaut, Henrik Reschreiter, Tim Reynolds, Neil Richardson, P. Matthew Ridley, Mark Roberts, Stephanie Roberts, Devender Roberts, David L. Robertson, Alistair Rose, Guy Rousseau, Bobby Ruge, Clark D. Russell, Brendan Ryan, Debby Sales, Taranprit Saluja, Vanessa Sancho-Shimizu, Caroline Sands, Egle Saviciute, Matthias Schmid, Janet T. Scott, James Scott-Brown, Malcolm G. Semple, Aarti Shah, Manu Shankar-Hari, Prad Shanmuga, Anil Sharma, Catherine A. Shaw, Victoria E. Shaw, Anna Shawcross, Rebecca K. Shears, Louise Sigfrid, Jagtur Singh Pooni, Jeremy Sizer, Benjamin Small, Richard Smith, Catherine Snelson, Tom Solomon, Rebecca G. Spencer, Nick Spittle, Shiranee Sriskandan, Nikki Staines, Tom Stambach, Richard Stewart, David Stuart, Krishanthi S. Subramaniam, Pradeep Subudhi, Charlotte Summers, Olivia V. Swann, Tamas Szakmany, Agnieska Szemiel, Aislynn Taggart, Sarah Tait, Zoltan Takats, Panteleimon Takis, Jolanta Tanianis-Hughes, Kate Tatham, Richard S. Tedder, Jo Thomas, Jordan Thomas, Robert Thompson, Chris Thompson, Emma C. Thomson, Ascanio Tridente, Erwan Trochu, Darell Tupper-Carey, Lance C. W. Turtle, Mary Twagira, Nick Vallotton, Libby van Tonder, Rama Vancheeswaran, Rachel Vincent, Lisa Vincent-Smith, Shico Visuvanathan, Alan Vuylsteke, Sam Waddy, Rachel Wake, Andrew Walden, Ingeborg Welters, Murray Wham, Tony Whitehouse, Paul Whittaker, Ashley Whittington, Meme Wijesinghe, Eve Wilcock, Martin Williams, Lawrence Wilson, Stephen Winchester, Martin Wiselka, Adam Wolverson, Daniel G. Wootton, Andrew Workman, Nicola Wrobel, Bryan Yates, Peter Young, Maria Zambon, J. Eunice Zhang

**Affiliations:** 1 National Heart and Lung Institute, Imperial College London, London, UK; 2 Institute for Lung Health, Leicester NIHR Biomedical Research Centre, University of Leicester, Leicester, UK; 3 NIHR Health Protection Research Unit in Emerging and Zoonotic Infections, Institute of Infection, Veterinary and Ecological Sciences, University of Liverpool, Liverpool, UK; 4 The Imperial Clinical Respiratory Research Unit, Imperial College NHS Trust, London, UK; 5 Cardiovascular Research Team, Imperial College Healthcare NHS Trust, London, UK; 6 Department of Population Health Sciences, University of Leicester, Leicester, UK; 7 NIHR Leicester Biomedical Research Centre, University of Leicester, Leicester, UK; 8 Centre for Exercise and Rehabilitation Science, NIHR Leicester Biomedical Research Centre-Respiratory, University of Leicester, Leicester, UK; 9 Usher Institute, University of Edinburgh, Edinburgh, UK; 10 Centre for Medical Informatics, The Usher Institute, University of Edinburgh, Edinburgh, UK; 11 Department of Infection, Immunity and Cardiovascular Disease, University of Sheffield, Sheffield, UK; 12 University of Dundee, Ninewells Hospital and Medical School, Dundee, UK; 13 MRC Human Immunology Unit, University of Oxford, Oxford, UK; 14 Division of Infection, Immunity and Respiratory Medicine, Faculty of Biology, Medicine and Health, University of Manchester, Manchester, UK; 15 Radcliffe Department of Medicine, University of Oxford, Oxford, UK; 16 Asthma + Lung UK, London, UK; 17 Department of Clinical Research, London School of Hygiene and Tropical Medicine, London, UK; 18 Hospital for Tropical Diseases, University College London Hospital, London, UK; 19 Division of Infection and Immunity, University College London, London, UK; 20 MRC Centre for Virus Research, School of Infection and Immunity, University of Glasgow, Glasgow, UK; 21 Institute of Systems, Molecular and Integrative Biology, University of Liverpool, Liverpool, UK; 22 The Roslin Institute, University of Edinburgh, Edinburgh, UK; 23 Pandemic Science Hub, University of Edinburgh, Edinburgh, UK; 24 The Pandemic Institute, University of Liverpool, Liverpool, UK; 25 University of Manchester, Manchester, UK; 26 Intensive Care Unit, Royal Infirmary of Edinburgh, Edinburgh, UK; 27 North Bristol NHS Trust and University of Bristol, Bristol, UK; 28 University of Edinburgh, Manchester, UK; 29 King’s College Hospital NHS Foundation Trust and King’s College London, London, UK; 30 Guy’s and St Thomas’ NHS Foundation Trust, London, UK; 31 Royal Free London NHS Foundation Trust, London, UK; 32 University Hospital Birmingham NHS Foundation Trust and University of Birmingham, Birmingham, UK; 33 Stroke Association, London, UK; 34 University College London Hospital and University College London, London, UK; 35 Oxford University Hospitals NHS Foundation Trust and University of Oxford, Oxford, UK; 36 St George’s University Hospitals NHS Foundation Trust, London, UK; 37 University Hospitals of Leicester NHS Trust and University of Leicester, Leicester, UK; 38 University of Exeter, Exeter, UK; 39 University of Leicester, Leicester, UK; 40 Liverpool University Hospitals NHS Foundation Trust and University of Liverpool, Liverpool, UK; 41 Sherwood Forest Hospitals NHS Foundation Trust, Nottingham, UK; 42 Nottingham University Hospitals NHS Trust and University of Nottingham, London, UK; 43 Manchester University NHS Foundation Trust and University of Manchester, London, UK; 44 Imperial College London, London, UK; 45 Hampshire Hospitals NHS Foundation Trust, Basingstoke, UK; 46 British Heart Foundation, Birmingham, UK; 47 NHS Greater Glasgow and Clyde Health Board and University of Glasgow, Glasgow, UK; 48 University of Oxford, Oxford, UK; 49 Belfast Health and Social Care Trust and Queen’s University Belfast, Belfast, UK; 50 Airedale NHS Foundation Trust, Keighley, UK; 51 Wrightington Wigan and Leigh NHS Trust, Wigan, UK; 52 Leeds Teaching Hospitals and University of Leeds, Leeds, UK; 53 University of Liverpool, Liverpool, UK; 54 University College London, London, UK; 55 Hull University Teaching Hospitals NHS Trust and University of Hull, Hull, UK; 56 East Kent Hospitals University NHS Foundation Trust, Canterbury, UK; 57 Baillie Gifford Pandemic Science Hub, Centre for Inflammation Research, The Queen’s Medical Research Institute, University of Edinburgh, Edinburgh, UK; 58 Roslin Institute, University of Edinburgh, Edinburgh, UK; 59 Newcastle upon Tyne Hospitals NHS Foundation Trust and University of Newcastle, Newcastle upon Tyne, UK; 60 East Cheshire NHS Trust, Macclesfield, UK; 61 Sheffield Teaching NHS Foundation Trust and University of Sheffield, Sheffield, UK; 62 University of Nottingham, Nottingham, UK; 63 Wirral University Teaching Hospital, Wirral, UK; 64 MRC Human Genetics Unit, Institute of Genetics and Cancer, University of Edinburgh, Western General Hospital, Edinburgh, UK; 65 University of Swansea, Swansea, UK; 66 University of Southampton, London, UK; 67 Royal Brompton and Harefield Clinical Group, Guy’s and St Thomas’ NHS Foundation Trust, London, UK; 68 York and Scarborough NHS Foundation Trust, York, UK; 69 NHS Highland, Inverness, UK; 70 Royal Papworth Hospital NHS Foundation Trust, Cambridge, UK; 71 University Hospitals of Derby and Burton, Derby, UK; 72 NHS Lanarkshire, Hamilton, UK; 73 Cambridge University Hospitals NHS Foundation Trust, NIHR Cambridge Clinical Research Facility and University of Cambridge, Cambridge, UK; 74 Loughborough University, Loughborough, UK; 75 Betsi Cadwallader University Health Board, Bangor, UK; 76 Nottingham University Hospitals NHS Trust and University of Nottingham, Nottingham, UK; 77 King’s College London, London, UK; 78 Bradford Teaching Hospitals NHS Foundation Trust, Bradford, UK; 79 South London and Maudsley NHS Foundation Trust and King’s College London, London, UK; 80 London School of Hygiene and Tropical Medicine, London, UK; 81 Whittington Health NHS Trust, London, UK; 82 Cardiff and Vale University Health Board, Cardiff, UK; 83 Yeovil District Hospital NHS Foundation Trust, Yeovil, UK; 84 University of Birmingham, Birmingham, UK; 85 BHF Centre for Cardiovascular Science, University of Edinburgh, Edinburgh, UK; 86 University of Cambridge, Cambridge, UK; 87 NIHR Leicester Biomedical Research Centre–Respiratory Patient and Public Involvement Group, Leicester, UK; 88 Imperial College Healthcare NHS Trust and Imperial College London, London, UK; 89 Harrogate and District NHD Foundation Trust, Harrogate, UK; 90 Newcastle University/Chair of NIHR Dementia TRC, Newcastle, UK; 91 Oxford University Hospitals NHS Foundation Trust, Oxford, UK; 92 Tameside and Glossop Integrated Care NHS Foundation Trust, Ashton-under-Lyne, UK; 93 University of Oxford, Nuffield Department of Medicine, Oxford, UK; 94 University of Glasgow, Glasgow, UK; 95 United Lincolnshire Hospitals NHS Trust, Grantham, UK; 96 Department of Psychological Medicine, Institute of Psychiatry, Psychology and Neuroscience, King’s College London, London, UK; 97 University Hospital of South Manchester NHS Foundation Trust, Manchester, UK; 98 University Hospital Southampton NHS Foundation Trust and University of Southampton, Southampton, UK; 99 King’s College Hospital/Guy’s and St Thomas’ NHS FT, London, UK; 100 Barts Health NHS Trust, London, UK; 101 NHS Lothian and University of Edinburgh, Edinburgh, UK; 102 School of Cardiovascular Medicine and Sciences. King’s College London, London, UK; 103 Lewisham and Greenwich NHS Trust, London, UK; 104 Hywel Dda University Health Board, Haverfordwest, UK; 105 NHS Tayside and University of Dundee, Dundee, UK; 106 Swansea Bay University Health Board, Port Talbot, UK; 107 Faculty of Medicine, Nursing and Health Sciences, School of Biomedical Sciences, Monash University, Melbourne, Victoria Australia; 108 Rotherham NHS Foundation Trust, Rotherham, UK; 109 Salford Royal NHS Foundation Trust, Salford, UK; 110 Cwm Taf Morgannwg University Health Board, Mountain Ash, UK; 111 Borders General Hospital, NHS Borders, Melrose, UK; 112 Aneurin Bevan University Health Board, Caerleon, UK; 113 University of Exeter Medical School, Exeter, UK; 114 London North West University Healthcare NHS Trust, London, UK; 115 Alzheimer’s Research UK, Cambridge, UK; 116 Health and Care Research Wales, Cardiff, UK; 117 University of Bristol, Bristol, UK; 118 University of Sheffield, Sheffield, UK; 119 Great Western Hospital Foundation Trust, Swindon, UK; 120 Royal Devon and Exeter NHS Trust, Barnstaple, UK; 121 Kettering General Hospital NHS Trust, Kettering, UK; 122 NIHR Leicester Biomedical Research Centre, Leicester, UK; 123 University of Leeds, Leeds, UK; 124 Royal Surrey NHS Foundation Trust, Cranleigh, UK; 125 Chesterfield Royal Hospital NHS Trust, Calow, UK; 126 Long Covid Support, London, UK; 127 King’s College Hospital, NHS Foundation Trust and King’s College London, London, UK; 128 Department of Oncology and Metabolism, University of Sheffield, Sheffield, UK; 129 NIHR Office for Clinical Research Infrastructure, London, UK; 130 Asthma UK and British Lung Foundation Partnership, London, UK; 131 North Middlesex University Hospital NHS Trust, London, UK; 132 Action for Pulmonary Fibrosis, Peterborough, UK; 133 Cardiff University, National Centre for Mental Health, Cardiff, UK; 134 McPin Foundation, London, UK; 135 Roslin Institute, The University of Edinburgh, Edinburgh, UK; 136 The Hillingdon Hospitals NHS Foundation Trust, London, UK; 137 Queen Mary University of London, London, UK; 138 Swansea University, Swansea Welsh Network, Hywel Dda University Health Board, Swansea, UK; 139 Royal Infirmary of Edinburgh, NHS Lothian, Edinburgh, UK; 140 Barts Heart Centre, London, UK; 141 Barts Health NHS Trust and Queen Mary University of London, London, UK; 142 Salisbury NHS Foundation Trust, Salisbury, UK; 143 University of Newcastle, Newcastle, UK; 144 Gateshead NHS Trust, Gateshead, UK; 145 Manchester Centre for Clinical Neurosciences, Salford Royal NHS Foundation Trust, Manchester, UK; 146 Kidney Research UK, Peterborough, UK; 147 NHS Dumfries and Galloway, Dumfries, UK; 148 Swansea University, Swansea, UK; 149 MQ Mental Health Research, London, UK; 150 BHF Centre for Cardiovascular Science, Usher Institute of Population Health Sciences and Informatics, University of Edinburgh, Edinburgh, UK; 151 Shropshire Community Health NHS Trust, Shropshire, UK; 152 Somerset NHS Foundation Trust, Taunton, UK; 153 Francis Crick Institute, London, UK; 154 Manchester University NHD Foundation Trust, Manchester, UK; 155 Diabetes UK, University of Glasgow, Glasgow, UK; 156 Barnsley Hospital NHS Foundation Trust, Barnsley, UK; 157 MRC–University of Glasgow Centre for Virus Research, Glasgow, UK; 158 Diabetes UK, London, UK; 159 British Heart Foundation Centre, King’s College London, London, UK; 160 King’s College Hospital NHS Foundation Trust, London, UK; 161 University Hospitals Birmingham NHS Foundation Trust and University of Birmingham, Birmingham, UK; 162 Institute of Cardiovascular and Medical Sciences, BHF Glasgow Cardiovascular Research Centre, University of Glasgow, Glasgow, UK; 163 University College London NHS Foundation Trust, London and Barts Health NHS Trust, London, UK; 164 Northumbria University, Newcastle upon Tyne, UK; 165 Swansea University and Swansea Welsh Network, Swansea, UK; 166 DUK | NHS Digital, Salford Royal Foundation Trust, Salford, UK; 167 Queen Alexandra Hospital, Portsmouth, UK; 168 Princess Royal Hospital, Haywards Heath, UK; 169 Bassetlaw Hospital, Bassetlaw, UK; 170 Darent Valley Hospital, Dartford, UK; 171 Queen Elizabeth the Queen Mother Hospital, Margate, UK; 172 School of Informatics, University of Edinburgh, Edinburgh, UK; 173 North East and North Cumbria Ingerated, Newcastle upon Tyne, UK; 174 Section of Biomolecular Medicine, Division of Systems Medicine, Department of Metabolism, Digestion and Reproduction, Imperial College London, London, UK; 175 Section of Genomic and Environmental Medicine, Respiratory Division, National Heart and Lung Institute, Imperial College London, London, UK; 176 John Radcliffe Hospital, Oxford, UK; 177 Royal Albert Edward Infirmary, Wigan, UK; 178 Manchester Royal Infirmary, Manchester, UK; 179 MRC Human Genetics Unit, Institute of Genetics and Cancer, University of Edinburgh, Western General Hospital, Crewe Road, Edinburgh, UK; 180 Section of Molecular Virology, Imperial College London, London, UK; 181 Furness General Hospital, Barrow-in-Furness, UK; 182 Hull University Teaching Hospital Trust, Kingston upon Hull, UK; 183 Hillingdon Hospital, Hillingdon, UK; 184 St Thomas’ Hospital, London, UK; 185 Coventry and Warwickshire, Coventry, UK; 186 St Michael’s Hospital, Bristol, UK; 187 Stepping Hill Hospital, Stockport, UK; 188 Royal Liverpool University Hospital, Liverpool, UK; 189 Bristol Royal Hospital Children’s, Bristol, UK; 190 Scarborough Hospital, Scarborough, UK; 191 Golden Jubilee National Hospital, Clydebank, UK; 192 Liverpool Heart and Chest Hospital, Liverpool, UK; 193 Centre for Inflammation Research, The Queen’s Medical Research Institute, University of Edinburgh, Edinburgh, UK; 194 James Paget University Hospital, Great Yarmouth, UK; 195 Aberdeen Royal Infirmary, Aberdeen, UK; 196 Adamson Hospital, Cupar, UK; 197 Royal Devon and Exeter Hospital, Exeter, UK; 198 Worcestershire Royal Hospital, Worcester, UK; 199 ISARIC Global Support Centre, Centre for Tropical Medicine and Global Health, Nuffield Department of Medicine, University of Oxford, Oxford, UK; 200 Conquest Hospital, Hastings, UK; 201 The James Cook University Hospital, Middlesbrough, UK; 202 Dorset County Hospital, Dorchester, UK; 203 Antimicrobial Resistance and Hospital Acquired Infection Department, Public Health England, London, UK; 204 Department of Epidemiology and Biostatistics, School of Public Health, Faculty of Medicine, Imperial College London, London, UK; 205 Royal Bournemouth General Hospital, Bournemouth, UK; 206 Harrogate Hospital, Harrogate, UK; 207 Royal Blackburn Teaching Hospital, Blackburn, UK; 208 Edinburgh Clinical Research Facility, University of Edinburgh, Edinburgh, UK; 209 Torbay Hospital, Torquay, UK; 210 Northern General Hospital, Sheffield, UK; 211 Liverpool Clinical Trials Centre, University of Liverpool, Liverpool, UK; 212 Department of Infectious Disease, Imperial College London, London, UK; 213 St Georges Hospital (Tooting), London, UK; 214 Blackpool Victoria Hospital, Blackpool, UK; 215 The Royal London Hospital, London, UK; 216 MRC-University of Glasgow Centre for Virus Research, Glasgow, UK; 217 Salford Royal Hospital, Salford, UK; 218 University Hospital of North Durham, Durham, UK; 219 Norfolk and Norwich University Hospital, Norwich, UK; 220 Intensive Care Unit, Royal Infirmary Edinburgh, Edinburgh, UK; 221 Institute of Infection, Veterinary and Ecological Sciences, Faculty of Health and Life Sciences, University of Liverpool, Liverpool, UK; 222 Salisbury District Hospital, Salisbury, UK; 223 National Phenome Centre, Department of Metabolism, Digestion and Reproduction, Imperial College London, London, UK; 224 Section of Bioanalytical Chemistry, Department of Metabolism, Digestion and Reproduction, Imperial College London, London, UK; 225 Guy’s and St Thomas’, NHS Foundation Trust, London, UK; 226 The Royal Oldham Hospital, Oldham, UK; 227 European Genomic Institute for Diabetes, Institut Pasteur de Lille, Lille University Hospital, University of Lille, Lille, France; 228 McGill University and Genome Quebec Innovation Centre, Montreal, Qeubec Canada; 229 National Infection Service, Public Health England, London, UK; 230 Hereford Count Hospital, Hereford, UK; 231 Southampton General Hospital, Southampton, UK; 232 Northampton General Hospital, Northampton, UK; 233 University Hospital of Wales, Cardiff, UK; 234 University Hospitals Bristol NHS Foundation Trust, Bristol, UK; 235 Liverpool School of Tropical Medicine, Liverpool, UK; 236 Leighton Hospital, Crewe, UK; 237 Manor Hospital, Walsall, UK; 238 Scunthorpe Hospital, Scunthorpe, UK; 239 Cambridge University Hospital, Cambridge, UK; 240 West Suffolk NHS Foundation Trust, Bury St Edmunds, UK; 241 Basingstoke and North Hampshire Hospital, Basingstoke, UK; 242 North Cumberland Infirmary, Carlisle, UK; 243 Paediatric Liver, GI and Nutrition Centre and MowatLabs, King’s College Hospital, London, UK; 244 Institute of Liver Studies, King’s College London, London, UK; 245 Institute of Microbiology and Infection, University of Birmingham, Birmingham, UK; 246 Department of Molecular and Clinical Cancer Medicine, University of Liverpool, Liverpool, UK; 247 Institute for Global Health, University College London, London, UK; 248 NIHR Health Protection Research Unit, Institute of Infection, Veterinary and Ecological Sciences, Faculty of Health and Life Sciences, University of Liverpool, Liverpool, UK; 249 Warwick Hospital, Warwick, UK; 250 Birmingham Children’s Hospital, Birmingham, UK; 251 Nottingham City Hospital, Nottingham, UK; 252 Glangwili Hospital Child Health Section, Carmarthen, UK; 253 Alder Hey Children’s Hospital, Liverpool, UK; 254 Department of Infectious Diseases, Queen Elizabeth University Hospital, Glasgow, UK; 255 Bronglais General Hospital, Aberystwyth, UK; 256 Worthing Hospital, Worthing, UK; 257 Centre for Tropical Medicine and Global Health, Nuffield Department of Medicine, University of Oxford, Oxford, UK; 258 Rotheram District General Hospital, Rotheram, UK; 259 Virology Reference Department, National Infection Service, Public Health England, Colindale Avenue, London, UK; 260 Royal Free Hospital, London, UK; 261 Homerton Hospital, London, UK; 262 Airedale Hospital, Airedale, UK; 263 Basildon Hospital, Basildon, UK; 264 The Christie NHS Foundation Trust, Manchester, UK; 265 University Hospital Lewisham, London, UK; 266 The Whittington Hospital, London, UK; 267 Southmead Hospital, Bristol, UK; 268 Sheffield Childrens Hospital, Sheffield, UK; 269 Royal United Hospital, Bath, UK; 270 Department of Pharmacology, University of Liverpool, Liverpool, UK; 271 Nuffield Department of Medicine, Peter Medawar Building for Pathogen Research, University of Oxford, Oxford, UK; 272 Translational Gastroenterology Unit, Nuffield Department of Medicine, University of Oxford, Oxford, UK; 273 Public Health Scotland, Edinburgh, UK; 274 Western General Hospital, Edinburgh, UK; 275 Southend University Hospital NHS Foundation Trust, Southend-on-Sea, UK; 276 Hinchingbrooke Hospital, Huntingdon, UK; 277 Royal Preston Hospital, Fulwood, UK; 278 University Hospital (Coventry), Coventry, UK; 279 The Walton Centre, Liverpool, UK; 280 ISARIC, Global Support Centre, COVID-19 Clinical Research Resources, Epidemic diseases Research Group, Oxford (ERGO), University of Oxford, Oxford, UK; 281 Centre for Health Informatics, Division of Informatics, Imaging and Data Science, School of Health Sciences, Faculty of Biology, Medicine and Health, University of Manchester, Manchester Academic Health Science Centre, Manchester, UK; 282 Hull Royal Infirmary, Hull, UK; 283 Nottingham University Hospitals NHS Trust:, Nottingham, UK; 284 Darlington Memorial Hospital, Darlington, UK; 285 Queen Elizabeth Hospital (Gateshead), Gateshead, UK; 286 Warrington Hospital, Warrington, UK; 287 Bristol Royal Hospital for Children, Bristol, UK; 288 St Mary’s Hospital (Isle of Wight), Isle of Wight, UK; 289 The Tunbridge Wells Hospital, Royal Tunbridge Wells, UK; 290 Huddersfield Royal, Huddersfield, UK; 291 Countess of Chester Hospital, Liverpool, UK; 292 Frimley Park Hospital, Frimley, UK; 293 Nuffield Department of Medicine, John Radcliffe Hospital, Oxford, UK; 294 Department of Microbiology/Infectious Diseases, Oxford University Hospitals NHS Foundation Trust, John Radcliffe Hospital, Oxford, UK; 295 MRC Human Genetics Unit, MRC Institute of Genetics and Molecular Medicine, University of Edinburgh, Edinburgh, UK; 296 St James University Hospital, Leeds, UK; 297 Arrowe Park Hospital, Birkenhead, UK; 298 Great Ormond Street Hospital, London, UK; 299 Royal Shrewsbury Hospital, Shrewsbury, UK; 300 Addenbrookes Hospital, Cambridge, UK; 301 Institute of Infection, Veterinary and Ecological Sciences, University of Liverpool, Liverpool, UK; 302 East Surrey Hospital (Redhill), Redhill, UK; 303 Burton Hospital, Burton, UK; 304 Peterborough City Hospital, Peterborough, UK; 305 Kent and Canterbury Hospital, Canterbury, UK; 306 Weston Area General Trust, Bristol, UK; 307 Bedfordshire Hospital, Bedfordshire, UK; 308 Glasgow Royal Infirmary, Glasgow, UK; 309 Macclesfield General Hospital, Macclesfield, UK; 310 Derbyshire Healthcare, Derbyshire, UK; 311 Chelsea and Westminster Hospital, London, UK; 312 Watford General Hospital, Watford, UK; 313 EPCC, University of Edinburgh, Edinburgh, UK; 314 Section of Biomolecular Medicine, Division of Systems Medicine, Department of Metabolism, Digestion and Reproduction, London, UK; 315 Imperial College Healthcare NHS Trust: London, London, UK; 316 Division of Systems Medicine, Department of Metabolism, Digestion and Reproduction, Imperial College London, London, UK; 317 Prince Philip Hospital, Llanelli, UK; 318 George Eliot Hospital – Acute Services, Nuneaton, UK; 319 Molecular and Clinical Cancer Medicine, Institute of Systems, Molecular and Integrative Biology, University of Liverpool, Liverpool, UK; 320 Clatterbridge Cancer Centre NHS Foundation Trust, Liverpool, UK; 321 Kettering General Hospital, Kettering, UK; 322 University Hospitals of North Midlands NHS Trust, North Midlands, UK; 323 Russells Hall Hospital, Dudley, UK; 324 Harefield Hospital, Harefield, UK; 325 Lister Hospital, Lister, UK; 326 Musgrove Park Hospital, Taunton, UK; 327 Kingston Hospital, Kingston, UK; 328 Queen’s Hospital, Romford, UK; 329 Southport and Formby District General Hospital, Southport, UK; 330 St George’s University of London, London, UK; 331 King’s College Hospital (Denmark Hill), London, UK; 332 Centre for Clinical Infection and Diagnostics Research, Department of Infectious Diseases, School of Immunology and Microbial Sciences, King’s College London, London, UK; 333 Department of Infectious Diseases, Guy’s and St Thomas’ NHS Foundation Trust, London, UK; 334 The Clatterbridge Cancer Centre NHS Foundation, Bebington, UK; 335 The Great Western Hospital, Swindon, UK; 336 Ninewells Hospital, Dundee, UK; 337 Institute of Evolutionary Biology, University of Edinburgh, Edinburgh, UK; 338 Poole Hospital NHS Trust, Poole, UK; 339 William Harvey Hospital, Ashford, UK; 340 King’s Mill Hospital, Sutton-in-Ashfield, UK; 341 Liverpool Women’s Hospital, Liverpool, UK; 342 Pinderfields Hospital, Wakefield, UK; 343 North Devon District Hospital, Barnstaple, UK; 344 Queen Elizabeth Hospital, Birmingham, UK; 345 Tameside General Hospital, Ashton-under-Lyne, UK; 346 City Hospital (Birmingham), Birmingham, UK; 347 Department of Pediatrics and Virology, St Mary’s Medical School Bldg, Imperial College London, London, UK; 348 The Newcastle Upon Tyne Hospitals NHS Foundation Trust, Newcastle Upon Tyne, UK; 349 NHS Greater Glasgow and Clyde, Glasgow, UK; 350 Respiratory Medicine, Institute in The Park, University of Liverpool, Alder Hey Children’s Hospital, Liverpool, UK; 351 Broomfield Hospital, Broomfield, UK; 352 Stoke Mandeville, UK; 353 University Hospital of North Tees, Stockton-on-Tees, UK; 354 Institute of Translational Medicine, University of, Liverpool, Merseyside, UK; 355 Royal Manchester Children’s Hospital, Manchester, UK; 356 New Cross Hospital, Wolverhampton, UK; 357 Bedford Hospital, Bedford, UK; 358 Colchester General Hospital, Colchester, UK; 359 University Hospital Birmingham NHS Foundation Trust, Birmingham, UK; 360 Walton Centre NHS Foundation Trust, Liverpool, UK; 361 Chesterfield Royal Hospital, Calow, UK; 362 MRC Centre for Molecular Bacteriology and Infection, Imperial College London, London, UK; 363 Princess Alexandra Hospital, Harlow, UK; 364 Milton Keynes Hospital, Eaglestone, UK; 365 Division of Structural Biology, The Wellcome Centre for Human Genetics, University of Oxford, Oxford, UK; 366 Royal Bolton Hopital, Farnworth, UK; 367 Department of Medicine, University of Cambridge, Cambridge, UK; 368 Department of Child Life and Health, University of Edinburgh, Edinburgh, UK; 369 Royal Gwent (Newport), Newport, UK; 370 The Royal Marsden Hospital (London), London, UK; 371 Blood Borne Virus Unit, Virus Reference Department, National Infection Service, Public Health England, London, UK; 372 Transfusion Microbiology, National Health Service Blood and Transplant, London, UK; 373 Department of Medicine, Imperial College London, London, UK; 374 Queen Victoria Hospital (East Grinstead), East Grinstead, UK; 375 Leeds Teaching Hospitals NHS Trust, Leeds, UK; 376 Royal Stoke University Hospital, Stoke-on-Trent, UK; 377 Whiston Hospital, Rainhill, UK; 378 Tropical and Infectious Disease Unit, Royal Liverpool University Hospital, Liverpool, UK; 379 Croydon University Hospital, Thornton Heath, UK; 380 Gloucester Royal, Gloucester, UK; 381 West Hertfordshire Teaching Hospitals NHS Trust, Hertfordshire, UK; 382 North Middlesex Hospital, London, UK; 383 Medway Maritime Hospital, Gillingham, UK; 384 Royal Papworth Hospital Everard, Cambridge, UK; 385 Derriford (Plymouth), Plymouth, UK; 386 St Helier Hospital, Sutton, UK; 387 Royal Berkshire Hospital, Reading, UK; 388 Royal Liverpool Hospital, Liverpool, UK; 389 Bradford Royal infirmary, Bradford, UK; 390 Central Middlesex, London, UK; 391 Royal Cornwall Hospital (Tresliske), Truro, UK; 392 North Bristol NHS Trust, Bristol, UK; 393 St. Peter’s Hospital, Runnymede, UK; 394 Leicester Royal Infirmary, Leicester, UK; 395 Grantham and District Hospital, Grantham, UK; 396 Aintree University Hospital, Liverpool, UK; 397 North Tyneside General Hospital, North Shields, UK; 398 Queen Elizabeth Hospital, King’s Lynn, UK

**Keywords:** Inflammasome, Inflammation, Innate immunity

## Abstract

One in ten severe acute respiratory syndrome coronavirus 2 infections result in prolonged symptoms termed long coronavirus disease (COVID), yet disease phenotypes and mechanisms are poorly understood^1^. Here we profiled 368 plasma proteins in 657 participants ≥3 months following hospitalization. Of these, 426 had at least one long COVID symptom and 233 had fully recovered. Elevated markers of myeloid inflammation and complement activation were associated with long COVID. IL-1R2, MATN2 and COLEC12 were associated with cardiorespiratory symptoms, fatigue and anxiety/depression; MATN2, CSF3 and C1QA were elevated in gastrointestinal symptoms and C1QA was elevated in cognitive impairment. Additional markers of alterations in nerve tissue repair (SPON-1 and NFASC) were elevated in those with cognitive impairment and SCG3, suggestive of brain–gut axis disturbance, was elevated in gastrointestinal symptoms. Severe acute respiratory syndrome coronavirus 2-specific immunoglobulin G (IgG) was persistently elevated in some individuals with long COVID, but virus was not detected in sputum. Analysis of inflammatory markers in nasal fluids showed no association with symptoms. Our study aimed to understand inflammatory processes that underlie long COVID and was not designed for biomarker discovery. Our findings suggest that specific inflammatory pathways related to tissue damage are implicated in subtypes of long COVID, which might be targeted in future therapeutic trials.

## Abstract (web summary)

Openshaw and colleagues find myeloid inflammation and complement activation signatures in patients with long COVID who were previously hospitalized.

## Main

One in ten severe acute respiratory syndrome coronavirus 2 (SARS-CoV-2) infections results in post-acute sequelae of coronavirus disease 2019 (PASC) or long coronavirus disease (COVID), which affects 65 million people worldwide^1^. Long COVID (LC) remains common, even after mild acute infection with recent variants^2^, and it is likely LC will continue to cause substantial long-term ill health, requiring targeted management based on an understanding of how disease phenotypes relate to underlying mechanisms. Persistent inflammation has been reported in adults with LC^1,3^, but studies have been limited in size, timing of samples or breadth of immune mediators measured, leading to inconsistent or absent associations with symptoms. Markers of oxidative stress, metabolic disturbance, vasculoproliferative processes and IFN-, NF-κB- or monocyte-related inflammation have been suggested^3–6^.

The PHOSP-COVID study, a multicenter United Kingdom study of patients previously hospitalized with COVID-19, has reported inflammatory profiles in 626 adults with health impairment after COVID-19, identified through clustering. Elevated IL-6 and markers of mucosal inflammation were observed in those with severe impairment compared with individuals with milder impairment^7^. However, LC is a heterogeneous condition that may be a distinct form of health impairment after COVID-19, and it remains unclear whether there are inflammatory changes specific to LC symptom subtypes. Determining whether activated inflammatory pathways underlie all cases of LC or if mechanisms differ according to clinical presentation is essential for developing effective therapies and has been highlighted as a top research priority by patients and clinicians^8^.

**Fig. 1:**
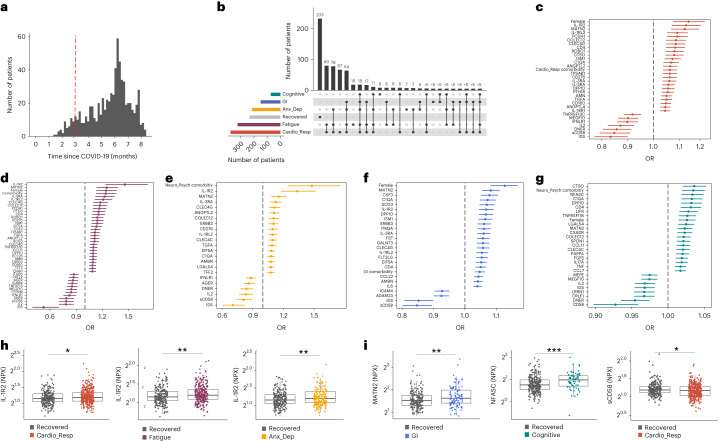
Subtypes of LC are associated with distinct inflammatory profiles. **a**, Distribution of time from COVID-19 hospitalization at sample collection. All samples were cross-sectional. The vertical red line indicates the 3 month cutoff used to define our final cohort and samples collected before 3 months were excluded. **b**, An UpSet plot describing pooled LC groups. The horizontal colored bars represent the number of patients in each symptom group: cardiorespiratory (Cardio_Resp), fatigue, cognitive, GI and anxiety/depression (Anx_Dep). Vertical black bars represent the number of patients in each symptom combination group. To prevent patient identification, where less than five patients belong to a combination group, this has been represented as ‘<5’. The recovered group (*n* = 233) were used as controls. **c**–**g**, Forest plots of Olink protein concentrations (NPX) associated with Cardio_Resp (*n* = 365) (**c**), fatigue (n = 314) (**d**), Anx_Dep (*n* = 202) (**e**), GI (*n* = 124) (**f**) and cognitive (*n* = 60) (**g**). Neuro_Psych, neuropsychiatric. The error bars represent the median accuracy of the model. **h**,**i**, Distribution of Olink values (NPX) for IL-1R2 (**h**) and MATN2, neurofascin and sCD58 (**i**) measured between symptomatic and recovered individuals in recovered (*n* = 233), Cardio_Resp (*n* = 365), fatigue (*n* = 314) and Anx_Dep (*n* = 202) groups (**h**) and MATN2 in GI (*n* = 124), neurofascin in cognitive (*n* = 60) and sCD58 in Cardio_Resp and recovered groups (**i**). The box plot center line represents the median, the boundaries represent IQR and the whisker length represents 1.5× IQR. The median values were compared between groups using two-sided Wilcoxon signed-rank test, *P < 0.05, **P < 0.01, ***P < 0.001 and ****P < 0.0001.

**Extended Data Fig. 1:**
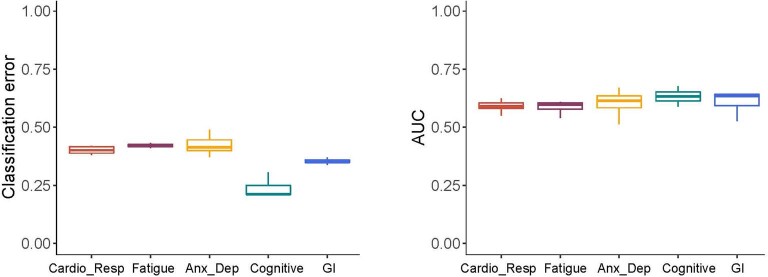
Penalized logistic regression performance. Graphs show classification error and Area under curve (AUC) from the 50 repeats tenfold nested cross-validation used to optimise and assess the performance of PLR testing associations with each LC outcome relative to Recovered (n = 233): Cardio_Resp (n = 398), Fatigue (n = 384), Anxiety/Depression (n = 202), GI (n = 132), (**e**) Cognitive (n = 6). The distributions of classification error and area under curve (AUC) from the nested cross-validation are shown. Box plot centre line represents the Median and boundaries of the box represent interquartile range (IQR), the whisker length represent 1.5xIQR.

In this Letter, in a prospective multicenter study, we measured 368 plasma proteins in 657 adults previously hospitalized for COVID-19 (Fig. 1a and Table 1). Individuals in our cohort experienced a range of acute COVID-19 severities based on World Health Organization (WHO) progression scores^9^; WHO 3–4 (no oxygen support, *n* = 133 and median age of 55 years), WHO 5–6 (oxygen support, *n* = 353 and median age of 59 years) and WHO 7–9 (critical care, *n* = 171 and median age of 57 years). Participants were hospitalized for COVID-19 ≥3 months before sample collection (median 6.1 months, interquartile range (IQR) 5.1–6.8 months and range 3.0–8.3 months) and confirmed clinically (*n* = 36/657) or by PCR (*n* = 621/657). Symptom data indicated 233/657 (35%) felt fully recovered at 6 months (hereafter ‘recovered’) and the remaining 424 (65%) reported symptoms consistent with the WHO definition for LC (symptoms ≥3 months post infection^10^). Given the diversity of LC presentations, patients were grouped according to symptom type (Fig. 1b). Groups were defined using symptoms and health deficits that have been commonly reported in the literature^1^ (Methods). A multivariate penalized logistic regression model (PLR) was used to explore associations of clinical covariates and immune mediators at 6 months between recovered patients (*n* = 233) and each LC group (cardiorespiratory symptoms, cardioresp, *n* = 398, Fig. 1c; fatigue, *n* = 384, Fig. 1d; affective symptoms, anxiety/depression, *n* = 202, Fig. 1e; gastrointestinal symptoms, GI, *n* = 132, Fig. 1f; and cognitive impairment, cognitive, *n* = 61, Fig. 1g). Women (*n* = 239) were more likely to experience CardioResp (odds ratio (OR 1.14), Fatigue (OR 1.22), GI (OR 1.13) and Cognitive (OR 1.03) outcomes (Fig. 1c,d,f,g). Repeated cross-validation was used to optimize and assess model performance (Methods and Extended Data Fig. 1). Pre-existing conditions, such as chronic lung disease, neurological disease and cardiovascular disease (Supplementary Table 1), were associated with all LC groups (Fig. 1c–g). Age, C-reactive protein (CRP) and acute disease severity were not associated with any LC group (Table 1).

**Table 1:** Cohort demographics

		GI	Fatigue	Cardiorespiratory	Anxiety/depression	Cognitive impairment	Recovered	*P* value
Age at admission	Years (s.d.)	57.72 (11.48)	56.57 (11.07)	57.08 (11.37)	56.36 (10.84)	59.24 (12.82)	58.92 (13.72)	0.046 *
Sex	Female *N* (%)	68 (53%)	143 (47%)	161 (43%)	89 (45%)	24 (42%)	55 (27%)	1.69 × 10^−6^****
Ethnicity	White	110	300	331	193	50	197	0.09 NS
	South Asian	14	26	38	16	7	46	
	Black	8	16	25	11	7	10	
	Mixed/Other	8	24	22	16	7	17	
WHO clinical progression scale for acute COVID-19	Class 3–4	41	83	88	45	18	45	0.28 NS
	Class 5	45	107	124	74	21	115	
	Class 6	27	78	89	55	11	57	
	Class 7–9	27	98	115	62	21	50	
CRP	Mean (s.d.)	5.33 (5.42)	5.47 (7.17)	5.17 (6.82)	5.79 (8.12)	4.58 (5.78)	4.75 (10.38)	0.76 NS
Length of hospitalization	Days (s.d.)	12.04 (14.3)	14.59 (18.41)	15.39 (19.96)	14.57 (17.76)	14.95 (16.01)	12.5 (15.73)	0.0047**
Steroid^a^	% Yes	34%	35%	37%	38%	33%	29%	0.294 NS
Remdesivir^a^	% Yes	4%	3%	4%	2%	3%	3%	0.725 NS
Comorbidities	Mean (s.d.)	2.9 (2.62)	2.675 (2.3)	2.553 (2.24)	2.911 (2.47)	2.493 (2.17)	1.554 (1.67)	9.92 × 10^−10^****

The demographics of each symptom group and recovered controls are shown. The WHO clinical progression scale was used to classify acute COVID-19 severity: class 3–4: no oxygen requirement; class 5: oxygen therapy; class 6: noninvasive ventilation or high-flow nasal oxygen and class 7–9: organ support. Differences between groups were compared using chi-squared, two-way Kruskal–Wallis or two-way analysis of variance as appropriate. Data are *n* (%) or mean (s.d.). CRP levels represent those measured contemporaneously with clinical data collected in this study.

^a^Denotes treatment given during acute illness.

To study the association of peripheral inflammation with symptoms, we analyzed cross-sectional data collected approximately 6 months after hospitalizations. We measured 368 immune mediators from plasma collected contemporaneously with symptom data. Mediators suggestive of myeloid inflammation were associated with all symptoms (Fig. 1c–h). Elevated IL-1R2, an IL-1 receptor expressed by monocytes and macrophages modulating inflammation^11^ and MATN2, an extracellular matrix protein that modulates tissue inflammation through recruitment of innate immune cells^12^, were associated with cardioresp (IL-1R2 OR 1.14, Fig. 1c,h), fatigue (IL-1R2 OR 1.45, Fig. 1d,h), anxiety/depression (IL-1R2 OR 1.34. Fig. 1e,h) and GI (MATN2 OR 1.08, Fig. 1f). IL-3RA, an IL-3 receptor, was associated with cardioresp (OR 1.07, Fig. 1c), fatigue (OR 1.21, Fig. 1d), anxiety/depression (OR 1.12, Fig. 1e) and GI (OR 1.06, Fig. 1f) groups, while CSF3, a cytokine promoting neutrophilic inflammation^13^, was elevated in cardioresp (OR 1.06, Fig. 1c), fatigue (OR 1.12, Fig. 1d) and GI (OR 1.08, Fig. 1f).

Elevated COLEC12, which initiates inflammation in tissues by activating the alternative complement pathway^14^, associated with cardioresp (OR 1.09, Fig. 1c), fatigue (OR 1.19, Fig. 1d) and anxiety/depression (OR 1.11, Fig. 1e), but not with GI (Fig. 1f) and only weakly with cognitive (OR 1.02, Fig. 1g). C1QA, a degradation product released by complement activation^15^ was associated with GI (OR 1.08, Fig. 1f) and cognitive (OR 1.03, Fig. 1g). C1QA, which is known to mediate dementia-related neuroinflammation^16^, had the third strongest association with cognitive (Fig. 1g). These observations indicated that myeloid inflammation and complement activation were associated with LC.

**Extended Data Fig. 2:**
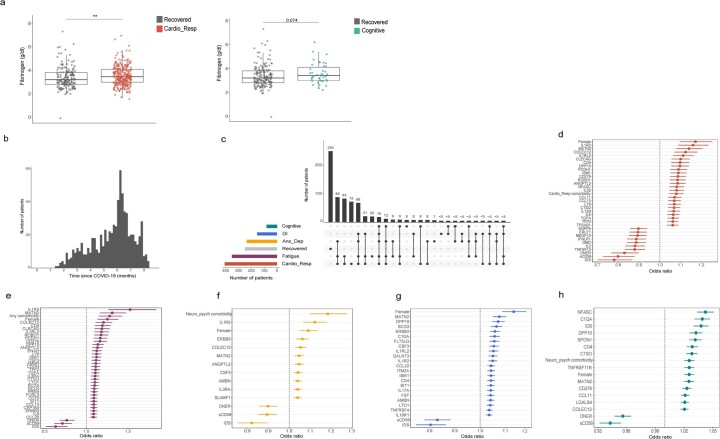
Associations with long COVID symptoms in full study cohort. (**a**) Fibrinogen levels at 6 months were compared between pooled LC cases (n = 295) and Recovered (n = 233) and between the Cognitive group (n = 41) and Recovered (n = 233). Box plot centre line represent the Median and boundaries of the box represent interquartile range (IQR), the whisker length represents 1.5xIQR, any outliers beyond the whisker range are shown as individual dots. Median differences were compared using two-sided Wilcoxon signed-rank test *=*p* < 0·05, **=*p* < 0·01, ***=*p* < 0·001, ****=*p* < 0·0001. Unadjusted p-values are reported. **b**) Distribution of time from COVID-19 hospitalisation at sample collection applying CDC and NICE definitions of LC (n = 719) (**c**) Upset plot of symptom groups. Horizontal coloured bars represent the number of patients in each symptom group: Cardiorespiratory (Cardio_Resp), Fatigue, Cognitive, Gastrointestinal (GI) and Anxiety/Depression (Anx_Dep). Vertical black bars represent the number of patients in each symptom combination group. To prevent patient identification, where less than 5 patients belong to a combination group, this has been represented as ‘<5’. The Recovered group (n = 250) were used as controls. Forest plots show Olink protein concentrations (NPX) associated with (**d**) Cardio_Resp (n = 398), (**e**) Fatigue (n = 342), (**f**) Anx_Dep (n = 219), (**g**) GI (n = 134), and (**h**) Cognitive (n = 65). Error bars represent the median accuracy of the model.

Increased expression of DPP10 and SCG3 was observed in the GI group compared with recovered (DPP10 OR 1.07 and SCG3 OR 1.08, Fig. 1f). DPP10 is a membrane protein that modulates tissue inflammation, and increased *DPP10* expression is associated with inflammatory bowel disease^17,18^, suggesting that GI symptoms may result from enteric inflammation. Elevated SCG3, a multifunctional protein that has been associated with irritable bowel syndrome^19^, suggested that noninflammatory disturbance of the brain–gut axis or dysbiosis, may occur in the GI group. The cognitive group was associated with elevated CTSO (OR 1.04), NFASC (OR 1.03) and SPON-1 (OR 1.02, Fig. 1g,i). NFASC and SPON-1 regulate neural growth^20,21^, while CTSO is a cysteine proteinase supporting tissue turnover^22^. The increased expression of these three proteins as well as C1QA and DPP10 in the cognitive group (Fig. 1g) suggested neuroinflammation and alterations in nerve tissue repair, possibly resulting in neurodegeneration. Together, our findings indicated that complement activation and myeloid inflammation were common to all LC groups, but subtle differences were observed in the GI and cognitive groups, which may have mechanistic importance. Acutely elevated fibrinogen during hospitalization has been reported to be predictive of LC cognitive deficits^23^. We found elevated fibrinogen in LC relative to recovered (Extended Data Fig. 2a; *P* = 0.0077), although this was not significant when restricted to the cognitive group (*P* = 0.074), supporting our observation of complement pathway activation in LC and in keeping with reports that complement dysregulation and thrombosis drive severe COVID-19 (ref. ^24^).

Elevated sCD58 was associated with lower odds of all LC symptoms and was most pronounced in cardioresp (OR 0.85, Fig. 1c,i), fatigue (OR 0.80, Fig. 1d) and anxiety/depression (OR 0.83, Fig. 1e). IL-2 was negatively associated with the cardioresp (Fig. 1c, OR 0.87), fatigue (Fig. 1d, OR 0.80), anxiety/depression (Fig. 1e, OR 0.84) and cognitive (Fig. 1g, OR 0.96) groups. Both IL-2 and sCD58 have immunoregulatory functions^25,26^. Specifically, sCD58 suppresses IL-1- or IL-6-dependent interactions between CD2^+^ monocytes and CD58^+^ T or natural killer cells^26^. The association of sCD58 with recovered suggests a central role of dysregulated myeloid inflammation in LC. Elevated markers of tissue repair, IDS and DNER^27,28^, were also associated with recovered relative to all LC groups (Fig. 1c–g). Taken together, our data suggest that suppression of myeloid inflammation and enhanced tissue repair were associated with recovered, supporting the use of immunomodulatory agents in therapeutic trials^29^ (Supplementary Table 2).

**Extended Data Fig. 3:**
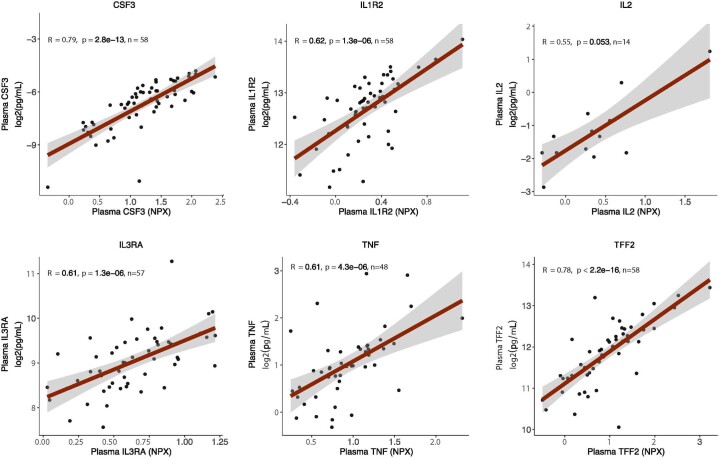
Validation of olink measurements using conventional assays in plasma. Olink measured protein (NPX) were compared to chemiluminescence assays (ECL or ELISA, log2[pg/mL]) to validate our findings, where contemporaneously collected plasma samples were available (n = 58). Results from key mediators associated with LC groups were validated: CSF3, IL1R2, IL2, IL3RA, TNFa, TFF2. *R*= spearman rank correlation coefficient and shaded areas indicated the 95% confidence interval. Samples that fell below the lower limit of detection for a given assay were excluded and the ‘n’ value on each panel indicates the number of samples above this limit.

**Extended Data Fig. 4:**
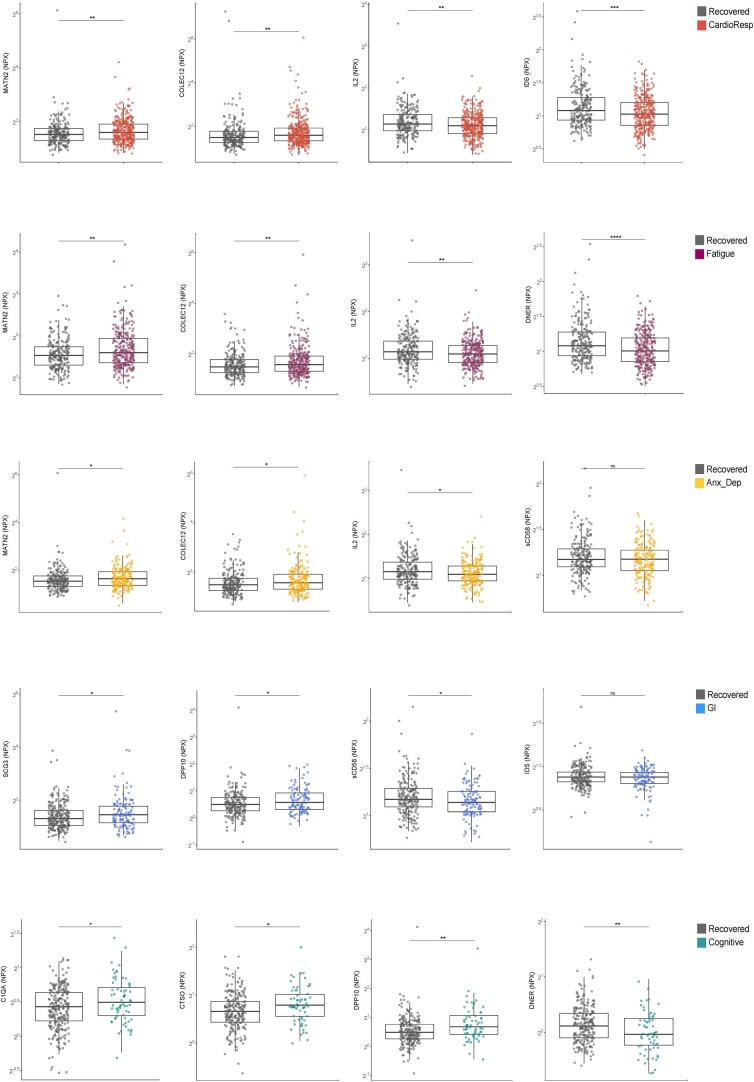
Univariate analysis of proteins associated with each symptom. Olink measured plasma protein levels (NPX) compared between LC groups (Cardio_Resp, n = 398, Fatigue n = 384, Anxiety/Depression, n = 202, GI, n = 132 and Cognitive, n = 60) and Recovered (n = 233). Proteins identified by PLR were compared between groups. Median differences were compared using two-sided Wilcoxon signed-rank test. * = p < 0·05, ** = p < 0·01, *** = p < 0·001, ****= p < 0·0001 after FDR adjustment. Box plot centre line represent the Median and boundaries of the box represent interquartile range (IQR), the whisker length represents 1.5xIQR, any outliers beyond the whisker range are shown as individual dots.

**Extended Data Fig. 5:**
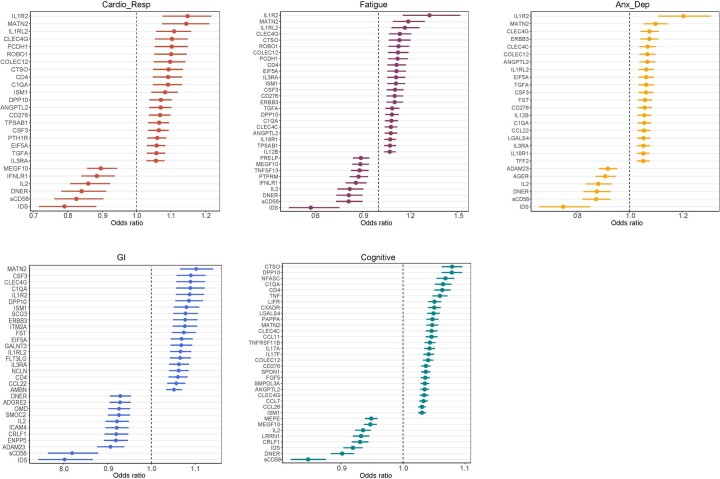
Unadjusted Penalised Logistic Regression. Olink measured proteins (NPX) and their association with Cardio_Resp (n = 398), Fatigue (n = 342), Anx_Dep (n = 219), GI (n = 134), and Cognitive (n = 65). Forest plots show odds of each LC outcome vs Recovered (n = 233), using PLR without adjusting for clinical co-variates. Error bars represent the median accuracy of the model.

**Extended Data Fig. 6:**
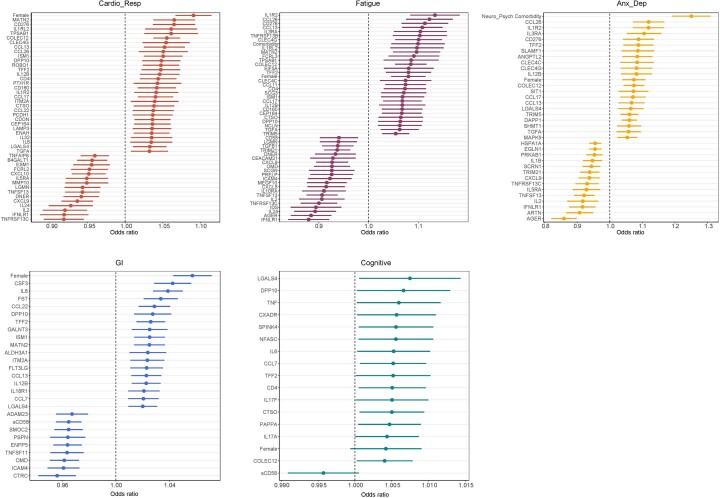
Partial Least Squares analysis. Olink measured proteins (NPX) and their association with Cardio_Resp (n = 398), Fatigue (n = 342), Anx_Dep (n = 219), GI (n = 134), and Cognitive (n = 65) groups. Forest plots show odds of LC outcome vs Recovered (n = 233), using PLS analysis. Error bars represent the standard error of the coefficient estimate.

We next sought to validate the experimental and analytical approaches used. Although Olink has been validated against other immunoassay platforms, showing superior sensitivity and specificity^30,31^, we confirmed the performance of Olink against chemiluminescent immunoassays within our cohort. We performed chemiluminescent immunoassays on plasma from a subgroup of 58 participants (recovered *n* = 13 and LC *n* = 45). There were good correlations between results from Olink (normalized protein expression (NPX)) and chemiluminescent immunoassays (pg ml^−1^) for CSF3, IL-1R2, IL-3RA, TNF and TFF2 (Extended Data Fig. 3). Most samples did not have concentrations of IL-2 detectable using a mesoscale discovery chemiluminescent assay, limiting this analysis to 14 samples (recovered *n* = 4, LC *n* = 10, *R* = 0.55 and *P* = 0.053, Extended Data Fig. 3). We next repeated our analysis using alternative definitions of LC. The Centers for Disease Control and Prevention and National Institute for Health and Care Excellence definitions for LC include symptoms occurring 1 month post infection^32,33^. Using the 1 month post-infection definition included 62 additional participants to our analysis (recovered *n* = 21, 3 females and median age 61 years and LC *n* = 41, 15 females and median age 60 years, Extended Data Fig. 2c) and found that inflammatory associations with each LC group were consistent with our analysis based on the WHO definition (Extended Data Fig. 2d–h). Finally, to validate the analytical approach (PLR) we examined the distribution of data, prioritizing proteins that were most strongly associated with each LC/recovered group (IL-1R2, MATN2, NFASC and sCD58). Each protein was significantly elevated in the LC group compared with recovered (Fig. 1h,i and Extended Data Fig. 4), consistent with the PLR. Alternative regression approaches (unadjusted regression models and partial least squares, PLS) reported results consistent with the original analysis of protein associations and LC outcome in the WHO-defined cohort (Fig. 1c–g, Supplementary Table 3 and Extended Data Figs. 5 and 6). The standard errors of PLS estimates were wide (Extended Data Fig. 6), consistent with previous demonstrations that PLR is the optimal method to analyze high-dimensional data where variables may have combined effects^34^. As inflammatory proteins are often colinear, working in-tandem to mediate effects, we prioritized PLR results to draw conclusions.

**Fig. 2:**
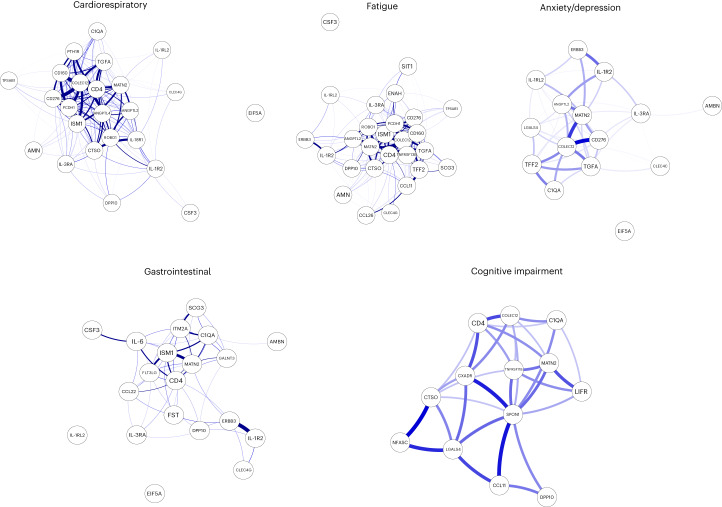
Network analyses define key immune mediators in LC symptom groups. Network analysis of Olink mediators associated with cardioresp (*n* = 365), fatigue (*n* = 314), anxiety/depression (*n* = 202), GI (*n* = 124) and cognitive groups (*n* = 60). Each node corresponds to a protein mediator identified by PLR. The edges (blue lines) were weighted according to the size of Spearman’s rank correlation coefficient between proteins. All edges represent positive and significant correlations (*P* < 0.05) after FDR adjustment.

**Extended Data Fig. 7:**
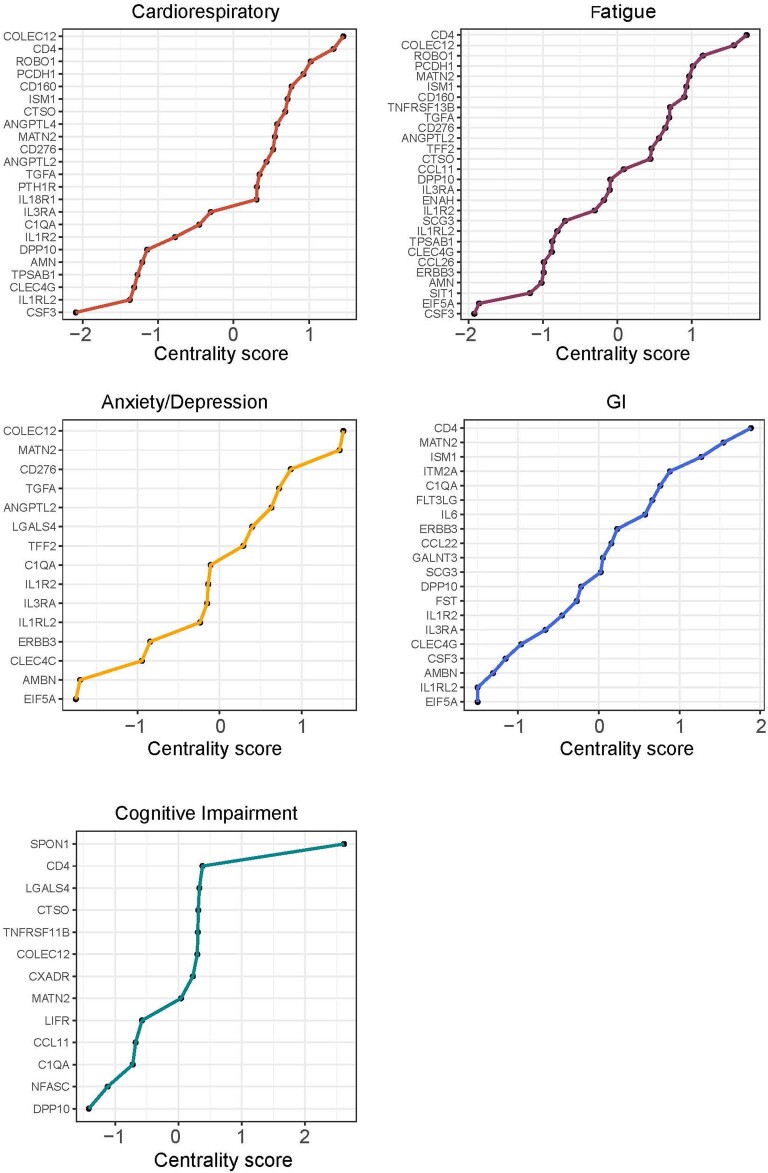
Network analysis centrality. Each graph shows the centrality score for each Olink measured protein (NPX) found to have significant associations with other proteins that were elevated in the Cardio_Resp (n = 398), Fatigue (n = 342), Anx_Dep (n = 219), GI (n = 134), and Cognitive (n = 65) groups relative to Recovered (n = 233).

To explore the relationship between inflammatory mediators associated with different LC symptoms, we performed a network analysis of Olink mediators highlighted by PLR within each LC group. COLEC12 and markers of endothelial and mucosal inflammation (MATN2, PCDH1, ROBO1, ISM1, ANGPTL2, TGF-α and TFF2) were highly correlated within the cardioresp, fatigue and anxiety/depression groups (Fig. 2 and Extended Data Fig. 7). Elevated PCDH1, an adhesion protein modulating airway inflammation^35^, was highly correlated with other inflammatory proteins associated with the cardioresp group (Fig. 2), suggesting that systemic inflammation may arise from the lung in these individuals. This was supported by increased expression of IL-3RA, which regulates innate immune responses in the lung through interactions with circulating IL-3 (ref. ^36^), in fatigue (Figs. 1d and 2), which correlated with markers of tissue inflammation, including PCDH1 (Fig. 2). MATN2 and ISM1, mucosal proteins that enhance inflammation^37,38^, were highly correlated in the GI group (Fig. 2), highlighting the role of tissue-specific inflammation in different LC groups. SCG3 correlated less closely with mediators in the GI group (Fig. 2), suggesting that the brain–gut axis may contribute separately to some GI symptoms. SPON-1, which regulates neural growth^21^, was the most highly correlated mediator in the cognitive group (Fig. 2 and Extended Data Fig. 7), highlighting that processes within nerve tissue may underlie this group. These observations suggested that inflammation might arise from mucosal tissues and that additional mechanisms may contribute to pathophysiology underlying the GI and cognitive groups.

**Fig. 3:**
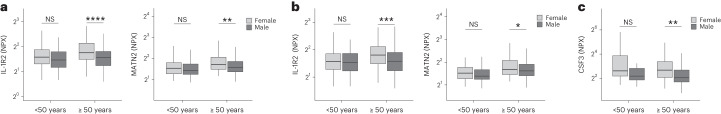
Elevated immune mediator levels are most pronounced in older women with LC. **a**–**c**, Olink-measured plasma protein levels (NPX) of IL-1R2 and MATN2 (**a** and **b**) and CSF3 (**c**) between LC men and LC women divided by age (<50 or ≥50 years) in the cardiorespiratory group (<50 years *n* = 8 and ≥50 years *n* = 270) (**a**), fatigue group (<50 years *n* = 81 and ≥50 years *n* = 227) (**b**) and GI group (<50 years *n* = 34 and ≥50 years *n* = 82) (**c**). the median values were compared between men and women using two-sided Wilcoxon signed-rank test, *P < 0.05, **P < 0.01, ***P < 0.001 and ****P < 0.0001. The box plot center line represents the median, the boundaries represent IQR and the whisker length represents 1.5× IQR.

**Extended Data Fig. 8:**
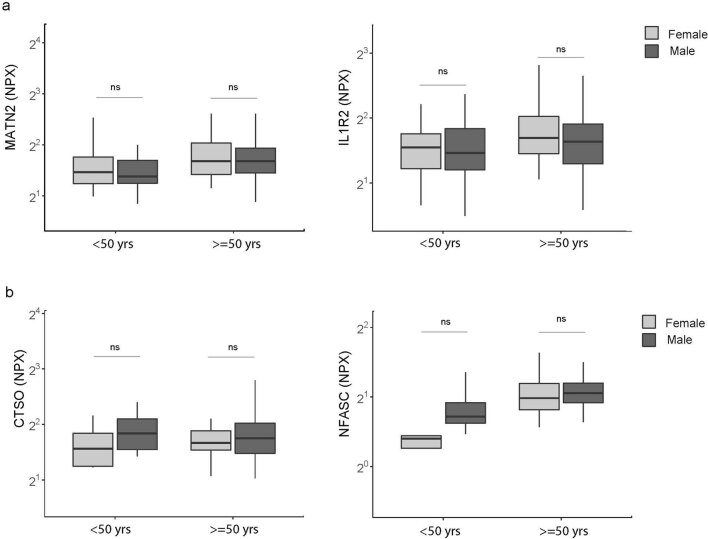
Inflammation in men and women with long COVID. Olink measured plasma protein levels (NPX) between men and women with symptoms, divided by age (<50 or >=50years): **(a)** shows IL1R2 and MATN2 in the Anxiety/Depression group (<50 n = 55, >=50 n = 133), **(b)** shows CTSO and NFASC in the Cognitive group (<50 n = 11, >=50 n = 50). Median values were compared between men and women using two-sided Wilcoxon signed-rank test. Box plot centre line represent the Median and boundaries represent interquartile range (IQR), the whisker length represents 1.5xIQR.

Women were more likely to experience LC (Table 1), as found in previous studies^1^. As estrogen can influence immunological responses^39^, we investigated whether hormonal differences between men and women with LC in our cohort explained this trend. We grouped men and women with LC symptoms into two age groups (those younger than 50 years and those 50 years and older, using age as a proxy for menopause status in women) and compared mediator levels between men and women in each age group, prioritizing those identified by PLR to be higher in LC compared with recovered. As we aimed to understand whether women with LC had stronger inflammatory responses than men with LC, we did not assess differences in men and women in the recovered group. IL-1R2 and MATN2 were significantly higher in women ≥50 years than men ≥50 years in the cardioresp group (Fig. 3a, IL-1R2 and MATN2) and the fatigue group (Fig. 3b). In the GI group, CSF3 was higher in women ≥50 years compared with men ≥50 years (Fig. 3c), indicating that the inflammatory markers observed in women were not likely to be estrogen-dependent. Women have been reported to have stronger innate immune responses to infection and to be at greater risk of autoimmunity^39^, possibly explaining why some women in the ≥50 years group had higher inflammatory proteins than men the same group. Proteins associated with the anxiety/depression (IL-1R2 *P* = 0.11 and MATN2 *P* = 0.61, Extended Data Fig. 8a) and cognitive groups (CTSO *P* = 0.64 and NFASC *P* = 0.41, Extended Data Fig. 8b) were not different between men and women in either age group, consistent with the absent/weak association between sex and these outcomes identified by PLR (Fig. 1e,g). Though our findings suggested that nonhormonal differences in inflammatory responses may explain why some women are more likely to have LC, they require confirmation in adequately powered studies.

**Fig. 4:**
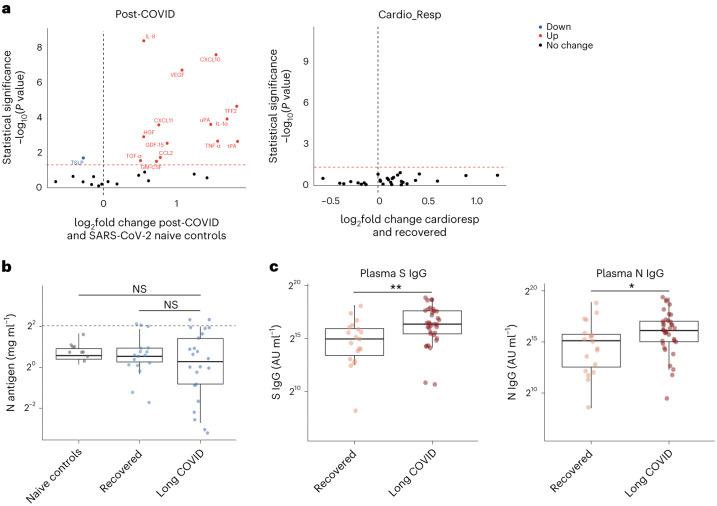
Pronounced mucosal inflammation after COVID-19 is not associated with LC. **a**, Nasal cytokines measured by immunoassay in post-COVID participants (*n* = 64) compared with healthy SARS-CoV-2 naive controls (*n* = 25), and between the the cardioresp group (*n* = 29) and the recovered group (*n* = 31). The red values indicate significantly increased cytokine levels after FDR adjustment (*P* < 0.05) using two-tailed Wilcoxon signed-rank test. **b**, SARS-CoV-2 N antigen measured in sputum by electrochemiluminescence from recovered (*n* = 17) and pooled LC (*n* = 23) groups, compared with BALF from SARS-CoV-2 naive controls (*n* = 9). The horizontal dashed line indicates the lower limit of detection of the assay. **c**, Plasma S- and N-specific IgG responses measured by electrochemiluminescence in the LC (*n* = 35) and recovered (*n* = 19) groups. The median values were compared using two-sided Wilcoxon signed-rank tests, NS *P* > 0.05, *P < 0.05, **P < 0.01, ***P < 0.001 and ****P < 0.0001. The box plot center lines represent the median, the boundaries represent IQR and the whisker length represents 1.5× IQR.

**Extended Data Fig. 9:**
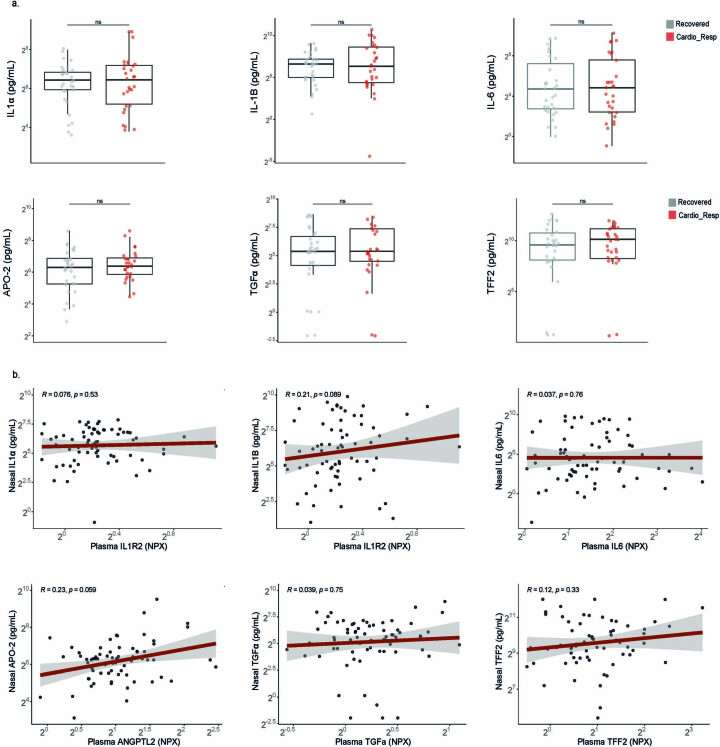
Inflammation in the upper respiratory tract. Nasal cytokines measured by immunoassay in the CardioResp Group (n = 29) and Recovered (n = 31): (**a**) shows IL1a, IL1b, IL-6, APO-2, TGFa, TFF2. Median differences were compared using two-sided Wilcoxon signed-rank test. Box plot centre line represents the Median and boundaries of the box represent interquartile range (IQR), the whisker length represent 1.5xIQR. (**b**) Shows cytokines measured by immunoassay in paired plasma and nasal (n = 70). Correlations between IL1a, IL1b, IL-6, APO-2, TGFa and TFF2 in nasal and plasma samples were compared using Spearman’s rank correlation coefficient (*R*). Shaded areas indicated the 95% confidence interval of R.

To test whether local respiratory tract inflammation persisted after COVID-19, we compared nasosorption samples from 89 participants (recovered, *n* = 31; LC, *n* = 33; and healthy SARS-CoV-2 naive controls, *n* = 25, Supplementary Tables 4 and 5). Several inflammatory markers were elevated in the upper respiratory tract post COVID (including IL-1α, CXCL10, CXCL11, TNF, VEGF and TFF2) when compared with naive controls, but similar between recovered and LC (Fig. 4a). In the cardioresp group (*n* = 29), inflammatory mediators elevated in plasma (for example, IL-6, APO-2, TGF-α and TFF2) were not elevated in the upper respiratory tract (Extended Data Fig. 9a) and there was no correlation between plasma and nasal mediator levels (Extended Data Fig. 9b). This exploratory analysis suggested upper respiratory tract inflammation post COVID was not specifically associated with cardiorespiratory symptoms.

To explore whether SARS-CoV-2 persistence might explain the inflammatory profiles observed in the cardioresp group, we measured SARS-CoV-2 nucleocapsid (N) antigen in sputum from 40 participants (recovered *n* = 17 and LC *n* = 23) collected approximately 6 months post hospitalization (Supplementary Table 6). All samples were compared with prepandemic bronchoalveolar lavage fluid (*n* = 9, Supplementary Table 4). Only four samples (recovered *n* = 2 and LC *n* = 2) had N antigen above the assay’s lower limit of detection, and there was no difference in N antigen concentrations between LC and recovered (Fig. 4b, *P* = 0.78). These observations did not exclude viral persistence, which might require tissues samples for detection^40,41^. On the basis of the hypothesis that persistent viral antigen might prevent a decline in antibody levels over time, we examined the titers of SARS-CoV-2-specific antibodies in unvaccinated individuals (recovered *n* = 19 and LC *n* = 35). SARS-CoV-2 N-specific (*P* = 0.023) and spike (S)-specific (*P* = 0.0040) immunoglobulin G (IgG) levels were elevated in LC compared with recovered (Fig. 4c).

**Extended Data Fig. 10:**
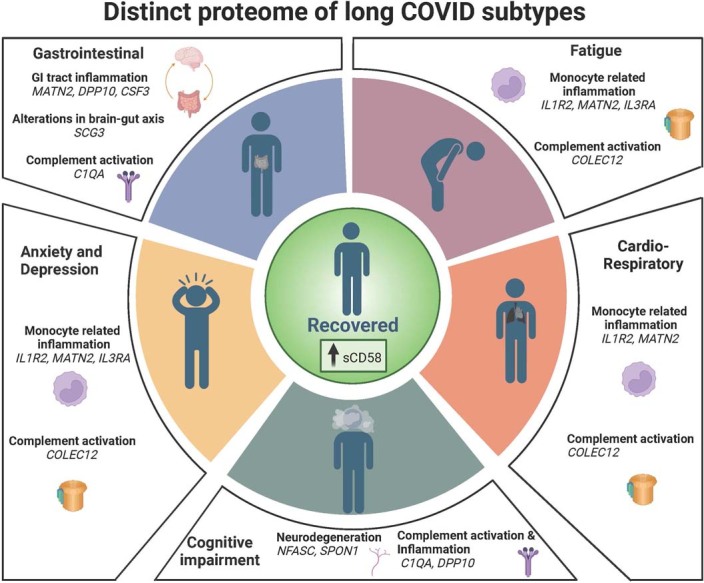
Graphical abstract. Summary of interpretation of key findings from Olink measured proteins and their association with CardioResp (n = 398), Fatigue (n = 342), Anx/Dep (n = 219), GI (n = 134), and Cognitive (n = 65) groups relative to Recovered (n = 233).

Overall, we identified myeloid inflammation and complement activation in the cardioresp, fatigue, anxiety/depression, cognitive and GI groups 6 months after hospitalization (Extended Data Fig. 10). Our findings build on results of smaller studies^5,6,42^ and are consistent with a genome-wide association study that identified an independent association between LC and *FOXP4*, which modulates neutrophilic inflammation and immune cell function^43,44^. In addition, we identified tissue-specific inflammatory elements, indicating that myeloid disturbance in different tissues may result in distinct symptoms. Multiple mechanisms for LC have been suggested, including autoimmunity, thrombosis, vascular dysfunction, SARS-CoV-2 persistence and latent virus reactivation^1^. All these processes involve myeloid inflammation and complement activation^45^. Complement activation in LC has been suggested in a proteomic study in 97 mostly nonhospitalized COVID-19 cases^42^ and a study of 48 LC patients, of which one-third experienced severe acute disease^46^. As components of the complement system are known to have a short half-life^47^, ongoing complement activation suggests active inflammation rather than past tissue damage from acute infection.

Despite the heterogeneity of LC and the likelihood of coexisting or multiple etiologies, our work suggests some common pathways that might be targeted therapeutically and supports the rationale for several drugs currently under trial. Our finding of increased sCD58 levels (associated with suppression of monocyte–lymphocyte interactions^26^) in the recovered group, strengthens our conclusion that myeloid inflammation is central to the biology of LC and that trials of steroids, IL-1 antagonists, JAK inhibitors, naltrexone and colchicine are justified. Although anticoagulants such as apixaban might prevent thrombosis downstream of complement dysregulation, they can also increase the risk of serious bleeding when given after COVID-19 hospitalization^48^. Thus, clinical trials, already underway, need to carefully assess the risks and benefits of anticoagulants (Supplementary Table 2).

Our finding of elevated S- and N-specific IgG in LC could suggest viral persistence, as found in other studies^6,42,49^. Our network analysis indicated that inflammatory proteins in the cardioresp group interacted strongly with ISM1 and ROBO1, which are expressed during respiratory tract infection and regulate lung inflammation^50,51^. Although we were unable to find SARS-CoV-2 antigen in sputum from our LC cases, we did not test for viral persistence in GI tract and lung tissue^40,41^ or in plasma^52^. Evidence of SARS-CoV-2 persistence would justify trials of antiviral drugs (singly or in combination) in LC. It is also possible that autoimmune processes could result in an innate inflammatory profile in LC. Autoreactive B cells have been identified in LC patients with higher SARS-CoV-2-specific antibody titers in a study of mostly mild acute COVID cases (59% WHO 2–3)^42^, a different population from our study of hospitalized cases.

Our observations of distinct protein profiles in GI and cognitive groups support previous reports on distinct associations between Epstein–Barr virus reactivation and neurological symptoms, or autoantibodies and GI symptoms relative to other forms of LC^49,53^. We did not assess autoantibody induction but found evidence of brain–gut axis disturbance (SCG3) in the GI group, which occurs in many autoimmune diseases^54^. We found signatures suggestive of neuroinflammation (C1QA) in the cognitive group, consistent with findings of brain abnormalities on magnetic resonance imaging after COVID-19 hospitalization^55^, as well as findings of microglial activation in mice after COVID-19 (ref. ^56^). Proinflammatory signatures dominated in the cardioresp, fatigue and anxiety/depression groups and were consistent with those seen in non-COVID depression, suggesting shared mechanisms^57^. The association between markers of myeloid inflammation, including IL-3RA, and symptoms was greatest for fatigue. Whilst membrane-bound IL-3RA facilitates IL-3 signaling upstream of myelopoesis^36^ its soluble form (measured in plasma) can bind IL-3 and can act as a decoy receptor, preventing monocyte maturation and enhancing immunopathology^58^. Monocytes from individuals with post-COVID fatigue are reported to have abnormal expression profiles (including reduced CXCR2), suggestive of altered maturation and migration^5,59^. Lung-specific inflammation was suggested by the association between PCDH1 (an airway epithelial adhesion molecule^35^) and cardioresp symptoms.

Our observations do not align with all published observations on LC. One proteomic study of 55 LC cases after generally mild (WHO 2–3) acute disease found that TNF and IFN signatures were elevated in LC^3^. Vasculoproliferative processes and metabolic disturbance have been reported in LC^4,60^, but these studies used uninfected healthy individuals for comparison and cannot distinguish between LC-specific phenomena and residual post-COVID inflammation. A study of 63 adults (LC, *n* = 50 and recovered, *n* = 13) reported no association between immune cell activation and LC 3 months after infection^61^, though myeloid inflammation was not directly measured, and 3 months post infection may be too early to detect subtle differences between LC and recovered cases due to residual acute inflammation.

Our study has limitations. We designed the study to identify inflammatory markers identifying pathways underlying LC subgroups rather than diagnostic biomarkers. The ORs we report are small, but associations were consistent across alternative methods of analysis and when using different LC definitions. Small effect sizes can be expected when using PLR, which shrinks correlated mediator coefficients to reflect combined effects and prevent colinear inflation^62^, and could also result from measurement of plasma mediators that may underestimate tissue inflammation. Although our LC cohort is large compared with most other published studies, some of our subgroups are small (only 60 cases were designated cognitive). Though the performance of the cognitive PLR model was adequate, our findings should be validated in larger studies. It should be noted that our cohort of hospitalized cases may not represent all types of LC, especially those occurring after mild infection. We looked for an effect of acute disease severity within our study and did not find it, and are reassured that the inflammatory profiles we observed were consistent with those seen in smaller studies including nonhospitalized cases^42,46^. Studies of posthospital LC may be confounded by ‘posthospital syndrome’, which encompasses general and nonspecific effects of hospitalization (particularly intensive care)^63^.

In conclusion, we found markers of myeloid inflammation and complement activation in our large prospective posthospital cohort of patients with LC, in addition to distinct inflammatory patterns in patients with cognitive impairment or gastrointestinal symptoms. These findings show the need to consider subphenotypes in managing patients with LC and support the use of antiviral or immunomodulatory agents in controlled therapeutic trials.

## Methods

### Study design and ethics

After hospitalization for COVID-19, adults who had no comorbidity resulting in a prognosis of less than 6 months were recruited to the PHOSP-COVID study (*n* = 719). Patients hospitalized between February 2020 and January 2021 were recruited. Both sexes were recruited and gender was self-reported (female, *n* = 257 and male, *n* = 462). Written informed consent was obtained from all patients. Ethical approvals for the PHOSP-COVID study were given by Leeds West Research Ethics Committee (20/YH/0225).

Symptom data and samples were prospectively collected from individuals approximately 6 months (IQR 5.1–6.8 months and range 3.0–8.3 months) post hospitalization (Fig. 1a), via the PHOSP-COVID multicenter United Kingdom study^64^. Data relating to patient demographics and acute admission were collected via the International Severe Acute Respiratory and Emerging Infection Consortium World Health Organization Clinical Characterisation Protocol United Kingdom (ISARIC4C study; IRAS260007/IRAS126600) (ref. ^65^). Adults hospitalized during the SARS-CoV-2 pandemic were systematically recruited into ISARIC4C. Written informed consent was obtained from all patients. Ethical approval was given by the South Central–Oxford C Research Ethics Committee in England (reference 13:/SC/0149), Scotland A Research Ethics Committee (20/SS/0028) and WHO Ethics Review Committee (RPC571 and RPC572l, 25 April 2013).

Data were collected to account for variables affecting symptom outcome, via hospital records and self-reporting. Acute disease severity was classified according to the WHO clinical progression score: WHO class 3–4: no oxygen therapy; class 5: oxygen therapy; class 6: noninvasive ventilation or high-flow nasal oxygen; and class 7–9: managed in critical care^9^. Clinical data were used to place patients into six categories: ‘recovered’, ‘GI’, ‘cardiorespiratory’, ‘fatigue’, ‘cognitive impairment’ and ‘anxiety/depression’ (Supplementary Table 7). Patient-reported symptoms and validated clinical scores were used when feasible, including Medical Research Council (MRC) breathlessness score, dyspnea-12 score, Functional Assessment of Chronic Illness Therapy (FACIT) score, Patient Health Questionnaire (PHQ)-9 and Generalized Anxiety Disorder (GAD)-7. Cognitive impairment was defined as a Montreal Cognitive Assessment score <26. GI symptoms were defined as answering ‘Yes’ to the presence of at least two of the listed symptoms. ‘Recovered’ was defined by self-reporting. Patients were placed in multiple groups if they experienced a combination of symptoms.

Matched nasal fluid and sputum samples were prospectively collected from a subgroup of convalescent patients approximately 6 months after hospitalization via the PHOSP-COVID study. Nasal and bronchoalveolar lavage fluid (BALF) collected from healthy volunteers before the COVID-19 pandemic were used as controls (Supplementary Table 4). Written consent was obtained for all individuals and ethical approvals were given by London–Harrow Research Ethics Committee (13/LO/1899) for the collection of nasal samples and the Health Research Authority London–Fulham Research Ethics Committee (IRAS project ID 154109; references 14/LO/1023, 10/H0711/94 and 11/LO/1826) for BALF samples.

### Procedures

Ethylenediaminetetraacetic acid plasma was collected from whole blood taken by venepuncture and frozen at −80 °C as previously described^7,66^. Nasal fluid was collected using a NasosorptionTM FX·I device (Hunt Developments), which uses a synthetic absorptive matrix to collect concentrated nasal fluid. Samples were eluted and stored as previously described^67^. Sputum samples were collected via passive expectoration and frozen at −80 °C without the addition of buffers. Sputum samples from convalescent individuals were compared with BALF from healthy SARS-CoV-2-naive controls, collected before the pandemic. BALF samples were used to act as a comparison for lower respiratory tract samples since passively expectorated sputum from healthy SARS-CoV-2-naive individuals was not available. BALF samples were obtained by instillation and recovery of up to 240 ml of normal saline via a fiberoptic bronchoscope. BALF was filtered through 100 µM strainers into sterile 50 ml Falcon tubes, then centrifuged for 10 min at 400 *g* at 4 °C. The resulting supernatant was transferred into sterile 50 ml Falcon tubes and frozen at −80 °C until use. The full methods for BALF collection and processing have been described previously^68,69^.

### Immunoassays

To determine inflammatory signatures that associated with symptom outcomes, plasma samples were analyzed on an Olink Explore 384 Inflammation panel^70^. Supplementary Table 8 (Appendix 1) lists all the analytes measured. To ensure the validity of results, samples were run in a single batch with the use of negative controls, plate controls in triplicate and repeated measurement of patient samples between plates in duplicate. Samples were randomized between plates according to site and sample collection date. Randomization between plates was blind to LC/recovered outcome. Data were first normalized to an internal extension control that was included in each sample well. Plates were standardized by normalizing to interplate controls, run in triplicate on each plate. Each plate contained a minimum of four patient samples, which were duplicates on another plate; these duplicate pairs allowed any plate to be linked to any other through the duplicates. Data were then intensity normalized across all cohort samples. Finally, Olink results underwent quality control processing and samples or analytes that did not reach quality control standards were excluded. Final normalized relative protein quantities were reported as log_2_ NPX values.

To further validate our findings, we performed conventional electrochemiluminescence (ECL) assays and enzyme-linked immunosorbent assay for Olink mediators that were associated with symptom outcome (Supplementary Methods). Contemporaneously collected plasma samples were available from 58 individuals. Like most omics platforms, Olink measures relative quantities, so perfect agreement with conventional assays that measure absolute concentrations is not expected.

Sputum samples were thawed before analysis and sputum plugs were extracted with the addition of 0.1% dithiothreitol creating a one in two sample dilution, as previously described^71^. SARS-CoV-2 S and N proteins were measured by ECL S-plex assay at a fixed dilution of one in two (Mesoscale Diagnostics), as per the manufacturers protocol^72^. Control BALF samples were thawed and measured on the same plate, neat. The S-plex assay is highly sensitive in detecting viral antigen in respiratory tract samples^73^.

Nasal cytokines were measured by ECL (mesoscale discovery) and Luminex bead multiplex assays (Biotechne). The full methods and list of analytes are detailed in Supplementary Methods.

### Statistics and reproducibility

Clinical data was collected via the PHOSP REDCap database, to which access is available under reasonable request as per the data sharing statement in the manuscript. All analyses were performed within the Outbreak Data Analysis Platform (ODAP). All data and code can be accessed using information in the ‘Data sharing’ and ‘Code sharing’ statements at the end of the manuscript. No statistical method was used to predetermine sample size. Data distribution was assumed to be normal but this was not formally tested. Olink assays and immunoassays were randomized and investigators were blinded to outcomes.

To determine protein signatures that associated with each symptom outcome, a ridge PLR was used. PLR shrinks coefficients to account for combined effects within high-dimensional data, preventing false discovery while managing multicollinearity^34^. Thus, PLR was chosen a priori as the most appropriate model to assess associations between a large number of explanatory variables (that may work together to mediate effects) and symptom outcome^34,62,70,74^. In keeping with our aim to perform an unbiased exploration of inflammatory process, the model alpha was set to zero, facilitating regularization without complete penalization of any mediator. This enabled review of all possible mediators that might associate with LC^62^.

A 50 repeats tenfold nested cross-validation was used to select the optimal lambda for each model and assess its accuracy (Extended Data Fig. 1). The performance of the cognitive impairment model was influenced by the imbalance in size of the symptom group (*n* = 60) relative to recovered (*n* = 250). The model was weighted to account for this imbalance resulting in a sensitivity of 0.98, indicating its validity. We have expanded on the model performance and validation approaches in Supplementary Information.

Age, sex, acute disease severity and preexisting comorbidities were included as covariates in the PLR analysis (Supplementary Tables 1 and 3). Covariates were selected a priori using features reported to influence the risk of LC and inflammatory responses^1,39,64,75^. Ethnicity was not included since it has been shown not to predict symptom outcome in this cohort^64^. Individuals with missing data were excluded from the regression analysis. Each symptom group was compared with the ‘recovered’ group. The model coefficients of each covariate were converted into ORs for each outcome and visualized in a forest plot, after removing variables associated with regularized OR between 0.98 and 1.02 or in cases where most variables fell outside of this range, using mediators associated with the highest decile of coefficients either side of this range. This enabled exclusion of mediators with effect sizes that were unlikely to have clinical or mechanistic importance since the ridge PLR shrinks and orders coefficients according to their relative importance rather than making estimates with standard error. Thus, confidence intervals cannot be appropriately derived from PLR, and forest plot error bars were calculated using the median accuracy of the model generated by the nested cross-validation. To verify observations made through PLR analysis, we also performed an unadjusted PLR, an unadjusted logistic regression and a PLS analysis. Univariate analyses using Wilcoxon signed-rank test was also performed (Supplementary Table 8, Appendix 1). Analyses were performed in R version 4.2.0 using ‘data.table v1.14.2’, ‘EnvStats v2.7.0’ ‘tidyverse v1.3.2’, ‘lme4 v1.1-32’, ‘caret v6.0-93’, ‘glmnet v4.1-6’, ‘mdatools v0.14.0’, ‘ggpubbr v0.4.0’ and ‘ggplot2 v3.3.6’ packages.

To further investigate the relationship between proteins elevated in each symptom group, we performed a correlation network analysis using Spearman’s rank correlation coefficient and false discovery rate (FDR) thresholding. The mediators visualized in the PLR forest plots, which were associated with cardiorespiratory symptoms, fatigue, anxiety/depression GI symptoms and cognitive impairment were used, respectively. Analyses were performed in R version 4.2.0 using ‘bootnet v1.5.6*’* and ‘qgraph v1.9.8*’* packages.

To determine whether differences in protein levels between men and women related to hormonal differences, we divided each symptom group into premenopausal and postmenopausal groups using an age cutoff of 50 years old. Differences between sexes in each group were determined using the Wilcoxon signed-rank test. To understand whether antigen persistence contributed to inflammation in adults with LC, the median viral antigen concentration from sputum/BALF samples and cytokine concentrations from nasal samples were compared using the Wilcoxon signed-rank test. All tests were two-tailed and statistical significance was defined as a *P* value < 0.05 after adjustment for FDR (*q*-value of 0.05). Analyses were performed in R version 4.2.0 using ‘bootnet v1.5.6’ and ‘qgraph v1.9.8’ packages.

Extended Data Fig. 10 was made using Biorender, accessed at www.biorender.com.

### Reporting summary

Further information on research design is available in the Nature Portfolio Reporting Summary linked to this article.

## Online content

Any methods, additional references, Nature Portfolio reporting summaries, source data, extended data, supplementary information, acknowledgements, peer review information; details of author contributions and competing interests; and statements of data and code availability are available at 10.1038/s41590-024-01778-0.

### Supplementary information

Supplementary Information Supplementary Methods, Statistics and reproducibility statement, Supplementary Results, Supplementary Tables 1–7, Extended data figure legends, Appendix 1 (Supplementary Table 8), Appendix 2 (PHOSP-COVID author list) and Appendix 3 (ISARIC4C author list).

Reporting Summary

## Data Availability

This is an open access article under the CC BY 4.0 license. The PHOSP-COVID protocol, consent form, definition and derivation of clinical characteristics and outcomes, training materials, regulatory documents, information about requests for data access, and other relevant study materials are available online at ref. ^76^. Access to these materials can be granted by contacting phosp@leicester.ac.uk and Phospcontracts@leicester.ac.uk. The ISARIC4C protocol, data sharing and publication policy are available at https://isaric4c.net. ISARIC4C’s Independent Data and Material Access Committee welcomes applications for access to data and materials (https://isaric4c.net). The datasets used in the study contain extensive clinical information at an individual level that prevent them from being deposited in an public depository due to data protection policies of the study. Study data can only be accessed via the ODAP, a protected research environment. All data used in this study are available within ODAP and accessible under reasonable request. Data access criteria and information about how to request access is available online at ref. ^76^. If criteria are met and a request is made, access can be gained by signing the eDRIS user agreement.

## Code availability

Code was written within the ODAP, using R v4.2.0 and publicly available packages (‘data.table v1.14.2’, ‘EnvStats v2.7.0’, ‘tidyverse v1.3.2’, ‘lme4 v1.1-32’, ‘caret v6.0-93’, ‘glmnet v4.1-6’, ‘mdatools v0.14.0’, ‘ggpubbr v0.4.0’, ‘ggplot2 v3.3.6’, ‘bootnet v1.5.6’ and ‘qgraph v1.9.8’ packages). No new algorithms or functions were created and code used in-built functions in listed packages available on CRAN. The code used to generate data and to analyze data is publicly available at https://github.com/isaric4c/wiki/wiki/ISARIC; https://github.com/SurgicalInformatics/cocin_cc and https://github.com/ClaudiaEfstath/PHOSP_Olink_NatImm.

## References

[1] Davis HE, McCorkell L, Vogel JM, Topol EJ (2023). Long COVID: major findings, mechanisms and recommendations. Nat. Rev. Microbiol..

[2] Antonelli M, Pujol JC, Spector TD, Ourselin S, Steves CJ (2022). Risk of long COVID associated with delta versus omicron variants of SARS-CoV-2. Lancet.

[3] Talla A (2023). Persistent serum protein signatures define an inflammatory subcategory of long COVID. Nat. Commun..

[4] Captur G (2022). Plasma proteomic signature predicts who will get persistent symptoms following SARS-CoV-2 infection. EBioMedicine.

[5] Scott, N. A. et al. Monocyte migration profiles define disease severity in acute COVID-19 and unique features of long COVID. *Eur. Respir. J.*10.1183/13993003.02226-2022 (2023). 10.1183/13993003.02226-2022 PMC10040898 36922030

[6] Klein J (2023). Distinguishing features of Long COVID identified through immune profiling. Nature.

[7] Evans RA (2022). Clinical characteristics with inflammation profiling of long COVID and association with 1-year recovery following hospitalisation in the UK: a prospective observational study. Lancet Respir. Med.

[8] Houchen-Wolloff L (2022). Joint patient and clinician priority setting to identify 10 key research questions regarding the long-term sequelae of COVID-19. Thorax.

[9] Marshall JC (2020). A minimal common outcome measure set for COVID-19 clinical research. Lancet Infect. Dis..

[10] Post COVID-19 condition (long COVID). *World Health Organization* https://www.who.int/europe/news-room/fact-sheets/item/post-covid-19-condition#:~:text=Definition,months%20with%20no%20other%20explanation (2022).

[11] Peters VA, Joesting JJ, Freund GG (2013). IL-1 receptor 2 (IL-1R2) and its role in immune regulation. Brain Behav. Immun..

[12] Luo Z (2022). Monocytes augment inflammatory responses in human aortic valve interstitial cells via β2-integrin/ICAM-1-mediated signaling. Inflamm. Res..

[13] Bendall LJ, Bradstock KF (2014). G-CSF: from granulopoietic stimulant to bone marrow stem cell mobilizing agent. Cytokine Growth Factor Rev..

[14] Ma YJ (2015). Soluble collectin-12 (CL-12) is a pattern recognition molecule initiating complement activation via the alternative pathway. J. Immunol..

[15] Laursen NS (2020). Functional and structural characterization of a potent C1q inhibitor targeting the classical pathway of the complement system. Front. Immunol..

[16] Dejanovic B (2022). Complement C1q-dependent excitatory and inhibitory synapse elimination by astrocytes and microglia in Alzheimer’s disease mouse models. Nat. Aging.

[17] Xue G, Hua L, Zhou N, Li J (2021). Characteristics of immune cell infiltration and associated diagnostic biomarkers in ulcerative colitis: results from bioinformatics analysis. Bioengineered.

[18] He T (2022). Integrative computational approach identifies immune‐relevant biomarkers in ulcerative colitis. FEBS Open Bio..

[19] Sundin J (2018). Fecal chromogranins and secretogranins are linked to the fecal and mucosal intestinal bacterial composition of IBS patients and healthy subjects. Sci. Rep..

[20] Kriebel M, Wuchter J, Trinks S, Volkmer H (2012). Neurofascin: a switch between neuronal plasticity and stability. Int. J. Biochem. Cell Biol..

[21] Woo W-M (2008). The *C. elegans* F-spondin family protein SPON-1 maintains cell adhesion in neural and non-neural tissues. Development.

[22] Yadati T, Houben T, Bitorina A, Shiri-Sverdlov R (2020). The ins and outs of cathepsins: physiological function and role in disease management. Cells.

[23] Taquet M (2023). Acute blood biomarker profiles predict cognitive deficits 6 and 12 months after COVID-19 hospitalization. Nat. Med..

[24] Siggins MK (2023). Alternative pathway dysregulation in tissues drives sustained complement activation and predicts outcome across the disease course in COVID‐19. Immunology.

[25] Pol JG, Caudana P, Paillet J, Piaggio E, Kroemer G (2020). Effects of interleukin-2 in immunostimulation and immunosuppression. J. Exp. Med..

[26] Zhang Y, Liu Q, Yang S, Liao Q (2021). CD58 immunobiology at a glance. Front. Immunol..

[27] Demydchuk M (2017). Insights into Hunter syndrome from the structure of iduronate-2-sulfatase. Nat. Commun..

[28] Wang Z (2020). DNER promotes epithelial–mesenchymal transition and prevents chemosensitivity through the Wnt/β-catenin pathway in breast cancer. Cell Death Dis..

[29] Bonilla H (2023). Therapeutic trials for long COVID-19: a call to action from the interventions taskforce of the RECOVER initiative. Front. Immunol..

[30] Wik L (2021). Proximity extension assay in combination with next-generation sequencing for high-throughput proteome-wide analysis. Mol. Cell. Proteomics.

[31] Measuring protein biomarkers with Olink—technical comparisons and orthogonal validation. *Olink Proteomics* https://www.olink.com/content/uploads/2021/09/olink-technical-comparisons-and-orthogonal-validation-1118-v2.0.pdf (2021).

[32] COVID-19 rapid guideline: managing the long-term effects of COVID-19. *National Institute for Health and Care Excellence (NICE), Scottish Intercollegiate Guidelines Network (SIGN) and Royal College of General Practitioners (RCGP)* https://www.nice.org.uk/guidance/ng188/resources/covid19-rapid-guideline-managing-the-longterm-effects-of-covid19-pdf-51035515742 (2022).

[33] Long COVID or post-COVID conditions. *Centers for Disease Control and Prevention* https://www.cdc.gov/coronavirus/2019-ncov/long-term-effects/index.html#:~:text=Long%20COVID%20is%20broadly%20defined,after%20acute%20COVID%2D19%20infection (2023).

[34] Firinguetti L, Kibria G, Araya R (2017). Study of partial least squares and ridge regression methods. Commun. Stat. Simul. Comput.

[35] Mortensen LJ, Kreiner-Moller E, Hakonarson H, Bonnelykke K, Bisgaard H (2014). The PCDH1 gene and asthma in early childhood. Eur. Respir. J..

[36] Tong Y (2020). The RNFT2/IL-3Rα axis regulates IL-3 signaling and innate immunity. JCI Insight.

[37] Wu Y (2021). Effect of ISM1 on the immune microenvironment and epithelial-mesenchymal transition in colorectal cancer. Front. Cell Dev. Biol..

[38] Luo GG, Ou JJ (2015). Oncogenic viruses and cancer. Virol. Sin..

[39] Klein SL, Flanagan KL (2016). Sex differences in immune responses. Nat. Rev. Immunol..

[40] Gaebler C (2021). Evolution of antibody immunity to SARS-CoV-2. Nature.

[41] Bussani R (2023). Persistent SARS‐CoV‐2 infection in patients seemingly recovered from COVID‐19. J. Pathol..

[42] Woodruff MC (2023). Chronic inflammation, neutrophil activity, and autoreactivity splits long COVID. Nat. Commun..

[43] Lammi, V. et al. Genome-wide association study of long COVID. Preprint at *medRxiv*10.1101/2023.06.29.23292056 (2023).

[44] Ismailova A (2023). Identification of a forkhead box protein transcriptional network induced in human neutrophils in response to inflammatory stimuli. Front. Immunol..

[45] Beurskens FJ, van Schaarenburg RA, Trouw LA (2015). C1q, antibodies and anti-C1q autoantibodies. Mol. Immunol..

[46] Cervia-Hasler C (2024). Persistent complement dysregulation with signs of thromboinflammation in active long Covid. Science.

[47] Morgan BP, Harris CL (2015). Complement, a target for therapy in inflammatory and degenerative diseases. Nat. Rev. Drug Discov..

[48] Toshner, M. R. et al. Apixaban following discharge in hospitalised adults with COVID-19: preliminary results from a multicentre, open-label, randomised controlled platform clinical trial. Preprint at *medRxiv*, 10.1101/2022.12.07.22283175 (2022).

[49] Su Y (2022). Multiple early factors anticipate post-acute COVID-19 sequelae. Cell.

[50] Branchfield K (2016). Pulmonary neuroendocrine cells function as airway sensors to control lung immune response. Science.

[51] Rivera-Torruco G (2019). Isthmin 1 identifies a subset of lung hematopoietic stem cells and it is associated with systemic inflammation. J. Immunol..

[52] Swank Z (2023). Persistent circulating severe acute respiratory syndrome coronavirus 2 spike is associated with post-acute coronavirus disease 2019 sequelae. Clin. Infect. Dis..

[53] Peluso MJ (2023). Chronic viral coinfections differentially affect the likelihood of developing long COVID. J. Clin. Invest..

[54] Bellocchi C (2022). The interplay between autonomic nervous system and inflammation across systemic autoimmune diseases. Int. J. Mol. Sci..

[55] Raman B (2023). Multiorgan MRI findings after hospitalisation with COVID-19 in the UK (C-MORE): a prospective, multicentre, observational cohort study. Lancet Respir. Med.

[56] Fernández-Castañeda A (2022). Mild respiratory COVID can cause multi-lineage neural cell and myelin dysregulation. Cell.

[57] Dantzer R, O’Connor JC, Freund GG, Johnson RW, Kelley KW (2008). From inflammation to sickness and depression: when the immune system subjugates the brain. Nat. Rev. Neurosci..

[58] Broughton SE (2014). Dual mechanism of interleukin-3 receptor blockade by an anti-cancer antibody. Cell Rep..

[59] Ley K, Miller YI, Hedrick CC (2011). Monocyte and macrophage dynamics during atherogenesis. Arterioscler. Thromb. Vasc. Biol..

[60] Iosef C (2023). Plasma proteome of long-COVID patients indicates HIF-mediated vasculo-proliferative disease with impact on brain and heart function. J. Transl. Med..

[61] Santopaolo M (2023). Prolonged T-cell activation and long COVID symptoms independently associate with severe COVID-19 at 3 months. eLife.

[62] Friedman J, Hastie T, Tibshirani R (2010). Regularization paths for generalized linear models via coordinate descent. J. Stat. Softw..

[63] Voiriot G (2022). Chronic critical illness and post-intensive care syndrome: from pathophysiology to clinical challenges. Ann. Intensive Care.

[64] Evans RA (2021). Physical, cognitive, and mental health impacts of COVID-19 after hospitalisation (PHOSP-COVID): a UK multicentre, prospective cohort study. Lancet Respir. Med.

[65] Docherty, A. B. et al. Features of 20,133 UK patients in hospital with covid-19 using the ISARIC WHO clinical characterisation protocol: prospective observational cohort study. *BMJ*10.1136/bmj.m1985 (2020). 10.1136/bmj.m1985 PMC7243036 32444460

[66] Elneima, O. et al. Cohort profile: post-hospitalisation COVID-19 study (PHOSP-COVID). Preprint at *medRxiv*10.1101/2023.05.08.23289442 (2023).

[67] Liew F (2023). SARS-CoV-2-specific nasal IgA wanes 9 months after hospitalisation with COVID-19 and is not induced by subsequent vaccination. EBioMedicine.

[68] Ascough S (2022). Divergent age-related humoral correlates of protection against respiratory syncytial virus infection in older and young adults: a pilot, controlled, human infection challenge model. Lancet Healthy Longev..

[69] Guvenel A (2019). Epitope-specific airway-resident CD4+ T cell dynamics during experimental human RSV infection. J. Clin. Invest..

[70] Greenwood CJ (2020). A comparison of penalised regression methods for informing the selection of predictive markers. PLoS ONE.

[71] Higham A (2016). Leukotriene B4 levels in sputum from asthma patients. ERJ Open Res..

[72] SARS-CoV-2 spike kit. *MSD* https://www.mesoscale.com/~/media/files/product%20inserts/s-plex%20sars-cov-2%20spike%20kit%20product%20insert.pdf (2023).

[73] Ren A (2022). Ultrasensitive assay for saliva-based SARS-CoV-2 antigen detection. Clin. Chem. Lab. Med..

[74] Breheny P, Huang J (2009). Penalized methods for bi-level variable selection. Stat. Interface.

[75] Thwaites RS (2021). Inflammatory profiles across the spectrum of disease reveal a distinct role for GM-CSF in severe COVID-19. Sci. Immunol..

[76] Resources. *PHOSP-COVID* https://phosp.org/resource/ (2022).

