# Supplementary material for: Large-scale phenotyping of patients with long COVID post-hospitalization reveals mechanistic subtypes of disease
Source: Nat Immunol. 2024 Apr 8;25(4):607–21. doi: 10.1038/s41590-024-01778-0 (PMC11003868; doi:10.1038/s41590-024-01778-0)
Supplement: Supplementary file 1 — Supplementary Methods, Statistics and reproducibility statement, Supplementary Results, Supplementary Tables 1–7, Extended data figure legends, Appendix 1 (Supplementary Table 8), Appendix 2 (PHOSP-COVID author list) and Appendix 3 (ISARIC4C author list). [file 41590_2024_1778_MOESM1_ESM.pdf]

nature immunology

Letter
https://doi.org/10.1038/s41590-024-01778-0

Large-scale phenotyping of patients with long COVID post-hospitalization reveals mechanistic subtypes of disease

In the format provided by the authors and unedited

Supplementary Materials

Contents

**Supplementary methods**	Pages 1-2
**Statistics and reproducibility**	Page 3
**Supplementary tables and legends**	Pages 4-8
**Supplementary Materials References**	Page 9-10
**Supplementary Table 8**	Appendix 1
**PHOSP COVID Consortium Author list**	Appendix 2
**ISARIC4C Consortium Author list**	Appendix 3

Supplementary methods

Cytokine immunoassay

To validate key results from the Olink analysis, we performed conventional immunoassays on contemporaneously collected plasma samples, available from 58 individuals. We measured G-CSF (CSF3), IL2, TFF2, TNFα and IL1R2 by electrochemiluminescence (ECL, Mesoscale Diagnostics (MSD), Rockville, Maryland, USA). Custom R plex (IL1R2 cat:K151ANVR-2, TFF2 cat: F21ACM-3) and U-plex (G-CSF, IL-2 cat: K15067M-1) MSD kits were used in addition to the Proinflammatory Panel 1 V-plex kit, for measurement of TNFα. Samples were thawed at room temperature and analysed, according to manufacturer's protocol (Available from: https://www.mesoscale.com/). MSD U-plex plates consisted of 96 wells with 2 spots, coated with a capture antibody and linker for G-CSF or IL2 respectively. The R-plex custom plate consisted of 96 wells with 1 spot coated with streptavidin. A solution of biotinylated capture antibody for IL1R2 or TFF2 was incubated on each R-plex plate to enable measurement of the chosen analytes. The V-plex kit consisted of a 96-well plate, precoated with a TNFα capture antibody. Samples were then incubated neat followed by the addition of a labelled detection antibody. MSD plates were measured on a MESO QuickPlex SQ 120 Reader (MSD) and cytokine concentrations were calculated using a reference standard and assigned pg/ml. Samples with values below the limit of detection (LLOD) were excluded from the validation analysis since assays perform poorly at these concentrations. Data were log2 transformed prior to analysis.

ELISA was used to measure IL3RA, (Abcam, Cambridge, United Kingdom, cat: ab300317). Samples were thawed at room temperature and added to the wells neat following the manufacturers' instructions for the kit. ELISA plates were quantified on a FLUOstar Omega (BMG labtech) at 450nm and cytokine concentrations were determined using the reference standard. Values below the limit of detection (LLOD) were excluded from the validation analysis since assays perform poorly at these concentrations. Data were log2 transformed prior to analysis.

Nasal Angiopoeitin-2, CXCL-10, Galectin-9, GM-CSF, IL-1α, IL-33, TSLP, CXCL11, VEGF, CCL2, CXCL11, GDF-15, HGF, Thrombomodulin and IL-15 were measured by custom Luminex discovery bead-based ELISA (Biotechne, Minneapolis, United States, configuration Ubdal728; cat:LXSAHM-15). IFNγ, IL-1β, IL-2, IL-4, IL-6, IL-8, IL-19, IL-12p70, IL-13 and TNFα were measured using the Proinflammatory Panel 1 V-plex kit, which uses an MSD electrochemiluminescence multiplex assay (Mesoscale Diagnostics, Rockville, Maryland, USA, cat K15049D-1). Trefoil factor 2 (TFF2), Tissue Plasminogen Activator (tPA), Follistatin and TGFα were measured using an R-plex custom electrochemiluminescence multiplex assay (MSD; cat: F21ACN-3 & F21ACZ-3).

Nasal samples were thawed at room temperature and analysed at a fixed dilution of 1 in 2 using the R-plex and Luminex assays, and neat using the Proinflammatory Panel 1 V-plex kit. Plates were

prepared and analysed according to the manufacturers protocol[1–3]. MSD V-plex plates consisted of 96 wells with 10 spots, each precoated with a capture antibody for the given analyte, and to which neat sample was added and incubated. The R-plex custom plate consisted of 96 wells with 4 spots coated with streptavidin. A solution of biotinylated capture antibody for each analyte was incubated on each R-plex plate to enable measurement of the chosen analytes. Nasal fluid was then added and incubated, followed by the addition of a labelled detection antibody. MSD plates were measured on a MESO QuickPlex SQ 120 Reader (MSD) and cytokine concentrations were calculated using a reference standard and assigned pg/ml. For the Luminex assay, samples were incubated on a 96 well plate with addition of beads labelled with the analyte capture antibody. Luminex plates were read on a BioPlex200 instrument (Bio-Rad, UK) and cytokine concentrations were derived from the reference standard and assigned pg/ml. All values at or below the LLOD were replaced with LLOD. All values at or above the upper limit of detection (ULOD) were replaced with ULOD.

Viral antigen measurements from sputum

Sputum samples were thawed at room temperature and sputum plugs were extracted and weighed. Eight millilitres of PBS were added per mg of sputum to create a solution and samples were centrifuged at 400g for 10 minutes at 4°C. Four volumes of PBS supernatant were then removed and stored at -80°C. Another 4 volumes were removed for the addition of 4 volumes of 0.1% dithiothreitol (DTT), creating a 1 in 2 dilution, and stored at -80°C. DTT was added to reduce matrix effects and enable measurement of sputum proteins, and this method has been previously validated[4]. Sputum diluted in DTT was used for the measurement of viral Spike (S) and Nucleopcapsid (N) antigens using a S plex SARS-CoV-2 N kit and SARS-CoV-2 S kit (MSD). The kits used ECL to detect antigen concentrations and plates consisted of 96 wells containing 1 spot coated with a streptavidin bound biotinylated capture antibody. Plates were blocked with MSD Blocker A, followed by the addition of sputum samples in DTT, analysed at a fixed dilution of 1 in 2. After sample incubation a TURBO-BOOST detection antibody was added followed by the addition of a TURBO-TAG which enables higher sensitivity of the assay[5]. All values below the lower limit of detection (LLOD) were replaced with LLOD if MSD software was unable to calculate a value.

Statistics and reproducibility

Performance of the adjusted penalised ridge logistic regression

The AUC of the final models were >0.6 for all symptom outcomes and the classification errors were all <0.4 (Extended Data Fig. 9). These metrics indicate that the model assigned symptom groups correctly based on the identified variables more frequently than would be expected by chance (in which case: AUC=< 0.5 and Classification error>=0.5). The cognitive impairment model was weighted due to the imbalance in group size (60 Vs 233), preventing misclassification to the majority class (Recovered), and the sensitivity of the weighted model was 0.98. Since the objective was not a predictive model, the accuracy metrics were considered adequate for extracting associations between symptoms and variables.

The small effect sizes reported in PLR were small, which can be explained by how the model derives coefficients. PLR shrinks correlated mediator coefficients towards each other to account for combined effects and prevent colinear inflation[6]. Thus, the effect sizes do not necessarily diminish the potential mechanistic significance of our findings, as inflammatory proteins are expected to mediate effects in combination.

Validation of results through alternative analytical approaches

PLS and univariate analyses were used to support the reliability of PLR findings, but were not used to draw conclusions. PLR enables relationships between mediators to be accounted for without false discovery[7,8]. In highly correlated data such as ours, PLR has been shown to consistently outperform PLS, which tends to overestimate coefficient variance[9,10]. Thus PLR was used for the primary analysis and to draw conclusions.

Supplementary tables

Cardiorespiratory	Neuropsychiatric	Gastrointestinal
Atrial fibrillation	Migraine	Inflammatory Bowel disease
Ischaemic heart disease	Previous Cerebrovascular Accident (CVA)	Irritable bowel syndrome
Congestive heart failure	Dementia	Peptic ulcer disease
Coronary heart disease	Multiple sclerosis	Chronic liver disease
Valvular heart disease	Depression	Any other chronic gastrointestinal disorder
Chronic Obstructive Pulmonary Disease (COPD)	Anxiety	
Asthma	Previous treatment by mental health professional	
Interstitial lung disease	Any other chronic neurological disorder	
Bronchiectasis		
Previous pulmonary embolus		
Previous Tuberculosis infection		
Lung carcinoma		
Any other chronic cardiac or lung disorder		

Supplementary Table 1. Comorbidities included as covariates in analysis. Details of patient questionnaire items used to assign patient comorbidity status relevant to long COVID symptom outcomes. Relevant comorbidities were included as covariates in the model for each symptom group. All comorbidities recorded by the PHOSP-COVID patient questionnaire were included as covariates for the Fatigue model, including the response ‘Yes’ to variable ‘any other comorbidity?’.

Targeted aetiology	Drug	Mechanisms of action relevant to long COVID inflammatory profiles	Clinical trial identifier
Viral persistence	Nirmatrelvir/ritonavir (Paxlovid)	Anti-SARS-CoV-2	NCT05668091<br>NCT05595369<br>NCT05576662<br>NCT05823896<br>NCT05852873
	Remdesivir	Anti-SARS-CoV-2	NCT04978259<br>NCT05911906
	Favipiravir	Anti-SARS-CoV-2	NCT04448119
	Rintatolimod (Ampligen)	TLR3 agonist and antiviral action through induction of interferons	NCT05592418
Autoimmunity and/or chronic inflammation	Anakinra	IL-1 signalling blockade	NCT05926505
	Infliximab	TNF signalling blockade	NCT05220280
	Imatinib	JAK inhibition and suppression of vascular[11] and macrophage/TNFα dependent[12] inflammation	NCT05220280
	Baricitinib	Anti-SARS-CoV-2 effects<br>Inhibits multiple cytokines including IL-1, IL-6 and G-CSF[13–15]	NCT05858515
	Ibudilast	Phosphodiesterase inhibition and reduction of monocyte derived cytokines	NCT05513560
	Cannabidiol<br>Xltanplus<br>Naltrexone	Agonist to PPARγ and CBD receptors expressed by immune cells to suppress NFkB, TNFα, IL-1 and IL-6 signalling.[16]	NCT04997395<br>NCT05467904<br>NCT04604704<br>NCT05430152<br>NCT05946551
	Methylprednisolone	Broad suppression of immune responses, including vascular inflammation	NCT05986422
	Montelukast	Leukotriene receptor antagonist which can suppress IL-1 and IL-6 in a range of tissues.[17]	NCT04695704
	Colchicine	Inhibits neutrophil derived inflammation and suppresses N-κB inflammatory pathways including IL-1 and TNFα[18]	NCT05648734<br>STIMULATE-ICP<br>/ISRCTN10665760
Complement and/or Thrombosis	Pentoxyfylline	Vasodilation and suppression of vascular inflammation[19]	NCT05513560
	Rivaroxaban	Anticoagulation	STIMULATE-ICP<br>/ISRCTN10665760

Supplementary Table 2. Drugs repurposed for long COVID. Trials listed are those using drugs already licensed in other disease, which have potential to target long COVID aetiologies consistent with observed inflammatory signatures. Trials were identified by searching clinicaltrials.gov, September 2023.

Cardiorespiratory	OR	upper_SE	lower_SE	*p*-value	
Female	2.05	2.46	1.64	0.0003	***
Cardioresp comorbidity	1.45	1.76	1.15	0.05	*
WHO acute severity: class 6-9	1.44	1.71	1.17	0.06	NS
Age	1.00	1.00	0.99	0.59	NS
Fatigue	OR	upper_SE	lower_SE	*p*-value	
Any Comorbidity	2.24	2.71	1.77	0.0001	***
Female	2.19	2.64	1.74	0.0002	***
Age	0.98	0.99	0.98	0.05	NS
WHO acute severity: class 6-9	1.38	1.66	1.11	0.10	NS
Anxiety/depression	OR	upper_SE	lower_SE	*p*-value	
Neuro/Psych comorbidity	4.30	5.46	3.14	6.90E-08	****
Female	2.08	2.56	1.60	0.002	**
WHO acute severity: class 6-9	1.52	1.86	1.18	0.06	NS
Age	0.98	0.99	0.98	0.09	NS
Gastrointestinal	OR	upper_SE	lower_SE	*p*-value	
Female	3.00	3.71	2.29	3.23E-06	****
GI Comorbidity	1.53	2.08	0.98	0.23	NS
WHO acute severity: class 6-9	0.95	1.18	0.73	0.83	NS
Age	1.00	1.01	0.99	0.86	NS
Cognitive impairment	OR	upper_SE	lower_SE	*p*-value	
Female	2.01	2.63	1.40	0.02	*
Neuro/Psych comorbidity	2.02	2.76	1.27	0.05	*
Age	1.02	1.03	1.01	0.14	NS
WHO acute severity: class 6-9	1.12	1.45	0.79	0.71	NS

Supplementary Table 3. The effect of clinical covariates on symptom outcome. The unadjusted odds ratio (OR) and standard error (SE) associated with clinical variables and each symptom outcome. An unadjusted, non-regularized logistic regression was used. The significance value from the binomial generalized linear model is shown: *=p<0·05, **=p<0·01, ***=p<0·001, ****=p<0·0001.

Healthy control demographics		
	Nasal fluid (n=25)	BALF (n=9)
COVID status	Samples collected prior to COVID-19 pandemic	Samples collected prior to COVID-19 pandemic
Age (years) Mean (SD)	39.5 (12.2)	38.1 (13.3)
Female sex n (%)	14 (56)	2 (22)
Non-smoker n (%)	25 (100)	9 (100)
White ethnicity n (%)	14 (56)	7 (77.8)

Supplementary Table 4. Demographics of healthy control participants. Nasal fluid from convalescent patients were compared to pre-pandemic nasal samples from healthy controls. Sputum from convalescent patients were compared to Broncho-alveolar lavage fluid (BALF) collected from individuals before the emergence of SARS-CoV-2. Data is shown as n (%) or mean (SD).

		Recovered (n=31)	Long COVID (n=33)	Missing data
Age, years Mean (SD)		59.32 (13.9)	54.8 (11.4)	5 (7.8)
Female sex n (%)		15 (50)	17 (58.6)	5 (7.8)
WHO acute severity n (%)	Class 3-4	4 (12.9)	7 (25)	5 (7.8)
	Class 5	18 (58.1)	11 (39.3)	
	Class 6	8 (25.8)	9 (32.1)	
	Class 7-9	1 (3.2)	1(3.6)	

Supplementary Table 5. Demographics of study participants with nasal inflammatory mediators measured (n=64). Data are n (% of cohort with complete data) or mean (SD). Missing data is shown as n (% of all individuals with inflammatory mediators measured).

		Recovered (n=16)	Long COVID (n=23)
Age, years Mean (SD)		57.5 (15.9)	61.7 (8.8)
Female sex n (%)		3 (18.8)	8 (34.8)
WHO acute severity n (%)	Class 3-4	4 (25.0)	6 (26.1)
	Class 5	7 (43.8)	4 (17.4)
	Class 6	3(18.8)	5 (21.7)
	Class 7-9	2 (12.5)	8 (34/8)

Supplementary Table 6. Demographics of study participants with Sputum samples available (n=40). Data are n (% of cohort with complete data) or mean (SD). There was no missing data in this cohort.

Symptom group	Questionnaire	Response type	Threshold score
Cardiorespiratory*	MRC dyspnoea scale	Numerical scale	Score >1
	Dyspnoea-12	Numerical scale	Score >= 12
	Palpitations	Yes/No	Yes
	Chest pain	Yes/No	Yes
Fatigue	FACIT Fatigue scale	Numerical scale	Score < 40
Anxiety/depression*	PHQ-9	Numerical scale	>= 10
	GAD-7	Numerical scale	>8
Cognitive impairment	Montreal Cognitive Assessment (MoCA)	Numerical scale	Score < 26
GI symptoms	Stomach pain	Yes/No	'Yes' to at least 2 symptoms.
	Constipation	Yes/No	
	Diarrhoea	Yes/No	
	Weight loss	Yes/No	
	Nausea and/or vomiting	Yes/No	
Recovered	Do you feel fully recovered from COVID-19?	Yes/No	Yes

Supplementary Table 7. **Clinical questionnaires used to define symptom groups.** The MoCA assessment was administered by a healthcare worker and the remaining scores were self-reported. All non-numerical scores were self-reported and patients were given the option to provide a binary response 'YES' or 'NO'. *Patients were placed in this symptom group if they met the threshold score for at least one variable in this category.

Supplementary Table 8. Univariate analysis of 368 Olink mediators measured. Each mediator was analysed, comparing values between recovered and symptom groups using the two-sided Wilcoxon-signed ranks test. The table is available in Appendix 1.

Supplementary Materials References:

1 Bio-techne. Human Luminex Discovery assay. https://www.rndsystems.com/products/human-luminex-discovery-assay_lxsahm. 2023.

2 Mesoscale discovery. R-Plex Human Follistatin Assay. https://www.mesoscale.com/en/products/r-plex-human-follistatin-assay-k151e5r/. 2023; published online April 20.

3 Mesoscale Discovery. Proinflammatory Panel 1 (human) kit. https://www.mesoscale.com/~/media/files/product%20inserts/proinflammatory%20panel%201%20human%20insert.pdf. 2023; published online April 20.

4 Higham A, Cadden P, Southworth T, *et al.* Leukotriene B4 levels in sputum from asthma patients. *ERJ Open Res* 2016; **2**: 00088–2015.

5 Ren A, Sohaei D, Ulndreaj A, *et al.* Ultrasensitive assay for saliva-based SARS-CoV-2 antigen detection. *Clinical Chemistry and Laboratory Medicine (CCLM)* 2022; **60**: 771–7.

6 Friedman J, Hastie T, Tibshirani R. Regularization Paths for Generalized Linear Models via Coordinate Descent. *J Stat Softw* 2010; **33**: 1–22.

7 Algamal ZY, Lee MH. Applying Penalized Binary Logistic Regression with Correlation Based Elastic Net for Variables Selection. *Journal of Modern Applied Statistical Methods* 2015; **14**: 168–79.

8 Greenwood CJ, Youssef GJ, Letcher P, *et al.* A comparison of penalised regression methods for informing the selection of predictive markers. *PLoS One* 2020; **15**: e0242730.

9 Xia Y. Correlation and association analyses in microbiome study integrating multiomics in health and disease. 2020: 309–491.

10 Firinguetti L, Kibria G, Araya R. Study of partial least squares and ridge regression methods. *Commun Stat Simul Comput* 2017; **46**: 6631–44.

11 Vorkapic E, Dugic E, Vikingsson S, *et al.* Imatinib treatment attenuates growth and inflammation of angiotensin II induced abdominal aortic aneurysm. *Atherosclerosis* 2016; **249**: 101–9.

12 Wolf AM, Wolf D, Rumpold H, *et al.* The kinase inhibitor imatinib mesylate inhibits TNF-α production *in vitro* and prevents TNF-dependent acute hepatic inflammation. *Proceedings of the National Academy of Sciences* 2005; **102**: 13622–7.

13 Kumar N, Goldminz AM, Kim N, Gottlieb AB. Phosphodiesterase 4-targeted treatments for autoimmune diseases. *BMC Med* 2013; **11**: 96.

14 Kalil AC, Stebbing J. Baricitinib: the first immunomodulatory treatment to reduce COVID-19 mortality in a placebo-controlled trial. *Lancet Respir Med* 2021; **9**: 1349–51.

15 Stebbing J, Krishnan V, de Bono S, *et al.* Mechanism of baricitinib supports artificial intelligence-predicted testing in <scp>COVID</scp> -19 patients. *EMBO Mol Med* 2020; **12**. DOI:10.15252/emmm.202012697.

16 Atalay S, Jarocka-Karpowicz I, Skrzydlewska E. Antioxidative and Anti-Inflammatory

Properties of Cannabidiol. *Antioxidants* 2019; **9**: 21.

17 Dong H, Liu F, Ma F, *et al.* Montelukast inhibits inflammatory response in rheumatoid arthritis fibroblast-like synoviocytes. *Int Immunopharmacol* 2018; **61**: 215–21.

18 Leung YY, Yao Hui LL, Kraus VB. Colchicine--Update on mechanisms of action and therapeutic uses. *Semin Arthritis Rheum* 2015; **45**: 341–50.

19 Bonilla H, Peluso MJ, Rodgers K, *et al.* Therapeutic trials for long COVID-19: A call to action from the interventions taskforce of the RECOVER initiative. *Front Immunol* 2023; **14**: 1129459.

Supplementary Table 8

	p value				
Assay	Cardiorespiratory	Fatigue	GI	Anxiety/ Depression	Cognitive
ACTN4	0.81050	0.79739	0.58587	0.81237	0.06346
ADA	0.88396	0.82789	0.58551	0.75619	0.49407
ADAM23	0.00682	0.00422	0.01834	0.00177	0.06026
ADGRE2	0.17803	0.27543	0.18735	0.39009	0.76286
AGER	0.15862	0.10577	0.29673	0.01256	0.19039
AGRN	0.92491	0.98035	0.95297	0.95531	0.91193
AGRP	0.41477	0.48829	0.19986	0.49593	0.56046
ALDH3A1	0.00247	0.00077	0.03121	0.00529	0.09081
AMBN	0.64560	0.73426	0.73014	0.91974	0.48710
AMN	0.12211	0.07642	0.54577	0.12229	0.32401
ANGPT1	0.43706	0.52978	0.30031	0.15403	0.41563
ANGPTL2	0.02766	0.02436	0.06211	0.00992	0.03074
ANGPTL4	0.13889	0.10379	0.21442	0.17346	0.93979
ANXA11	0.89512	0.79669	0.92964	0.75448	0.12503
AOC1	0.98301	0.91544	0.36056	0.70887	0.56934
ARHGEF12	0.68997	0.80717	0.82973	0.78684	0.22774
ARNT	0.42597	0.56220	0.69986	0.79074	0.24464
ARTN	0.86670	0.76789	0.99805	0.46119	0.75165
ATP5IF1	0.66350	0.84314	0.38571	0.82488	0.18530
AXIN1	0.89391	0.96149	0.89556	0.74744	0.40151
B4GALT1	0.16695	0.49533	0.59201	0.51084	0.85162
BACH1	0.50149	0.69635	0.65882	0.40903	0.12169
BANK1	0.81952	0.70486	0.96656	0.99961	0.51226
BCL2L11	0.25462	0.22514	0.16587	0.33901	0.89594
BCR	0.88511	0.97933	0.87956	0.81981	0.51556
BID	0.63722	0.60413	0.98084	0.78373	0.33113
BSG	0.86519	0.89895	0.48466	0.73836	0.55625
BTN2A1	0.69610	0.91296	0.99204	0.88575	0.41707
BTN3A2	0.80565	0.94480	0.37344	0.90235	0.27145
C1QA	0.28580	0.27994	0.08466	0.31555	0.02585
CASP2	0.44400	0.60466	0.62207	0.73586	0.36638
CCL11	0.33783	0.21097	0.24419	0.17599	0.10149
CCL13	0.04120	0.03952	0.12308	0.06138	0.32732
CCL17	0.24628	0.47709	0.23560	0.16263	0.54107
CCL20	0.29954	0.14704	0.19836	0.05802	0.22522
CCL21	0.77664	0.48870	0.39429	0.19249	0.34646
CCL22	0.10054	0.11941	0.03880	0.02273	0.08510
CCL23	0.54279	0.65320	0.23553	0.76989	0.49939
CCL24	0.92245	0.73379	0.86432	0.58731	0.35225
CCL25	0.81414	0.60697	0.90836	0.47221	0.19985
CCL26	0.21044	0.30852	0.81697	0.19416	0.11313
CCL28	0.11729	0.03393	0.74824	0.20374	0.99272
CCL3	0.98270	0.90274	0.73175	0.72319	0.94503
CCL4	0.55245	0.29619	0.84891	0.38402	0.57163
CCL7	0.50114	0.47034	0.66015	0.38114	0.32808
CCN2	0.54704	0.55934	0.69115	0.25212	0.57046
CD160	0.39574	0.25103	0.74370	0.45075	0.33703
CD200	0.11874	0.32724	0.38657	0.14413	0.30127
CD200R1	0.65203	0.74189	0.40629	0.81344	0.74297
CD22	0.37468	0.06172	0.07342	0.10772	0.98531
CD244	0.48867	0.36389	0.37025	0.60590	0.38787
CD276	0.14434	0.07477	0.56531	0.10443	0.07474
CD4	0.17890	0.16687	0.53573	0.47325	0.01188
CD40	0.95801	0.97678	0.71338	0.91925	0.50267
CD40LG	0.96187	0.91378	0.63722	0.73298	0.42694
CD48	0.28831	0.73922	0.80353	0.37027	0.92249
CD58	0.02520	0.04096	0.02191	0.06637	0.07476
CD6	0.89146	0.57233	0.64187	0.86662	0.76058
CD70	0.79132	0.95848	0.51491	0.73210	0.98354
CD79B	0.34973	0.52888	0.78955	0.47941	0.70611
CD83	0.21333	0.11968	0.23895	0.13765	0.24831
CD84	0.74242	0.81049	0.90736	0.81703	0.27693
CDON	0.60270	0.71023	0.08399	0.53347	0.39713
CDSN	0.52534	0.58049	0.37917	0.24733	0.72132
CEACAM21	0.69865	0.88692	0.83027	0.79077	0.83411
CEP164	0.13441	0.08125	0.32200	0.31197	0.07347
CHRDL1	0.89258	0.93295	0.76300	0.69617	0.11433
CKAP4	0.67591	0.59597	0.97523	0.80857	0.33515
CKMT1A/B	0.77070	0.57203	0.89919	0.60048	0.19389
CLEC4A	0.66043	0.61813	0.92430	0.70278	0.10373
CLEC4C	0.36007	0.10612	0.16437	0.04408	0.00633
CLEC4D	0.11614	0.04899	0.05339	0.02222	0.05771
CLEC4G	0.16728	0.21228	0.33311	0.19296	0.30057
CLEC7A	0.90046	0.94651	0.54976	0.58563	0.60461
CLIP2	0.79828	0.87317	0.86357	0.76390	0.23238
CLSTN2	0.44331	0.26365	0.47066	0.18189	0.48481
CNTNAP2	0.32335	0.20585	0.08791	0.16280	0.53206
COL9A1	0.91103	0.92553	0.31561	0.82217	0.09467
COLEC12	0.08993	0.07017	0.40892	0.15165	0.06520
CRELD2	0.91940	0.81340	0.74264	0.92743	0.97800
CRHBP	0.27119	0.21505	0.33588	0.16515	0.60723
CRIM1	0.71658	0.94817	0.68905	0.86767	0.48201
CRKL	0.84533	0.91145	0.79392	0.88601	0.16880
CRLF1	0.69610	0.68395	0.33959	0.46239	0.50872
CSF1	0.95188	0.47032	0.27476	0.58141	0.55176
CSF3	0.05212	0.02783	0.00109	0.04287	0.52887
CST7	0.74867	0.81811	0.72906	0.85536	0.23753
CTRC	0.15276	0.05795	0.01973	0.31245	0.72123
CTSC	0.42579	0.42549	0.73370	0.60916	0.32719
CTSO	0.13435	0.09827	0.86001	0.33220	0.03305
CXADR	0.40214	0.36947	0.66200	0.50014	0.01361
CXCL1	0.71475	0.70001	0.98919	0.70939	0.18498
CXCL10	0.52192	0.42895	0.59748	0.62459	0.78184
CXCL12	0.84298	0.94123	0.44174	0.99609	0.92844
CXCL14	0.60216	0.70596	0.17801	0.82781	0.35683
CXCL17	0.29083	0.51517	0.28501	0.27376	0.78870
CXCL3	0.89611	0.92349	0.76151	0.94010	0.25128
CXCL6	0.38225	0.27391	0.60203	0.16911	0.18681
CXCL8	0.92946	0.82929	0.63684	0.61213	0.26703
CXCL9	0.25703	0.27071	0.83730	0.28595	0.37746
DAG1	0.61444	0.69272	0.80763	0.77870	0.48989
DAPP1	0.64370	0.81907	0.65425	0.85852	0.32896

	p value				
Assay	Cardiorespiratory	Fatigue	GI	Anxiety/ Depression	Cognitive
DBNL	0.76030	0.87345	0.77722	0.96391	0.21269
DECR1	0.99616	0.92540	0.80225	0.95365	0.64329
DFFA	0.90772	0.83129	0.95000	0.91430	0.15786
DGKZ	0.82598	0.88376	0.82069	0.99150	0.70551
DNAJA2	0.97392	0.87844	0.99916	0.77042	0.20067
DNER	0.00003	0.00001	0.01092	0.00032	0.00944
DNPH1	0.47652	0.53786	0.42248	0.32019	0.07042
DPP10	0.11769	0.17225	0.14428	0.23071	0.00120
EDAR	0.67918	0.96758	0.40317	0.44669	0.38539
EGF	0.75595	0.92703	0.52980	0.73981	0.46932
EGLN1	0.92596	0.96095	0.67836	0.75284	0.35623
EIF4G1	0.61444	0.73940	0.85761	0.71377	0.19182
EIF5A	0.91448	0.67234	0.51616	0.65895	0.81210
ENAH	0.03529	0.03732	0.18537	0.13088	0.47099
ENPP5	0.00180	0.00111	0.00332	0.01386	0.26779
ENPP7	0.36629	0.08887	0.36062	0.02311	0.58890
EPCAM	0.51881	0.22133	0.39332	0.47327	0.04468
EPHA1	0.73446	0.74895	0.72429	0.74395	0.92558
EPO	0.11360	0.07243	0.04896	0.13858	0.18916
ERBB3	0.21359	0.07990	0.17998	0.03600	0.57568
ESM1	0.04378	0.09839	0.57950	0.11824	0.44494
F2R	0.86867	0.89939	0.93347	0.96558	0.19518
FABP1	0.09780	0.04344	0.18692	0.10097	0.19690
FABP9	0.81897	0.46472	0.11185	0.76814	0.79631
FASLG	0.48894	0.94121	0.33906	0.93614	0.72449
FCAR	0.11046	0.08793	0.11389	0.01831	0.14251
FCRL2	0.00293	0.10854	0.32256	0.02807	0.01962
FCRL3	0.77297	0.60954	0.62518	0.99689	0.41049
FCRL6	0.24074	0.12174	0.09997	0.12150	0.74445
FGF19	0.25839	0.34651	0.43930	0.66190	0.04267
FGF2	0.64817	0.55188	0.49732	0.76860	0.20144
FGF5	0.17195	0.22741	0.62300	0.21645	0.16461
FIS1	0.82480	0.80470	0.98766	0.68592	0.32881
FKBP1B	0.56154	0.63221	0.80949	0.59658	0.34283
FLT3LG	0.25954	0.31118	0.08794	0.30724	0.77572
FOXO1	0.55474	0.58116	0.81734	0.67558	0.28818
FST	0.09078	0.05310	0.00669	0.02038	0.27647
FSTL3	0.23858	0.11635	0.36917	0.33868	0.72562
FXYD5	0.82539	0.78012	0.90285	0.95870	0.16845
GAL	0.01603	0.02869	0.10848	0.03077	0.16354
GALNT3	0.25258	0.27420	0.14132	0.35037	0.47725
GBP2	0.80051	0.82359	0.84081	0.59038	0.30437
GLOD4	0.61767	0.70342	0.59001	0.62362	0.07496
GMPR	0.66064	0.89587	0.64895	0.81251	0.12164
GOPC	0.95990	0.92676	0.77377	0.98700	0.28983
GZMA	0.86758	0.99520	0.47443	0.90308	0.50017
GZMB	0.64036	0.58856	0.29889	0.85265	0.38703
HCLS1	0.39968	0.38747	0.43445	0.47268	0.29080
HEXIM1	0.89562	0.77487	0.86576	0.83054	0.21701
HGF	0.06673	0.08213	0.16002	0.05715	0.16600
HLA-DRA	0.45796	0.44349	0.17830	0.33423	0.31156
HLA-E	0.39252	0.40686	0.66894	0.23763	0.23019
HPCAL1	0.96253	0.97496	0.90708	0.84741	0.21785
HSD11B1	0.49529	0.32016	0.83609	0.65609	0.19206
HSPA1A	0.89465	0.95714	0.75364	0.95257	0.19635
ICA1	0.79437	0.68853	0.76627	0.73118	0.41204
ICAM4	0.00707	0.00217	0.00019	0.01482	0.21762
IDS	0.04207	0.00685	0.05753	0.02714	0.84990
IFNG	0.77626	0.65786	0.18128	0.91349	0.69687
IFNGR1	0.10281	0.19129	0.19401	0.18824	0.85291
IFNLR1	0.12425	0.14114	0.08654	0.18509	0.99994
IKBKG	0.83311	0.92925	0.94088	0.82683	0.26364
IL10	0.35088	0.45059	0.35165	0.50533	0.42664
IL10RA	0.46051	0.48410	0.08872	0.52353	0.28862
IL10RB	0.81198	0.70537	0.78142	0.70190	0.87329
IL11	0.47436	0.36722	0.33577	0.40359	0.30424
IL12B	0.02439	0.01848	0.09372	0.02292	0.08305
IL12RB1	0.54960	0.86952	0.78623	0.95107	0.11312
IL13	0.39729	0.59793	0.96109	0.77746	0.90692
IL15	0.65545	0.38851	0.88991	0.70165	0.80262
IL15RA	0.28688	0.57540	0.70160	0.21707	0.65753
IL16	0.59918	0.35501	0.66511	0.76015	0.15824
IL17A	0.44131	0.85921	0.53035	0.57366	0.06560
IL17C	0.73756	0.92777	0.43157	0.76296	0.93863
IL17D	0.98945	0.56410	0.15484	0.92585	0.48894
IL17F	0.19861	0.22145	0.24855	0.32190	0.00482
IL17RB	0.68807	0.80511	0.53850	0.58016	0.20951
IL18	0.80079	0.94748	0.75615	0.77879	0.88351
IL18R1	0.03539	0.01671	0.19124	0.03602	0.71183
IL1A	0.42265	0.08455	0.17520	0.09415	0.28182
IL1B	0.51424	0.56077	0.98701	0.55059	0.45982
IL1R2	0.00607	0.00061	0.05391	0.00021	0.22766
IL1RL2	0.07020	0.01231	0.06117	0.10291	0.15499
IL1RN	0.08592	0.02898	0.18443	0.02180	0.61730
IL2	0.00461	0.00437	0.32610	0.01797	0.10340
IL20	0.75320	0.74782	0.99652	0.94728	0.21397
IL20RA	0.02912	0.02579	0.01513	0.00611	0.05010
IL22RA1	0.71832	0.84800	0.90914	0.94268	0.86560
IL24	0.11303	0.14630	0.17197	0.24022	0.51283
IL2RB	0.37711	0.64271	0.51092	0.91406	0.76761
IL32	0.56962	0.70641	0.49781	0.88340	0.09267
IL33	0.30230	0.66156	0.49291	0.94038	0.56826
IL3RA	0.71710	0.84522	0.82663	0.99573	0.38109
IL4	0.73380	0.86763	0.83791	0.91104	0.34974
IL4R	0.58148	0.98232	0.59560	0.94100	0.27248
IL5	0.32461	0.64367	0.76792	0.35519	0.63577
IL5RA	0.04583	0.04989	0.10622	0.01435	0.71320
IL6	0.00864	0.00558	0.00039	0.00220	0.04438
IL7	0.38783	0.54027	0.53250	0.18562	0.38897
IRAK1	0.88895	0.79620	0.98977	0.69511	0.36582
IRAK4	0.81657	0.82785	0.96926	0.66349	0.16427
ISM1	0.01991	0.02421	0.05177	0.14262	0.34449

	p value				
Assay	Cardiorespiratory	Fatigue	GI	Anxiety/ Depression	Cognitive
ITGA11	0.02069	0.00920	0.00501	0.04576	0.21897
ITGA6	0.30610	0.25969	0.46493	0.41824	0.57787
ITGB6	0.10529	0.01330	0.06800	0.02263	0.57576
ITM2A	0.43392	0.44025	0.03707	0.27952	0.90769
JCHAIN	0.21242	0.30833	0.33755	0.40020	0.29174
JUN	0.60591	0.73770	0.90186	0.54598	0.41410
KLRB1	0.87824	0.54583	0.91096	0.63741	0.98693
KLRD1	0.90485	0.62739	0.85535	0.82808	0.22846
KRT19	0.30288	0.62243	0.52674	0.57093	0.11763
KYNU	0.55920	0.72687	0.29571	0.67721	0.87732
LAIR1	0.92249	0.77773	0.85505	0.95134	0.62270
LAMA4	0.85870	0.90148	0.90888	0.97350	0.83613
LAMP3	0.30580	0.44366	0.54161	0.16982	0.89200
LAP3	0.76175	0.95626	0.85006	0.96818	0.20408
LAT	0.84431	0.75217	0.72128	0.57258	0.25189
LGALS4	0.01838	0.00542	0.03572	0.01047	0.00147
LGALS9	0.11114	0.04762	0.06717	0.04943	0.07784
LGMN	0.39877	0.53812	0.99461	0.83329	0.20442
LHPP	0.65095	0.66877	0.51794	0.32508	0.47082
LIFR	0.90884	0.85492	0.71904	0.89604	0.10127
LILRB4	0.67327	0.58763	0.65037	0.81122	0.88355
LRRN1	0.08750	0.02525	0.02424	0.00854	0.01628
LSP1	0.76357	0.85187	0.39659	0.80851	0.32617
LTA	0.63965	0.39778	0.30801	0.61455	0.57550
LTBR	0.76518	0.99299	0.90932	0.91671	0.55356
LTO1	0.79820	0.82546	0.46830	0.84860	0.83647
LY6D	0.21545	0.21938	0.29174	0.22260	0.75401
LY75	0.71989	0.99188	0.66118	0.85668	0.37517
LY9	0.92118	0.68786	0.75061	0.70852	0.40427
MANF	0.79915	0.87669	0.46672	0.85531	0.15420
MAP2K6	0.57041	0.63270	0.82128	0.75942	0.12603
MAPK9	0.72599	0.96203	0.74085	0.82960	0.06634
MATN2	0.01206	0.01031	0.03138	0.02093	0.03121
MEGF10	0.00017	0.00038	0.02219	0.00720	0.11492
MEPE	0.18730	0.20809	0.08553	0.14826	0.47345
MERTK	0.58114	0.60019	0.57539	0.91007	0.14572
METAP1D	0.76510	0.73720	0.71416	0.68247	0.73618
MGLL	0.75241	0.79834	0.55968	0.70176	0.21015
MGMT	0.75524	0.68917	0.90932	0.93366	0.50860
MICB_MICA	0.06119	0.03011	0.06513	0.05108	0.01171
MILR1	0.75601	0.66411	0.38046	0.42320	0.24704
MLN	0.87811	0.92700	0.80281	0.70079	0.59679
MMP1	0.56133	0.48272	0.42385	0.24432	0.89028
MMP10	0.09400	0.30559	0.83999	0.35975	0.19312
MPIG6B	0.40646	0.45821	0.24364	0.21387	0.14228
MVK	0.14364	0.14770	0.25437	0.13883	0.20066
MYO9B	0.78521	0.85919	0.96601	0.99187	0.51251
MZB1	0.63531	0.38378	0.49808	0.68225	0.94476
NBN	0.76327	0.99320	0.45041	0.79945	0.28917
NCF2	0.32005	0.15862	0.70966	0.21631	0.02094
NCK2	0.77806	0.79743	0.84187	0.89728	0.46623
NCLN	0.82640	0.55088	0.26151	0.79362	0.47800
NCR1	0.65426	0.46432	0.55430	0.71656	0.21773
NELL2	0.93322	0.87735	0.90673	0.94929	0.60370
NFASC	0.11402	0.13756	0.60848	0.20852	0.01012
NFATC1	0.83033	0.72949	0.84810	0.90230	0.48961
NFATC3	0.18531	0.09128	0.79878	0.20567	0.79379
NME3	0.90832	0.86319	0.31323	0.67748	0.61244
NPPC	0.15353	0.08467	0.08569	0.21631	0.15566
NRTN	0.85238	0.92649	0.53710	0.85157	0.64691
NT5C3A	0.96389	0.95847	0.95710	0.93194	0.28298
NTF3	0.14486	0.02463	0.05635	0.03163	0.88393
NUB1	0.74942	0.74881	0.58672	0.64988	0.28091
NUDC	0.89480	0.82721	0.81287	0.84820	0.21648
OMD	0.01523	0.00541	0.01163	0.02909	0.75947
OSCAR	0.88233	0.52878	0.53727	0.25819	0.05449
OSM	0.14775	0.08471	0.15268	0.02379	0.07551
PADI2	0.62563	0.67072	0.94648	0.73700	0.02732
PAPPA	0.17062	0.20938	0.15975	0.37382	0.05782
PARP1	0.71163	0.98301	0.52005	0.75854	0.47351
PCDH1	0.60032	0.82133	0.42114	0.42426	0.54729
PDGFB	0.42090	0.52451	0.32719	0.19921	0.48825
PDLIM7	0.85548	0.99885	0.87105	0.86580	0.41433
PGF	0.34472	0.30658	0.42319	0.40064	0.95694
PIK3AP1	0.45544	0.47249	0.50780	0.36755	0.05370
PKLR	0.21342	0.21689	0.27021	0.09388	0.27253
PLA2G4A	0.84274	0.74442	0.85506	0.80485	0.32050
PLAUR	0.36019	0.28372	0.20596	0.30307	0.16388
PLXNA4	0.61133	0.74286	0.60984	0.68445	0.27100
PNLIPRP2	0.48718	0.80891	0.45599	0.26328	0.20843
PNPT1	0.79183	0.91934	0.81462	0.81073	0.42459
PON3	0.21361	0.35802	0.55270	0.75240	0.83239
PPP1R9B	0.47268	0.47455	0.46531	0.46252	0.38686
PRDX3	0.91954	0.90862	0.88432	0.86943	0.24940
PRDX5	0.79181	0.71555	0.77104	0.80286	0.12720
PREB	0.64555	0.90374	0.69069	0.88193	0.23439
PRELP	0.07758	0.08238	0.09589	0.08794	0.34416
PRKAB1	0.98417	0.99182	0.84935	0.60733	0.56202
PRKCQ	0.75517	0.46957	0.94443	0.66466	0.80422
PROK1	0.00608	0.00029	0.03947	0.00378	0.03596
PRSS8	0.05267	0.07074	0.06217	0.05590	0.32261
PSIP1	0.74734	0.90812	0.48122	0.71728	0.34844

	p value				
Assay	Cardiorespiratory	Fatigue	GI	Anxiety/ Depression	Cognitive
PSMG3	0.59224	0.58945	0.47407	0.43241	0.18487
PSPN	0.00259	0.00285	0.00180	0.00616	0.03989
PTH1R	0.25401	0.38421	0.97826	0.38693	0.35331
PTPN6	0.60653	0.69023	0.84636	0.72392	0.20520
PTPRM	0.23476	0.07688	0.10490	0.16590	0.41513
PTX3	0.75589	0.41666	0.95209	0.43829	0.69219
RAB37	0.88470	0.87649	0.81690	0.67719	0.17653
RAB6A	0.97520	0.94274	0.96032	0.94476	0.36601
RABGAP1L	0.98844	0.97635	0.95317	0.84260	0.19538
REG4	0.68286	0.31687	0.12733	0.84751	0.17746
RGS8	0.82331	0.74095	0.73522	0.98689	0.85045
ROBO1	0.03838	0.02554	0.12806	0.07874	0.29629
SAMD9L	0.87420	0.74742	0.83245	0.77114	0.16409
SCG3	0.93718	0.85195	0.14041	0.75227	0.42805
SCGB1A1	0.44360	0.38557	0.46258	0.27588	0.30943
SCGB3A2	0.96155	0.86672	0.68536	0.83138	0.61205
SCGN	0.52949	0.81290	0.10211	0.51879	0.19738
SCRN1	0.74156	0.56827	0.44599	0.56541	0.14316
SELPLG	0.36191	0.16550	0.57032	0.11894	0.57895
SERPINB8	0.42102	0.33358	0.83533	0.46359	0.04986
SH2D1A	0.91131	0.96395	0.46210	0.65606	0.46568
SHMT1	0.18048	0.26767	0.37989	0.13829	0.12490
SIGLEC1	0.44610	0.31174	0.28337	0.66789	0.66497
SIGLEC10	0.52200	0.20845	0.26175	0.56077	0.08764
SIRPB1	0.46665	0.80279	0.72181	0.67306	0.84651
SIT1	0.61131	0.30405	0.41240	0.33330	0.30168
SKAP2	0.64336	0.80111	0.55535	0.76536	0.21322
SLAMF1	0.95180	0.74811	0.11661	0.32008	0.34002
SLAMF7	0.44555	0.14955	0.90323	0.66600	0.71165
SLC39A5	0.03029	0.01306	0.33622	0.05671	0.25510
SMOC2	0.10717	0.04595	0.01478	0.09379	0.41787
SMPDL3A	0.77568	0.63580	0.71227	0.58013	0.02731
SPINK4	0.51873	0.48568	0.56985	0.79871	0.01662
SPINT2	0.64418	0.69247	0.85748	0.85859	0.58541
SPON1	0.98106	0.64321	0.92487	0.68975	0.08693
SPRY2	0.48255	0.47472	0.67408	0.54911	0.57848
SRPK2	0.47795	0.55024	0.34905	0.42289	0.48260
STX8	0.78760	0.83901	0.85177	0.93394	0.49488
SULT2A1	0.04511	0.03213	0.21337	0.06697	0.06940
TANK	0.58516	0.70978	0.66931	0.44914	0.30607
TBC1D5	0.29964	0.40877	0.57374	0.51282	0.16596
TFF2	0.09708	0.04517	0.01031	0.02007	0.02108
TGFA	0.19841	0.11956	0.10182	0.08391	0.02977
TGFB1	0.27964	0.43854	0.30544	0.23597	0.21768
TIMP3	0.22704	0.25343	0.35341	0.13253	0.08427
TLR3	0.45321	0.22843	0.68210	0.24247	0.66446
TNF	0.91569	0.65629	0.87709	0.83025	0.05782
TNFAIP8	0.26630	0.27259	0.93154	0.21632	0.43621
TNFRSF11A	0.86222	0.84006	0.70302	0.66859	0.84593
TNFRSF11B	0.98227	0.79912	0.09663	0.93828	0.00679
TNFRSF13B	0.89710	0.65602	0.57291	0.91834	0.62816
TNFRSF13C	0.00971	0.05458	0.28992	0.06921	0.08504
TNFRSF14	0.79302	0.89118	0.81197	0.74853	0.58764
TNFRSF4	0.50628	0.24408	0.40606	0.48060	0.39722
TNFSF10	0.38120	0.66831	0.03206	0.45883	0.79044
TNFSF11	0.19441	0.29855	0.00027	0.31662	0.21489
TNFSF12	0.26095	0.23096	0.43051	0.51900	0.69420
TNFSF13	0.09543	0.09251	0.42452	0.13854	0.73670
TPP1	0.81574	0.77232	0.38706	0.37092	0.45114
TPSAB1	0.09264	0.09192	0.09515	0.49904	0.25362
TPT1	0.72096	0.38019	0.33301	0.28821	0.97819
TRAF2	0.47667	0.70580	0.67011	0.79399	0.19724
TREM2	0.16274	0.08434	0.42661	0.13103	0.39964
TRIM21	0.42562	0.33020	0.14077	0.41659	0.57323
TRIM5	0.71690	0.82289	0.79073	0.83015	0.13667
VASH1	0.63406	0.61275	0.76057	0.59458	0.63513
VEGFA	0.71890	0.72726	0.23710	0.23747	0.77450
VEGFD	0.07368	0.06108	0.45856	0.07470	0.63667
WAS	0.78464	0.94263	0.68391	0.90455	0.57179
WFIKKN2	0.00366	0.00954	0.16165	0.00430	0.81605
WNT9A	0.27161	0.27970	0.53304	0.09789	0.71853
YTHDF3	0.52855	0.65617	0.65820	0.61448	0.53743

PHOSP-COVID Collaborative Group

Core Management Group

Chief Investigator C E Brightling, *Members* R A Evans (Lead Co-I), L V Wain (Lead Co-I), J D Chalmers, V C Harris, L P Ho, A Horsley, M Marks, K Poinasamy, B Raman, A Shikotra, A Singapuri

PHOSP-COVID Study Central Coordinating Team

C E Brightling (Chief Investigator), R A Evans (*Lead Co-I*), L V Wain (*Lead Co-I*), R Dowling, C Edwardson, O Elneima, S Finney, N J Greening, B Hargadon, V C Harris, L Houchen--Wolloff, O C Leavy, H J C McAuley, C Overton, T Plekhanova, R M Saunders, M Sereno, A Singapuri, A Shikotra, C Taylor, S Terry, C Tong, B Zhao

Steering Committee

Co-chairs D Lomas, E Sapey, *Institution representatives* C Berry, C E Bolton, N Brunskill, E R Chilvers, R Djukanovic, Y Ellis, D Forton, N French, J George, N A Hanley, N Hart, L McGarvey, N Maskell, H McShane, M Parkes, D Peckham, P Pfeffer, A Sayer, A Sheikh, A A R Thompson, N Williams and core management group representation

Executive Board

Chair C E Brightling, representation from the core management group, each working group and platforms

Platforms

Bioresource

W Greenhalf (*Co-Lead*), M G Semple (*Co-Lead*), M Ashworth, H E Hardwick, L Lavelle-Langham, W Reynolds, M Sereno, R M Saunders, A Singapuri, V Shaw, A Shikotra, B Venson, L V Wain

Data Hub

A B Docherty (*Co-Lead*), E M Harrison (*Co-Lead*), A Sheikh (*Co-Lead*), J K Baillie, C E Brightling, L Daines, R Free, R A Evans, S Kerr, O C Leavy, N I Lone, H J C McAuley, R Pius, J Quint, M Richardson, M Sereno, M Thorpe, L V Wain

Imaging Alliance

M Halling-Brown (*Co-Lead*), F Gleeson (*Co-Lead*), J Jacob (*Co-Lead*), S Neubauer (*Co-Lead*) B Raman (*Co-Lead*) S Siddiqui (*Co-Lead*) J M Wild (*Co-Lead*), S Aslani, M Beggs, M P Cassar, A Chiribiri, E Cox, D J Cuthbertson, V M Ferreira, L Finnigan, S Francis, P Jezzard, G J Kemp, H Lamlum, W Lilaonitkul, E Lukaschuk, C Manisty, G P McCann , C McCracken, K McGlynn , R Menke , C A Miller , A J Moss, T E Nichols, C Nikolaidou , C O'Brien , G Ogbole, D P O'Regan , S Piechnik , S Plein, I Propescu, A A Samat, L Saunders, Z B Sanders, R Steeds, T Treibel, E M Tunnicliffe, M Webster, J Willoughby, J Weir McCall, C Xie.

Omics

ISARIC4C Consortium members

Name	affiliation1
J Kenneth Baillie	Roslin Institute, University of Edinburgh, Easter Bush, Edinburgh, EH25 9RG, UK
Peter JM Openshaw	National Heart and Lung Institute, Imperial College London, London, UK
Malcolm G Semple	NIHR Health Protection Research Unit, Institute of Infection, Veterinary and Ecological Sciences, Faculty of Health and Life Sciences, University of Liverpool, Liverpool, UK
Beatrice Alex	School of Informatics, University of Edinburgh, Edinburgh, UK
Petros Andrikopoulos	Section of Biomolecular Medicine, Division of Systems Medicine, Department of Metabolism, Digestion and Reproduction, Sir Alexander Fleming Building, Exhibition Rd, London SW7 2AZ, UK
Benjamin Bach	School of Informatics, University of Edinburgh, Edinburgh, UK
Wendy S Barclay	Section of Molecular Virology, Imperial College London, London, UK
Debby Bogaert	Centre for Inflammation Research, The Queen's Medical Research Institute, University of Edinburgh, 47 Little France Crescent, Edinburgh, UK
Meera Chand	Antimicrobial Resistance and Hospital Acquired Infection Department, Public Health England, London, UK
Kanta Chechi	Department of Epidemiology and Biostatistics, School of Public Health, Faculty of Medicine, Imperial College London, 2 Norfolk St, W2 1PG, UK
Graham S Cooke	Department of Infectious Disease, Imperial College London, London, UK
Ana da Silva Filipe	MRC-University of Glasgow Centre for Virus Research, 464 Bearsden Road, Glasgow, UK
Thushan de Silva	The Florey Institute for Host-Pathogen Interactions, Department of Infection, Immunity and Cardiovascular Disease, University of Sheffield, Sheffield, UK
Annemarie B Docherty	Centre for Medical Informatics, The Usher Institute, University of Edinburgh, Edinburgh, UK
Gonçalo dos Santos Correia	National Phenome Centre, Department of Metabolism, Digestion and Reproduction, Imperial College London, London W12 0NN, United Kingdom
Marc-Emmanuel Dumas	Section of Biomolecular Medicine, Division of Systems Medicine, Department of Metabolism, Digestion and Reproduction, Sir Alexander Fleming Building, Exhibition Rd, London SW7 2AZ, UK
Jake Dunning	National Infection Service, Public Health England, London, UK
Tom Fletcher	Liverpool School of Tropical Medicine, Liverpool, UK

Christoper A Green	Institute of Microbiology and Infection, University of Birmingham, Birmingham, UK
William Greenhalf	Department of Molecular and Clinical Cancer Medicine, University of Liverpool, Liverpool, UK
Julian L Griffin	Section of Biomolecular Medicine, Division of Systems Medicine, Department of Metabolism, Digestion and Reproduction, Sir Alexander Fleming Building, Exhibition Rd, London SW7 2AZ, UK
Rishi K Gupta	Institute for Global Health, University College London, London, UK
Ewen M Harrison	Centre for Medical Informatics, The Usher Institute, University of Edinburgh, Edinburgh, UK
Antonia Ying Wai Ho	MRC-University of Glasgow Centre for Virus Research, 464 Bearsden Road, Glasgow, UK
Karl Holden	University of Liverpool
Peter W Horby	Centre for Tropical Medicine and Global Health,
Samreen Ijaz	Virology Reference Department, National Infection Service, Public Health England, Colindale Avenue, London, United Kingdom
Saye Khoo	Department of Pharmacology, University of Liverpool, Liverpool, UK
Paul Klenerman	Nuffield Department of Medicine, Peter Medawar Building for Pathogen Research, University of Oxford, UK
Andrew Law	The Roslin Institute, University of Edinburgh, Edinburgh, UK
Matthew R Lewis	National Phenome Centre, Department of Metabolism, Digestion and Reproduction, Imperial College London, London W12 0NN, United Kingdom
Sonia Liggi	Section of Biomolecular Medicine, Division of Systems Medicine, Department of Metabolism, Digestion and Reproduction, Sir Alexander Fleming Building, Exhibition Rd, London SW7 2AZ, UK
Wei Shen Lim	Nottingham University Hospitals NHS Trust: Nottingham
Lynn Maslen	National Phenome Centre, Department of Metabolism, Digestion and Reproduction, Imperial College London, London W12 0NN, United Kingdom
Alexander J Mentzer	Nuffield Department of Medicine, John Radcliffe Hospital, Oxford, United Kingdom
Laura Merson	ISARIC Global Support Centre, Centre for Tropical Medicine and Global Health, Nuffield Department of Medicine, University of Oxford, Oxford, UK
Alison M Meynert	MRC Human Genetics Unit, MRC Institute of Genetics and Molecular Medicine, University of Edinburgh, Edinburgh, UK
Shona C Moore	Institute of Infection, Veterinary and Ecological Sciences, University of Liverpool, Liverpool, UK

Mahdad Noursadeghi	Division of Infection and Immunity, University College London, UK
Michael Olanipekun	Section of Biomolecular Medicine, Division of Systems Medicine, Department of Metabolism, Digestion and Reproduction, Sir Alexander Fleming Building, Exhibition Rd, London SW7 2AZ, UK
Anthonia Osagie	Section of Biomolecular Medicine, Division of Systems Medicine, Department of Metabolism, Digestion and Reproduction, Sir Alexander Fleming Building, Exhibition Rd, London SW7 2AZ, UK
Massimo Palmarini	MRC-University of Glasgow Centre for Virus Research, 464 Bearsden Road, Glasgow, UK
Carlo Palmieri	Molecular and Clinical Cancer Medicine, Institute of Systems, Molecular and Integrative Biology, University of Liverpool
William A Paxton	Institute of Infection, Veterinary and Ecological Sciences, University of Liverpool, Liverpool, UK
Georgios Pollakis	Institute of Infection, Veterinary and Ecological Sciences, University of Liverpool, Liverpool, UK
Nicholas Price	Centre for Clinical Infection and Diagnostics Research, Department of Infectious Diseases, School of Immunology and Microbial Sciences, King's College London, London, UK
Andrew Rambaut	Institute of Evolutionary Biology, University of Edinburgh, Edinburgh, UK
David L Robertson	MRC-University of Glasgow Centre for Virus Research, 464 Bearsden Road, Glasgow, UK
Clark D Russell	Centre for Inflammation Research, The Queen's Medical Research Institute, University of Edinburgh, 47 Little France Crescent, Edinburgh, UK
Vanessa Sancho-Shimizu	Department of Pediatrics and Virology, St Mary's Medical School Bldg, Imperial College London, London, UK
Caroline J Sands	National Phenome Centre, Department of Metabolism, Digestion and Reproduction, Imperial College London, London W12 0NN, United Kingdom
Janet T Scott	MRC-University of Glasgow Centre for Virus Research, 464 Bearsden Road, Glasgow, UK
Louise Sigfrid	ISARIC Global Support Centre, Centre for Tropical Medicine and Global Health, Nuffield Department of Medicine, University of Oxford, Oxford, UK
Tom Solomon	NIHR Health Protection Research Unit, Institute of Infection, Veterinary and Ecological Sciences, Faculty of Health and Life Sciences, University of Liverpool, Liverpool, UK
Shiranee Sriskandan	Department of Infectious Disease, Imperial College London, London, UK

David Stuart	Division of Structural Biology, The Wellcome Centre for Human Genetics, University of Oxford, Headington, Oxford, OX3 7BN, UK
Charlotte Summers	Department of Medicine, University of Cambridge, Cambridge, Cambridgeshire, UK
Olivia V Swann	Department of Child Life and Health, University of Edinburgh, Edinburgh, UK
Zoltan Takats	Section of Biomolecular Medicine, Division of Systems Medicine, Department of Metabolism, Digestion and Reproduction, Sir Alexander Fleming Building, Exhibition Rd, London SW7 2AZ, UK
Panteleimon Takis	National Phenome Centre, Department of Metabolism, Digestion and Reproduction, Imperial College London, London W12 0NN, United Kingdom
Richard S Tedder	Blood Borne Virus Unit, Virus Reference Department, National Infection Service, Public Health England, London, UK
AA Roger Thompson	Department of Infection, Immunity and Cardiovascular Disease, University of Sheffield, Sheffield, UK
Emma C Thomson	MRC-University of Glasgow Centre for Virus Research, 464 Bearsden Road, Glasgow, UK
Ryan S Thwaites	National Heart and Lung Institute, Imperial College London, London, UK
Lance CW Turtle	NIHR Health Protection Research Unit, Institute of Infection, Veterinary and Ecological Sciences, Faculty of Health and Life Sciences, University of Liverpool, Liverpool, UK
Maria Zambon	National Infection Service, Public Health England, London, UK
Thomas M Drake	Centre for Medical Informatics, The Usher Institute, University of Edinburgh, Edinburgh, UK
Cameron J Fairfield	Centre for Medical Informatics, The Usher Institute, University of Edinburgh, Edinburgh, UK
Stephen R Knight	Centre for Medical Informatics, The Usher Institute, University of Edinburgh, Edinburgh, UK
Kenneth A Mclean	Centre for Medical Informatics, The Usher Institute, University of Edinburgh, Edinburgh, UK
Derek Murphy	Centre for Medical Informatics, The Usher Institute, University of Edinburgh, Edinburgh, UK
Lisa Norman	Centre for Medical Informatics, The Usher Institute, University of Edinburgh, Edinburgh, UK
Riinu Pius	Centre for Medical Informatics, The Usher Institute, University of Edinburgh, Edinburgh, UK
Catherine A Shaw	Centre for Medical Informatics, The Usher Institute, University of Edinburgh, Edinburgh, UK
Marie Connor	Liverpool Clinical Trials Centre, University of Liverpool, Liverpool, UK

Jo Dalton	Liverpool Clinical Trials Centre, University of Liverpool, Liverpool, UK
Carrol Gamble	Liverpool Clinical Trials Centre, University of Liverpool, Liverpool, UK
Michelle Girvan	Liverpool Clinical Trials Centre, University of Liverpool, Liverpool, UK
Sophie Halpin	Liverpool Clinical Trials Centre, University of Liverpool, Liverpool, UK
Janet Harrison	Liverpool Clinical Trials Centre, University of Liverpool, Liverpool, UK
Clare Jackson	Liverpool Clinical Trials Centre, University of Liverpool, Liverpool, UK
James Lee	ISARIC, Global Support Centre, COVID-19 Clinical Research Resources, Epidemic diseases Research Group, Oxford (ERGO), University of Oxford
Laura Marsh	Liverpool Clinical Trials Centre, University of Liverpool, Liverpool, UK
Daniel Plotkin	ISARIC, Global Support Centre, COVID-19 Clinical Research Resources, Epidemic diseases Research Group, Oxford (ERGO), University of Oxford
Stephanie Roberts	Liverpool Clinical Trials Centre, University of Liverpool, Liverpool, UK
Egle Saviciute	Liverpool Clinical Trials Centre, University of Liverpool, Liverpool, UK
Sara Clohisey	Roslin Institute, University of Edinburgh, Easter Bush, Edinburgh, EH25 9RG, UK
Ross Hendry	Roslin Institute, University of Edinburgh, Easter Bush, Edinburgh, EH25 9RG, UK
Susan Knight	Public Health Scotland
Eva Lahnsteiner	
Andrew Law	Roslin Institute, University of Edinburgh, Easter Bush, Edinburgh, EH25 9RG, UK

Gary Leeming	Centre for Health Informatics, Division of Informatics, Imaging and Data Science, School of Health Sciences, Faculty of Biology, Medicine and Health, University of Manchester, Manchester Academic Health Science Centre, Manchester, UK
Lucy Norris	EPCC, University of Edinburgh, Edinburgh, UK
James Scott-Brown	School of Informatics, University of Edinburgh, Edinburgh, UK
Sarah Tait	Public Health Scotland
Murray Wham	MRC Human Genetics Unit, MRC Institute of Genetics and Molecular Medicine, University of Edinburgh, Edinburgh, UK
Richard Clark	
Audrey Coutts	
Lorna Donnelly	
Angie Fawkes	
Tammy Gilchrist	
Katarzyna Hafezi	
Louise MacGillivray	
Alan Maclean	
Sarah McCafferty	
Kirstie Morrice	
Lee Murphy	
Nicola Wrobel	
Gail Carson	ISARIC Global Support Centre, Centre for Tropical Medicine and Global Health, Nuffield Department of Medicine, University of Oxford, Oxford, UK
Kayode Adeniji	
Daniel Agranoff	
Ken Agwuh	
Dhiraj Ail	
Erin L. Aldera	
Ana Alegria	
Sam Allen	
Brian Angus	
Abdul Ashish	
Dougal Atkinson	
Shahedal Bari	
Gavin Barlow	
Stella Barnass	

Nicholas Barrett
Christopher Bassford
Sneha Basude
David Baxter
Michael Beadsworth
Jolanta Bernatoniene
John Berridge
Colin Berry
Nicola Best
Pieter Bothma
Robin Brittain-Long
Naomi Bulteel
Tom Burden
Andrew Burtenshaw
Vikki Caruth
David Chadwick
David Chadwick
Duncan Chambler
Nigel Chee
Jenny Child
Srikanth Chukkambotla
Tom Clark
Paul Collini
Catherine Cosgrove
Jason Cupitt
Maria-Teresa Cutino-Moguel
Paul Dark
Chris Dawson
Samir Dervisevic
Phil Donnison
Sam Douthwaite
Andrew Drummond
Ingrid DuRand
Ahilanadan Dushianthan
Tristan Dyer
Cariad Evans
Chi Eziefula
Chrisopher Fegan
Adam Finn
Duncan Fullerton
Sanjeev Garg
Sanjeev Garg
Atul Garg
Effrossyni Gkrania-Klotsas
Jo Godden
Arthur Goldsmith
Clive Graham

Tassos Grammatikopoulos	Paediatric Liver, GI & Nutrition Centre and MowatLabs, King’s College Hospital, London, UK

Elaine Hardy
Stuart Hartshorn
Daniel Harvey
Peter Havalda
Daniel B Hawcutt
Maria Hobrok
Luke Hodgson
Anil Hormis
Joanne Howard
Michael Jacobs
Susan Jain
Paul Jennings
Agilan Kaliappan
Vidya Kasipandian
Stephen Kegg
Michael Kelsey
Jason Kendall
Caroline Kerrison
Ian Kerslake
Oliver Koch
Gouri Koduri
George Koshy
Shondipon Laha
Steven Laird
Susan Larkin
Tamas Leiner
Patrick Lillie
James Limb
Vanessa Linnett
Jeff Little
Mark Lyttle
Michael MacMahon
Emily MacNaughton
Ravish Mankregod
Huw Masson
Elijah Matovu
Katherine McCullough
Ruth McEwen
Manjula Meda
Gary Mills
Jane Minton
Kavya Mohandas
Quen Mok
James Moon
Elinoor Moore
Patrick Morgan
Craig Morris
Katherine Mortimore
Samuel Moses
Mbiye Mpenge
Rohinton Mulla
Michael Murphy

Thapas Nagarajan
Megan Nagel
Mark Nelson
Lillian Norris
Matthew K. O'Shea
Marlies Ostermann
Igor Otahal
Mark Pais
Carlo Palmieri University of Liverpool
Selva Panchatsharam
Danai Papakonstantinou
Padmasayee Papineni
Hassan Paraiso
Brij Patel
Natalie Pattison
Justin Pepperell
Mark Peters
Mandeep Phull
Stefania Pintus
Tim Planche
Frank Post
David Price
Rachel Prout
Nikolas Rae
Henrik Reschreiter
Tim Reynolds
Neil Richardson
Mark Roberts
Devender Roberts
Alistair Rose
Guy Rousseau
Bobby Ruge
Brendan Ryan
Taranprit Saluja
Sarah Cole
Matthias L Schmid
Aarti Shah
Manu Shankar-Hari
Prad Shanmuga
Anil Sharma
Anna Shawcross
Jagtur Singh Pooni
Jeremy Sizer
Richard Smith
Catherine Snelson
Nick Spittle
Nikki Staines
Tom Stambach
Richard Stewart
Pradeep Subudhi
Tamas Szakmany
Kate Tatham

Jo Thomas
Chris Thompson
Robert Thompson
Ascanio Tridente
Darell Tupper-Carey
Mary Twagira
Nick Vallotton
Rama Vancheeswaran
Rachel Vincent
Lisa Vincent-Smith
Shico Visuvanathan
Alan Vuylsteke
Sam Waddy
Rachel Wake
Andrew Walden
Ingeborg Welters
Tony Whitehouse
Paul Whittaker
Ashley Whittington
Meme Wijesinghe
Martin Williams
Lawrence Wilson
Stephen Winchester
Martin Wiselka
Adam Wolverson
Daniel G Wootton
Andrew Workman
Bryan Yates
Peter Young

Sarah E McDonald — MRC-University of Glasgow Centre for Virus Research, 464 Bearsden Road, Glasgow, UK

Victoria Shaw — Institute of Translational Medicine, University of Liverpool, Liverpool, Merseyside, United Kingdom
Katie A. Ahmed
Jane A Armstrong
Milton Ashworth
Innocent G Asiimwe
Siddharth Bakshi
Samantha L Barlow
Laura Booth
Benjamin Brennan
Katie Bullock
Nicola Carlucci
Emily Cass
Benjamin WA Catterall
Jordan J Clark
Emily A Clarke
Sarah Cole
Louise Cooper
Helen Cox
Christopher Davis
Oslem Dincarslan

Alejandra Doce Carracedo
Chris Dunn
Philip Dyer
Angela Elliott
Anthony Evans
Lorna Finch
Lewis WS Fisher
Lisa Flaherty
Terry Foster
Isabel Garcia-Dorival
Philip Gunning
Catherine Hartley
Anthony Holmes
Rebecca L Jensen
Christopher B Jones
Trevor R Jones
Shadia Khandaker
Katharine King
Robyn T. Kiy
Chrysa Koukorava
Annette Lake
Suzannah Lant
Diane Latawiec
Lara Lavelle-Langham
Daniella Lefteri
Lauren Lett
Lucia A Livoti
Maria Mancini
Hannah Massey
Nicole Maziere
Sarah McDonald
Laurence McEvoy
John McLauchlan
Soeren Metelmann
Nahida S Miah
Joanna Middleton
Joyce Mitchell
Shona C Moore
Ellen G Murphy
Rebekah Penrice-Randal
Jack Pilgrim
Tessa Prince
Will Reynolds
P. Matthew Ridley
Debby Sales
Victoria E Shaw
Rebecca K Shears
Benjamin Small
Krishanthi S Subramaniam
Agnieska Szemiel
Aislynn Taggart
Jolanta Tanianis-Hughes

Jordan Thomas	
Erwan Trochu	
Libby van Tonder	
Eve Wilcock	
J. Eunice Zhang	
Seán Keating	Intensive Care Unit, Royal Infirmary Edinburgh, Edinburgh, UK
Cara Donegan	Institute of Infection, Veterinary and Ecological Sciences, Faculty of Health and Life Sciences, University of Liverpool, Liverpool, UK
Rebecca G. Spencer	Institute of Infection, Veterinary and Ecological Sciences, Faculty of Health and Life Sciences, University of Liverpool, Liverpool, UK
Chloe Donohue	Liverpool Clinical Trials Centre, University of Liverpool, Liverpool, UK
Fiona Griffiths	Roslin Institute, University of Edinburgh, Easter Bush, Edinburgh, EH25 9RG, UK
Hayley Hardwick	NIHR Health Protection Research Unit, Institute of Infection, Veterinary and Ecological Sciences, Faculty of Health and Life Sciences, University of Liverpool, Liverpool, UK
Wilna Oosthuyzen	Roslin Institute, University of Edinburgh, Easter Bush, Edinburgh, EH25 9RG, UK

L V Wain (*Co-Lead)*, J K Baillie (*Co-Lead*), H Baxendale, C E Brightling, M Brown, J D Chalmers, R A Evans, B Gooptu, W Greenhalf, H E Hardwick, R G Jenkins, D Jones, I Koychev, C Langenberg, A Lawrie, P L Molyneaux, A Shikotra, J Pearl, M Ralser, N Sattar, R M Saunders, J T Scott, T Shaw, D Thomas, D Wilkinson

Working Groups

Airways

L G Heaney (*Co-Lead*), A De Soyza (*Co-Lead*), D Adeloye, C E Brightling, J S Brown, J Busby, J D Chalmers, C Echevarria, L Daines, O Elneima, RA Evans, J Hurst, P Novotny, P Pfeffer, K Poinasamy, J Quint, I Rudan, E Sapey, M Shankar-Hari, A Sheikh, S Siddiqui, S Walker, B Zheng

Brain

J R Geddes (*Lead*), M Hotopf *(Co-Lead),* K Abel, R Ahmed, L Allan, C Armour, D Baguley, D Baldwin, C Ballard, K Bhui, G Breen, M Broome, T Brugha, E Bullmore, D Burn, F Callard, J Cavanagh, T Chalder, D Clark, A David, B Deakin, H Dobson, B Elliott, J Evans, R Francis, E Guthrie, P Harrison, M Henderson, A Hosseini, N Huneke, M Husain, T Jackson, I Jones, T Kabir, P Kitterick, A Korszun, I Koychev, J Kwan, A Lingford-Hughes, P Mansoori, H McAllister-Williams, K McIvor, L Milligan, R Morriss, E Mukaetova-Ladinska, K Munro, A Nevado-Holgado, T Nicholson, S Paddick, C Pariante, J Pimm, K Saunders, M Sharpe, G Simons, R Upthegrove, S Wessely

Cardiac

G P McCann (*Lead*), S Amoils, C Antoniades, A Banerjee, R Bell, A Bularga, C Berry, P Chowienczyk, J P Greenwood, A D Hughes, K Khunti, L Kingham, C Lawson, K Mangion, N L Mills, A J Moss, S Neubauer, B Raman, A N Sattar, C L Sudlow, M Toshner,

Immunology

P J M Openshaw (*Lead*), D Altmann, J K Baillie, R Batterham, H Baxendale, N Bishop, C E Brightling, P C Calder, R A Evans, J L Heeney, T Hussell, P Klenerman, J M Lord, P Moss, S L Rowland-Jones, W Schwaeble, M G Semple, R S Thwaites, L Turtle, L V Wain, S Walmsley, D Wraith

Intensive Care

M J Rowland (*Lead*), A Rostron (*Co-Lead*), J K Baillie, B Connolly, A B Docherty, N I Lone, D F McAuley, D Parekh, A Rostron, J Simpson, C Summers

Lung Fibrosis

R G Jenkins (*Co-Lead*), J Porter (*Co-Lead*), R J Allen, R Aul, J K Baillie, S Barratt, P Beirne, J Blaikley, R C Chambers, N Chaudhuri, C Coleman, E Denneny, L Fabbri, P M George, M Gibbons, F Gleeson, B Gooptu, B Guillen Guio, I Hall, N A Hanley, L P Ho, E Hufton, J Jacob, I Jarrold, G Jenkins, S Johnson, M G Jones, S Jones, F Khan, P Mehta, J Mitchell, P L Molyneaux, J E Pearl, K Piper Hanley, K Poinasamy, J Quint, D Parekh, P Rivera-Ortega, L C Saunders, M G Semple, J Simpson, D Smith, M Spears, L G Spencer, S Stanel, I Stewart, A A R Thompson, D Thickett, R Thwaites, L V Wain, S Walker, S Walsh, J M Wild, D G Wootton, L Wright

Metabolic

S Heller (*Co-Lead*), M J Davies (*Co-Lead*), H Atkins, S Bain, J Dennis, K Ismail, D Johnston, P Kar, K Khunti, C Langenberg, P McArdle, A McGovern, T Peto, J Petrie, E Robertson, N Sattar, K Shah, J Valabhji, B Young

Pulmonary and Systematic Vasculature

L S Howard (*Co-Lead*), Mark Toshner (*Co-Lead*), C Berry, P Chowienczyk, D Lasserson, A Lawrie, O C Leavy, J Mitchell, J Newman, L Price, J Quint, A Reddy, J Rossdale, N Sattar, C Sudlow, A A R Thompson, J M Wild, M Wilkins

Rehabilitation, Sarcopenia and Fatigue

S J Singh (*Co-Lead*), W D-C Man (*Co-Lead*), J M Lord (*Co-Lead*), N J Greening (*Co-Lead*), T Chalder (*Co-Lead*), J T Scott (*Co-Lead*), N Armstrong, E Baldry, M Baldwin, N Basu, M Beadsworth, L Bishop, C E Bolton, A Briggs, M Buch, G Carson, J Cavanagh, H Chinoy, E Daynes, S Defres, R A Evans, P Greenhaff, S Greenwood, M Harvie, M Husain, S MacDonald, A McArdle, H J C McAuley, A McMahon, M McNarry, G Mills, C Nolan, K O'Donnell, D Parekh, Pimm, J Sargent, L Sigfrid, M Steiner, D Stensel, A L Tan, J Whitney, D Wilkinson, D Wilson, M Witham, D G Wootton, T Yates

Renal

D Thomas (*Lead*), N Brunskill (*Co-Lead*), S Francis (*Co-Lead*), S Greenwood (*Co-Lead*), C Laing (*Co-Lead*), K Bramham, P Chowdhury, A Frankel, L Lightstone, S McAdoo, K McCafferty, M Ostermann, N Selby, C Sharpe, M Willicombe

Patient Public Engagement Group

L Houchen-Wolloff (*Lead*), J Bunker, R Gill, C Hastie, R Nathu, N Rogers, N Smith

Local Clinical Centre PHOSP-COVID trial staff

(listed in alphabetical order)

Airedale NHS Foundation Trust

A Shaw (PI), L Armstrong, B Hairsine, H Henson, C Kurasz, L Shenton

Aneurin Bevan University Health Board

S Fairbairn (PI), A Dell, N Hawkings, J Haworth, M Hoare, A Lucey, V Lewis, G Mallison, H Nassa, C Pennington, A Price, C Price, A Storrie, G Willis, S Young

Barts Health NHS Trust & Queen Mary University of London

P Pfeffer (PI), K Chong-James, C David, W Y James, C Manisty, A Martineau, O Zongo

Barnsley Hospital NHS Foundation Trust

A Sanderson (PI)

Belfast Health and Social Care Trust & Queen's University Belfast

L G Heaney (PI), C Armour, V Brown, T Craig, S Drain, B King, N Magee, D McAulay, E Major, L McGarvey, J McGinness, R Stone

Betsi Cadwaladr University Health Board

A Haggar (PI), A Bolger, F Davies, J Lewis, A Lloyd, R Manley, E McIvor, D Menzies, K Roberts, W Saxon, D Southern, C Subbe, V Whitehead

Borders General Hospital, NHS Borders

H El-Taweel (PI), J Dawson, L Robinson

Bradford Teaching Hospitals NHS Foundation Trust

D Saralaya (PI), L Brear, K Regan, K Storton

Cambridge University Hospitals NHS Foundation Trust, NIHR Cambridge Clinical Research Facility & University of Cambridge

J Fuld (PI), A Bermperi, I Cruz, K Dempsey, A Elmer, H Jones, S Jose, S Marciniak, M Parkes, C Ribeiro, J Taylor, M Toshner, L Watson, J Weir McCall, J Worsley

Cardiff and Vale University Health Board

R Sabit (PI), L Broad, A Buttress, T Evans, M Haynes, L Jones, L Knibbs, A McQueen, C Oliver, K Paradowski, J Williams

Chesterfield Royal Hospital NHS Trust

E Harris (PI), C Sampson

Cwm Taf Morgannwg University Health Board

C Lynch (PI), E Davies, C Evenden , A Hancock, K Hancock, M Rees , L Roche, N Stroud, T Thomas-Woods

East Cheshire NHS Trust

M Babores (PI), J Bradley-Potts, M Holland, N Keenan, S Shashaa, H Wassall

East Kent Hospitals University NHS Foundation Trust

E Beranova (PI), H Weston (PI), T Cosier, L Austin, J Deery, T Hazelton, C Price, H Ramos, R Solly, S Turney

Gateshead NHS Trust

L Pearce (PI), W McCormick, S Pugmire, W Stoker, A Wilson

Guy's and St Thomas' NHS Foundation Trust

N Hart (PI), LA Aguilar Jimenez, G Arbane, S Betts, K Bisnauthsing, A Dewar, P Chowdhury, A Chiribiri, A Dewar, G Kaltsakas, H Kerslake, MM Magtoto, P Marino, LM Martinez, C O'Brien, M Ostermann, J Rossdale, TS Solano, E Wynn

Hampshire Hospitals NHS Foundation Trust

N Williams (PI), W Storrar (PI), M Alvarez Corral, A Arias, E Bevan, D Griffin, J Martin, J Owen,

S Payne, A Prabhu, A Reed, C Wrey Brown

Harrogate and District NHD Foundation Trust

C Lawson (PI), T Burdett, J Featherstone, A Layton, C Mills, L Stephenson,

Hull University Teaching Hospitals NHS Trust & University of Hull

N Easom (PI), P Atkin, K Brindle, M G Crooks, K Drury, R Flockton, L Holdsworth, A Richards, D L Sykes, S Thackray-Nocera, C Wright

Hywel Dda University Health Board

K E Lewis (PI), A Mohamed (PI), G Ross (PI), S Coetzee, K Davies, R Hughes, R Loosley, L O'Brien, Z Omar, H McGuinness, E Perkins, J Phipps, A Taylor, H Tench, R Wolf-Roberts

Imperial College Healthcare NHS Trust & Imperial College London

L S Howard (PI), O Kon (PI), D C Thomas (PI), S Anifowose, L Burden, E Calvelo, B Card, C Carr, E R Chilvers, D Copeland, P Cullinan, P Daly, L Evison, T Fayzan, H Gordon, S Haq, R G Jenkins, C King, K March, M Mariveles, L McLeavey, N Mohamed, S Moriera, U Munawar, J Nunag, U Nwanguma, L Orriss-Dib, D P O'Regan, A Ross, M Roy, E Russell, K Samuel, J Schronce, N Simpson, L Tarusan, C Wood, N Yasmin

Kettering General Hospital NHS Trust

R Reddy (PI), A-M, Guerdette, M Hewitt, K Warwick, S White

King's College Hospital NHS Foundation Trust & Kings College London

A M Shah (PI), C J Jolley (PI), O Adeyemi, R Adrego, H Assefa-Kebede, J Breeze, M Brown, S Byrne, T Chalder, A Chiribiri, P Dulawan, N Hart, A Hayday, A Hoare, A Knighton, M Malim, C O'Brien, S Patale, I Peralta, N Powell, A Ramos, K Shevket, F Speranza, A Te

Leeds Teaching Hospitals & University of Leeds

P Beirne (PI), A Ashworth, J Clarke, C Coupland, M Dalton, E Wade, C Favager, J Greenwood, J Glossop, L Hall, T Hardy, A Humphries, J Murira, D Peckham, S Plein, J Rangeley, G Saalmink, A L Tan, B Whittam, N Window, J Woods,

Lewisham & Greenwich NHS Trust

G Coakley (PI)

Liverpool University Hospitals NHS Foundation Trust & University of Liverpool

D G Wootton (PI), L Turtle (PI), L Allerton, AM All, M Beadsworth, A Berridge, J Brown, S Cooper, A Cross, D J Cuthbertson, S Defres, S L Dobson, J Earley, N French, W Greenhalf, H E Hardwick, K Hainey, J Hawkes, V Highett, S Kaprowska, G J Kemp, AL Key, S Koprowska, L Lavelle-Langham, N Lewis-Burke, G Madzamba, F Malein, S Marsh, C Mears, L Melling, M J Noonan, L Poll, J Pratt, E Richardson, A Rowe, M G Semple, V Shaw, K A Tripp, B Vinson, L O Wajero, S A Williams-Howard, J Wyles

London North West University Healthcare NHS Trust

S N Diwanji (PI), P Papineni (PI), S Gurram, S Quaid, G F Tiongson, E Watson

Manchester University NHS Foundation Trust & University of Manchester

B Al-Sheklly (PI), A Horsley (PI), C Avram, J Blaikely, M Buch, N Choudhury, D Faluyi, T Felton, T Gorsuch, N A Hanley, T Hussell, Z Kausar, C A Miller, N Odell, R Osbourne, K Piper Hanley, K Radhakrishnan, S Stockdale

Newcastle upon Tyne Hospitals NHS Foundation Trust & University of Newcastle

A De Soyza (PI), C Echevarria (PI), A Ayoub, J Brown, G Burns, G Davies, H Fisher, C Francis, A Greenhalgh, P Hogarth, J Hughes, K Jiwa, G Jones, G MacGowan, D Price, A Sayer, J Simpson, H Tedd, S Thomas, S West, M Witham, S Wright, A Young

NHS Dumfries and Galloway

M J McMahon (PI), P Neill

NHS Greater Glasgow and Clyde Health Board & University of Glasgow

D Anderson (PI), H Bayes (PI), C Berry (PI), D Grieve (PI), I B McInnes (PI), N Basu, A Brown, A Dougherty, K Fallon, L Gilmour, K Mangion, A Morrow, K Scott, R Sykes, R Touyz

NHS Highland

E K Sage (PI), F Barrett, A Donaldson

NHS Lanarkshire

M Patel (PI), D Bell, A Brown, M Brown, R Hamil, K Leitch, L Macliver, J Quigley, A Smith, B Welsh

NHS Lothian & University of Edinburgh

G Choudhury (PI), J K Baillie, S Clohisey, A Deans, A B Docherty, J Furniss, E M Harrison, S Kelly, N I Lone, D E Newby, A Sheikh

NHS Tayside & University of Dundee

J D Chalmers (PI), D Connell, A Elliott, C Deas, J George, S Mohammed, J Rowland, A R Solstice, D Sutherland, C J Tee

North Bristol NHS Trust & University of Bristol

N Maskell (PI), D Arnold, S Barrett, H Adamali, A Dipper, S Dunn, A Morley, L Morrison, L Stadon, S Waterson, H Welch

North Middlesex Hospital NHS Trust

B Jayaraman (PI), T Light

Nottingham University Hospitals NHS Trust & University of Nottingham

C E Bolton (PI), P Almeida, J Bonnington, M Chrystal, E Cox, C Dupont, S Francis, P Greenhaff, A Gupta, L Howard, W Jang, S Linford, L Matthews, R Needham, A Nikolaidis, S Prosper, K Shaw, A K Thomas

Oxford University Hospitals NHS Foundation Trust & University of Oxford

L P Ho (PI), N M Rahman (PI), M Ainsworth, A Alamoudi, M Beggs, A Bates, A Bloss, A Burns, P Carter, M Cassar, K M Channon, J Chen, F Conneh, T Dong, R I Evans, E Fraser, X Fu, J R Geddes, F Gleeson, P Harrison, M Havinden-Williams, P Jezzard, N Kanellakis, I Koychev, P Kurupati, X Li, E Lukaschuk, K McGlynn, H McShane, C Megson, K Motohashi, S Neubauer, D Nicoll, G Ogg, E Pacpaco, M Pavlides, Y Peng, N Petousi, J Propescu, N Rahman, B Raman, M J Rowland, K Saunders, M Sharpe, N Talbot, E Tunnicliffe

Royal Brompton and Harefield Clinical Group, Guy's and St Thomas' NHS Foundation Trust.

W D-C Man (PI), B Patel (PI), R E Barker, D Cristiano, N Dormand, M Gummadi, S Kon, K Liyanage, C M Nolan, S Patel, O Polgar, P Shah, S J Singh, J A Walsh

Royal Free London NHS Foundation Trust

J Hurst (PI), H Jarvis (PI), S Mandal (PI), S Ahmad, S Brill, L Lim, D Matila, O Olaosebikan, C Singh

Royal Papworth Hospital NHS Foundation Trust

M Toshner (PI), H Baxendale, L Garner, C Johnson, J Mackie, A Michael, J Pack, K Paques, H Parfrey, J Parmar

Salford Royal NHS Foundation Trust

N Diar Bakerly (PI), P Dark, D Evans, E Hardy, A Harvey, D Holgate, S Knight, N Mairs, N Majeed, L McMorrow, J Oxton, J Pendlebury, C Summersgill, R Ugwuoke, S Whittaker

Salisbury NHS Foundation Trust

W Matimba-Mupaya (PI), S Strong-Sheldrake

Sheffield Teaching NHS Foundation Trust & University of Sheffield

S L Rowland-Jones (PI), A A R Thompson (Co PI), J Bagshaw, M Begum, K Birchall, R Butcher, H Carborn, F Chan, K Chapman, Y Cheng, L Chetham, C Clark, Z Coburn, J Cole, M Dixon, A Fairman, J Finnigan, L Finnigan, H Foot, D Foote, A Ford, R Gregory, K Harrington, L Haslam, L Hesselden, J Hockridge, A Holbourn, B Holroyd-Hind, L Holt, A Howell, E Hurditch, F Ilyas, C Jarman, A Lawrie, E Lee, J-H Lee, R Lenagh, A Lye, I Macharia, M Marshall, A Mbuyisa, J McNeill, S Megson, J Meiring, L Milner, S Misra, H Newell, T Newman, C Norman, L Nwafor, D Pattenadk, M Plowright, J Porter, P Ravencroft, C Roddis, J Rodger, P Saunders, J Sidebottom, J Smith, L Smith, N Steele, G Stephens, R Stimpson, B Thamu, N Tinker, K Turner, H Turton, P Wade, S Walker, J Watson, J M Wild, I Wilson, A Zawia

St George's University Hospitals NHS Foundation Trust

R Aul (PI), M Ali, A Dunleavy (PI), D Forton, N Msimanga, M Mencias, T Samakomva, S Siddique, J Teixeira, V Tavoukjian

Sherwood Forest Hospitals NHS Foundation Trust

J Hutchinson (PI), L Allsop, K Bennett, P Buckley, M Flynn, M Gill, C Goodwin, M Greatorex, H Gregory, C Heeley, L Holloway, M Holmes, J Kirk, W Lovegrove, TA Sewell, S Shelton, D Sissons, K Slack, S Smith, D Sowter, S Turner, V Whitworth, I Wynter

Shropshire Community Health NHS Trust

L Warburton (PI), S Painter, J Tomlinson

Somerset NHS Foundation Trust

C Vickers (PI), T Wainwright, D Redwood, J Tilley, S Palmer

Swansea Bay University Health Board

G A Davies (PI), L Connor, A Cook, T Rees, F Thaivalappil, C Thomas

Tameside and Glossop Integrated Care NHS Foundation

A Butt (PI), M Coulding, H Jones, S Kilroy, J McCormick, J McIntosh, H Savill, V Turner, J Vere

The Great Western Hospital Foundation Trust

E Fraile (PI), J Ugoji

The Hillingdon Hospitals NHS Foundation Trust

S S Kon (PI), H Lota, G Landers, M Nasseri, S Portukhay

The Rotherham NHS Foundation Trust

A Hormis (PI), A Daniels, J Ingham, L Zeidan

United Lincolnshire Hospitals NHS Trust

M Chablani (PI), L Osborne

University College London Hospital & University College London

M Marks (PI), J S Brown (PI), N Ahwireng, B Bang, D Basire, R C Chambers, A Checkley, R Evans, M Heightman, T Hillman, J Hurst, J Jacob, S Janes, R Jastrub, M Lipman, S Logan, D Lomas, M Merida Morillas, A Pakzad, H Plant, J C Porter, K Roy, E Wall, B Williams, M Xu

University Hospital Birmingham NHS Foundation Trust & University of Birmingham

D Parekh (PI), N Ahmad Haider, C Atkin, R Baggott, M Bates, A Botkai, A Casey, B Cooper, J Dasgin, K Draxlbauer, N Gautam, J Hazeldine, T Hiwot, S Holden, K Isaacs, T Jackson, S Johnson, V Kamwa, D Lewis, J M Lord, S Madathil, C McGhee, K Mcgee, A Neal, A Newton Cox, J Nyaboko, D Parekh, Z Peterkin, H Qureshi, B Rangelov, L Ratcliffe, E Sapey, J Short, T Soulsby, R Steeds, J Stockley, Z Suleiman, T Thompson, M Ventura, S Walder, C Welch, D Wilson, S Yasmin, K P Yip

University Hospitals of Derby and Burton

P Beckett (PI) C Dickens, U Nanda

University Hospitals of Leicester NHS Trust & University of Leicester

C E Brightling (CI), R A Evans (PI), M Aljaroof, N Armstrong, H Arnold, H Aung, M Bakali, M Bakau, M Baldwin, M Bingham, M Bourne, C Bourne, N Brunskill, P Cairns, L Carr, A Charalambou, C Christie, M J Davies, S Diver, S Edwards, C Edwardson, O Elneima, H Evans, J Finch, S Glover, N Goodman, B Gootpu, N J Greening, K Hadley, P Haldar, B Hargadon, V C Harris, L Houchen-Wolloff, W Ibrahim, L Ingram, K Khunti, A Lea, D Lee, G P McCann, H J C McAuley, P McCourt, T Mcnally, G Mills, A Moss, W Monteiro, M Pareek, S Parker, A Rowland, A Prickett, I N Qureshi, R Russell, N Samani, M Sereno, M Sharma, A Shikotra, S Siddiqui, A Singapuri, S J Singh, J Skeemer, M Soares, E Stringer, T Thornton, M Tobin, E Turner, L V Wain, T J C Ward, F Woodhead, J Wormleighton, T Yates, A Yousuf

University Hospital Southampton NHS Foundation Trust & University of Southampton

M G Jones (PI), C Childs, R Djukanovic, S Fletcher, M Harvey, E Marouzet, B Marshall, R Samuel, T Sass, T Wallis, H Wheeler

Whittington Health NHS

R Dharmagunawardena (PI), E Bright, P Crisp, M Stern

Wirral University Teaching Hospital

A Wight (PI), L Bailey, A Reddington

Wrightington Wigan and Leigh NHS trust

A Ashish (PI), J Cooper, E Robinson

Yeovil District Hospital NHS Foundation Trust

A Broadley (PI)

York & Scarborough NHS Foundation Trust

K Howard (PI), L Barman, C Brookes, K Elliott. L Griffiths, Z Guy, D Ionita, H Redfearn, C Sarginson A Turnbull

Health and Care Research Wales

Y Ellis

London School of Hygiene & Tropical Medicine (LSHTM)

M Marks, A Briggs

NIHR Office for Clinical Research Infrastructure

K Holmes

Patient Public Involvement Leads

Asthma UK and British Lung Foundation Partnership - K Poinasamy, S Walker

Royal Surrey NHS Foundation Trust

M Halling-Brown

South London and Maudsley NHS Foundation Trust & Kings College London

G Breen, M Hotopf

Swansea University & Swansea Welsh Network

K Lewis, N Williams
