# Supplementary material for: Large-scale phenotyping of patients with long COVID post-hospitalization reveals mechanistic subtypes of disease
Source: Nat Immunol. 2024 Apr 8;25(4):607–21. doi: 10.1038/s41590-024-01778-0 (PMC11003868; doi:10.1038/s41590-024-01778-0)
Supplement: Supplementary file 2 — Reporting Summary [file 41590_2024_1778_MOESM2_ESM.pdf]

Corresponding author(s): Dr Ryan S Thwaites; Prof Peter Openshaw

Last updated by author(s): 1/02/2024

Reporting Summary

Nature Portfolio wishes to improve the reproducibility of the work that we publish. This form provides structure for consistency and transparency in reporting. For further information on Nature Portfolio policies, see our Editorial Policies and the Editorial Policy Checklist.

Statistics

For all statistical analyses, confirm that the following items are present in the figure legend, table legend, main text, or Methods section.

n/a	Confirmed	
☐	☒	The exact sample size (*n*) for each experimental group/condition, given as a discrete number and unit of measurement
☐	☒	A statement on whether measurements were taken from distinct samples or whether the same sample was measured repeatedly
☐	☒	The statistical test(s) used AND whether they are one- or two-sided<br>*Only common tests should be described solely by name; describe more complex techniques in the Methods section.*
☐	☒	A description of all covariates tested
☐	☒	A description of any assumptions or corrections, such as tests of normality and adjustment for multiple comparisons
☐	☒	A full description of the statistical parameters including central tendency (e.g. means) or other basic estimates (e.g. regression coefficient) AND variation (e.g. standard deviation) or associated estimates of uncertainty (e.g. confidence intervals)
☐	☒	For null hypothesis testing, the test statistic (e.g. *F*, *t*, *r*) with confidence intervals, effect sizes, degrees of freedom and *P* value noted<br>*Give P values as exact values whenever suitable.*
☒	☐	For Bayesian analysis, information on the choice of priors and Markov chain Monte Carlo settings
☒	☐	For hierarchical and complex designs, identification of the appropriate level for tests and full reporting of outcomes
☒	☐	Estimates of effect sizes (e.g. Cohen's *d*, Pearson's *r*), indicating how they were calculated

Our web collection on statistics for biologists contains articles on many of the points above.

Software and code

Policy information about availability of computer code

Data collection	No software or algorithms were used. Code used to generate summary data in the Outbreak Analysis Platform (ODAP) is publicly available here: https://github.com/isaric4c/wiki/wiki/ISARIC and https://github.com/SurgicalInformatics/cocin_ccp
Data analysis	code was written within the Outbreak Analysis Platform (ODAP) , to which access is available under reasonable request as per the Data sharing statement in the manuscript. Analyses within the ODAP were performed in R v4.2.0 using publicly available packages listed in the manuscript methods (‘data.table v1.14.2’, ‘EnvStats v2.7.0’ ‘tidyverse v1.3.2’, ‘lme4 v1.1-32’, ‘caret v6.0-93’, ‘glmnet v4.1-6’, ‘mdatools v0.14.0’, ‘ggpubbr v0.4.0’ , ‘ggplot2 v3.3.6’, ‘bootnet v1.5.6’ and ‘qgraph v1.9.8’ packages).<br>Extended data Figure 10 was made in Biorender by accessing biorender.com. Last access was Dec 2023. No new algorithms or functions were created for the purposes of analysing the datasets. Code used to generate figures used standard functions in listed packages that are available on CRAN. Code is available at https://github.com/isaric4c/wiki/wiki/ISARIC and https://github.com/SurgicalInformatics/cocin_ccp and https://github.com/ClaudiaEfstath/PHOSP_Olink_NatImm.

For manuscripts utilizing custom algorithms or software that are central to the research but not yet described in published literature, software must be made available to editors and reviewers. We strongly encourage code deposition in a community repository (e.g. GitHub). See the Nature Portfolio guidelines for submitting code & software for further information.

Data

Policy information about availability of data

All manuscripts must include a data availability statement. This statement should provide the following information, where applicable:

- Accession codes, unique identifiers, or web links for publicly available datasets
- A description of any restrictions on data availability
- For clinical datasets or third party data, please ensure that the statement adheres to our policy

A data availability statement is included in the manuscript.
This is an Open Access article under the CC BY 4.0 license.
Clinical data was collected via PHOSP REDCap database, to which access is available under reasonable request as per the Data sharing statement in the manuscript.
Conventional immunoassay data were collected using Mesoscale Discovery workbench software or Fluostar Omega plate reader software and Microsoft Excel.
The PHOSP-COVID protocol, consent form, definition and derivation of clinical characteristics and outcomes, training materials, regulatory documents, information about requests for data access, and other relevant study materials are available online: https://phosp.org/resource/. Access to these materials can be granted by contacting phosp@leicester.ac.uk and Phospcontracts@leicester.ac.uk.
The ISARIC4C protocol, data sharing and publication policy are available at https://isaric4c.net. ISARIC4C's Independent Data and Material Access Committee welcomes applications for access to data and materials (https://isaric4c.net).
All data used in this study is available within ODAP and accessible under reasonable request. Data access criteria and information about how to request access is available online: https://phosp.org/resource/. If criteria are met and a request is made, access can be gained by signing the eDRIS user agreement.

The data sets used in the study contain extensive clinical information at individual level that prevent them from being deposited in an public depository due to data protection policies of the study. Study data can only be accessed via ODAP, a protected research environment. However as stated in the above statement, data access can be requested from the PHOSP COVID consortium.

Human research participants

Policy information about studies involving human research participants and Sex and Gender in Research.

Reporting on sex and gender	From the manuscript: 'Both sexes were recruited and gender was self-reported.' (methods)<br>The proportion of men and women in each group has been reported (Table 1)<br>Sex was included as a covariate in the analysis (Figure 1C-G). An exploratory analysis was performed to understand the female-bias of long COVID (Figure 3, Extended Data Figure 6 & Supplementary Table 2).
Population characteristics	Population characteristics are reported in Table 1 and have been previously reported in the following reference which is cited in the paper: Evans, R. A. et al. Physical, cognitive, and mental health impacts of COVID-19 after hospitalisation (PHOSP-COVID): a UK multicentre, prospective cohort study. Lancet Respir Med 9, 1275–1287 (2021).
Recruitment	From the manuscript:<br><br>'After hospital discharge patients >18 years old who had no co-morbidity resulting in a prognosis of less than 6 months, who had been hospitalised for COVID-19 were recruited to the PHOSP-COVID study. Patients that had been hospitalised between February 2020 and January 2021 were recruited. Both sexes were recruited and gender was self-reported. Written informed consent was obtained from all patients.'<br><br>'Clinical data and plasma samples were prospectively collected from adult cases of COVID-19 approximately 6 months after hospitalisation, via the PHOSP-COVID multicentre UK study.'<br><br>'Data relating to patient demographics and the acute admission were collected via the ISARIC4C study. Adults hospitalised during the SARS-CoV-2 pandemic were systematically recruited into the International Severe Acute Respiratory and Emerging Infection Consortium (ISARIC) World Health Organization Clinical Characterisation Protocol UK (IRAS260007 and IRAS126600). Written informed consent was obtained from all patients.'<br><br>Citations are provided in the text to the papers which fully detail recruitment and population characteristics: Methods, Reference number 15,14,16,96
Ethics oversight	From the manuscript:<br>'Ethical approvals for the PHOSP-COVID study were given by Leeds West Research Ethics Committee (20/YH/0225).'<br><br>'Adults hospitalised during the SARS-CoV-2 pandemic were systematically recruited into the International Severe Acute Respiratory and Emerging Infection Consortium (ISARIC) World Health Organization Clinical Characterisation Protocol UK (IRAS260007 and IRAS126600). Written informed consent was obtained from all patients. Ethical approval was given by the South Central–Oxford C Research Ethics Committee in England (reference: 13/SC/0149), Scotland A Research Ethics Committee (20/SS/0028) and World Health Organization Ethics Review Committee (RPC571 and RPC572l; 25 April 2013).'<br><br>'Written consent was obtained for all individuals and ethical approvals were given by London-Harrow Research Ethics Committee (13/LO/1899) for the collection of nasal samples and the Health Research Authority London–Fulham Research Ethics Committee (IRAS Project ID 154109; references 14/LO/1023, 10/H0711/94, and 11/LO/1826) for BALF samples.'

Note that full information on the approval of the study protocol must also be provided in the manuscript.

Field-specific reporting

Please select the one below that is the best fit for your research. If you are not sure, read the appropriate sections before making your selection.

☒ Life sciences ☐ Behavioural & social sciences ☐ Ecological, evolutionary & environmental sciences

For a reference copy of the document with all sections, see nature.com/documents/nr-reporting-summary-flat.pdf

Life sciences study design

All studies must disclose on these points even when the disclosure is negative.

Sample size

This was an exploratory observational study set up early in the pandemic to understand long term healthcomes after COVID-19. As such, UK patients were systematically recruited to understand sequelae and biological mechanisms at population level. As such, power calculations were not performed. Given the limited understanding of long COVID as a disease entity at the start of the study, power calculations and meaningful effect sizes are challenging to derive. However previous work published by the PHOSP-COVID consortium has indicated the this study size is sufficient to detect changes in inflammatory profiles 6 months after hospitalisation:

Evans, R. A. et al. Clinical characteristics with inflammation profiling of long COVID and association with 1-year recovery following hospitalisation in the UK: a prospective observational study. Lancet Respir Med 10, 761–775 (2022).

Furthermore, recent studies using Olink data in sample sizes substantially smaller than ours indicate our sample size is sufficient to detect meaningful proteomic differences between symptom groups:

Woodruff, M. C. et al. Chronic inflammation, neutrophil activity, and autoreactivity splits long COVID. Nat Commun 14, 4201 (2023).

Data exclusions

Individuals were excluded from the PLR analysis if covariate or symptom outcome or covariate data was missing. Olink data from analytes that did not pass QC measures were excluded.

Replication

To ensure the validity of results, samples were run in a single batch with use of negative controls, plate controls in triplicate, and repeated measurement of patient samples between plates in duplicate. Samples were randomized between plates according to site and sample collection date. Randomization between plates was blind to long COVID/ recovered outcome. Data were first normalized to an internal extension control that was included in each sample well. Plates were standardized by normalizing to inter-plate controls, run in triplicate on each plate. Each plate contained a minimum of 4 patient samples which were duplicates on another plate, these duplicate pairs allowed any plate to be linked to any other through the duplicates. Data were then intensity normalized across all cohort samples. Finally, Olink results underwent QC processing and samples or analytes that did not reach QC standards were excluded. Final normalized relative protein quantities were reported as log2 normalized protein expression (NPX) values. (Methods)

The QC and normalisation methods are according to best practice guidance which can be found here: https://www.olink.com/content/uploads/2022/04/white-paper-data-normalization-v2.1.pdf

A nested cross-validation was used to choose the optimal model for analysis and assess the stability of the model. The results of this have been reported in the manuscript (supplemental p3 and Extended Data Fig 9). Furthermore we validated our findings through repeated analysis restricted by Long COVID definition (Extended Data Fig 1) as well as through application of different models, unadjusted and non-regularised models and univariate analyses (Extended Data Fig 1-4, Supplementary Table 2).

Randomization

This was an observational, non-interventional study and symptom data was collected prospectively. To avoid bias introduced by assay error, non-specific binding or batch effects, samples were randomized between plates and run in a single batch blinded for long COVID/ recovered outcome. Randomization of samples across Olink assay plates is described in 'Replication'.

Blinding

Assays were run blinded to symptom outcome. Samples were randomized between plates with blinding for long COVID/ recovered outcome. Exclusion of data according to missingness or QC measures occured prior to primary analysis, without knowledge of clinical data, symptom outcome or individual analyte results.

Reporting for specific materials, systems and methods

We require information from authors about some types of materials, experimental systems and methods used in many studies. Here, indicate whether each material, system or method listed is relevant to your study. If you are not sure if a list item applies to your research, read the appropriate section before selecting a response.

Materials & experimental systems

n/a	Involved in the study
	☒ Antibodies
☒	Eukaryotic cell lines
☒	Palaeontology and archaeology
☒	Animals and other organisms
	☒ Clinical data
☒	Dual use research of concern

Methods

n/a	Involved in the study
☒	ChIP-seq
☒	Flow cytometry
☒	MRI-based neuroimaging

Antibodies

Antibodies used

Antibodies used were in the form of commercial ELISA, MSD and Luminex assays (details in online methods and supplementary page 1):

Biotechne: Human Luminex Discovery Assay, configuration Ubdal728; CAT NO: LXSAHM-15
MSD: V-PLEX Proinflammatory Panel1 (human) Kit (1 Plate); CAT NO: K15049D-1

MSD:
R-PLEX Human TFF2 Antibody Set ; CAT NO: F21ACM-3
R-PLEX Human TGF-α Antibody Set ; CAT NO: F21ACN-3
R-PLEX Human tPA Antibody Set ; CAT NO: F21ACZ-3
R-PLEX IL1R2 Antibody set; CAT No: K151ANVR-2
U-plex GCSF and IL2 custom multiplex kit; CAT No: K15067M-1

Abcam: IL3RA Elisa kit, CAT No: ab300317

Olink antibodies were selected in a 3 stage validation process:
1. Screening against an Olink designed antigen (Ag) pool developed over many years to detect unspecific binding.
2. After removal of poorly performing antibodies, a second screen was performed using a second Ag pool (n=92 Ags)
3. Validation of final product design was then performed against a pool of carefully selected proteins with documented high homology within their protein families (n=96 Ags).
Using these methods (details available: https://olink.com/content/uploads/2022/10/olink-explore-validation-data.pdf) Olink have demonstrated that 99.7% of Olink explore protein assays do not show any cross-reactivity or non-specific binding. The specific proteins that we found to be associated with Long COVID have all shown inter- and intra-CVs < 10, demonstrating very high precision. The full list of analytes in the Olink Explore inflammation panel and their associated precision can be found here: https://olink.com/content/uploads/2023/07/olink-explore-3072-validation-data-results.xlsx.

Validation

validation details are available from the MSD website as follows:

"MSD's validated assay kits meet the Clinical Laboratory Standards Institute guidelines for consistency, sensitivity, precision, and robustness. Validation testing is conducted through a design-control process according to the principles outlined in "Fit-for-Purpose Method Development and Validation for Successful Biomarker Measurement" by Lee, J.W. et al." Available from: https://www.mesoscale.com/en/support/product_information/search_coa/~/link.aspx?_id=C6380BC0C4514064875E4382146EC6B1&_z=z

Validation of Luminex cytokine assays has been published: dupont NC, Wang K, Wadhwa PD, Culhane JF, Nelson EL. Validation and comparison of luminex multiplex cytokine analysis kits with ELISA: determinations of a panel of nine cytokines in clinical sample culture supernatants. J Reprod Immunol. 2005 Aug;66(2):175-91. doi: 10.1016/j.jri.2005.03.005. PMID: 16029895; PMCID: PMC5738327.

The Olink Explore assays have been validated internally through a 3 stage process (see above). The full methods used are publicly available: https://olink.com/content/uploads/2022/10/olink-explore-validation-data.pdf
and
https://olink.com/content/uploads/2023/07/olink-explore-3072-validation-data-results.xlsx.

Using these methods, Olink have demonstrated that 99.7% of Olink explore protein assays do not show any cross-reactivity or non-specific binding. The specific proteins that we found to be associated with Long COVID have all shown inter- and intra-CVs < 10, demonstrating very high precision. The full list of analytes in the Olink Explore inflammation panel and their associated precision can be found here: https://olink.com/content/uploads/2023/07/olink-explore-3072-validation-data-results.xlsx.

Olink has also been validated against other assays and this data has been published (wik et al., 2021, https://doi.org/10.1016/j.mcpro.2021.100168; references 88-93 in manuscript, Discussion).

We further internally validated the assay using conventional MSD and ELISA kits (Extended Data Fig.8)

Clinical data

Policy information about clinical studies

All manuscripts should comply with the ICMJE guidelines for publication of clinical research and a completed CONSORT checklist must be included with all submissions.

Clinical trial registration

NA. This was not a clinical trial.

Study protocol

The PHOSP-COVID protocol, consent form, definition and derivation of clinical characteristics and outcomes, training materials, regulatory documents, information about requests for data access, and other relevant study materials are available online: https://phosp.org/resource/. Access to these materials can be granted by contacting phosp@leicester.ac.uk and Phospcontracts@leicester.ac.uk.

Data collection

Clinical data and plasma samples were prospectively collected from adult cases of COVID-19 approximately 6 months after hospitalisation, via the PHOSP-COVID multicentre UK study (see Methods).

The definition and derivation of clinical characteristics and outcomes can be accessed here: https://phosp.org/resource/. Access to these materials can be granted by contacting phosp@leicester.ac.uk and Phospcontracts@leicester.ac.uk.

Clinical data were collected through patient symptom questionnaires and validated clinical scores. The methods of clinical data collection have been extensively outlined and published and the references from the manuscript are included below:
14. Elneima, O. et al. Cohort Profile: Post-hospitalisation COVID-19 study (PHOSP-COVID). medRxiv 2023.05.08.23289442 (2023) doi:10.1101/2023.05.08.23289442.

15. Evans, R. A. et al. Clinical characteristics with inflammation profiling of long COVID and association with 1-year recovery following hospitalisation in the UK: a prospective observational study. Lancet Respir Med 10, 761–775 (2022).

Prospectively collected outcome and covariate data used are detailed in the manuscript 'Methods' section:
"Symptom data and samples were prospectively collected from individuals approximately 6 months after hospitalisation (Fig. 1A), via the PHOSP-COVID multicentre UK study... Clinical data were used to place patients into 6 categories: 'Recovered', 'GI', 'Cardiorespiratory', 'Fatigue', 'Cognitive impairment' and 'Anxiety/depression' (Supplementary Table 5). Patient reported symptoms and validated clinical scores were used including: MRC breathlessness score, dyspnoea-12 score, FACIT score, PHQ-9 and GAD-7. Responses to symptom questionnaires about chest pain and palpitations were also used. Cognitive impairment was defined as a Montreal Cognitive Assessment (MoCA) score <26. GI symptoms were defined as answering 'Yes' to the presence of at least two of the listed symptoms. 'Recovered' was defined by self-reporting. Patients were placed in multiple groups if they experienced a combination of symptoms."

Additional covariate data were also collected and analysed:

"Data were collected to account for variables affecting symptom outcome, via hospital records and self-reporting. Acute disease severity was classified according to the WHO Clinical Progression score: Class 3-4 required no oxygen, Class 5 required oxygen therapy, Class 6 required non-invasive ventilation or high-flow nasal oxygen), Class 7-9 were managed in critical care... Age, sex, acute disease severity and pre-existing comorbidities were included as covariates in the PLR analysis (Supplementary Table 1,2)"

This was a cross-sectional analysis of clinical data collected. No single individual provided repeated measures in the cohort that we analysed.

Outcomes

The primary outcome of this study was inflammatory profiles associated with symptoms occurring approximately 6 months after COVID-19 hospitalisation. We used nested controls within our cohort by using a group of individuals that reported feeling recovered. We compared inflammatory profiles in patients with symptoms and compared them to recovered controls within the cohort. The range of time-points at which this data was collected relative to acute infection is shown in Figure 1 A. This was a cross-sectional analysis of clinical data collected. No single individual provided repeated measures in the cohort that we analysed.

The full list of clinical outcomes measured by PHOSP-COVID and questionnaires used are publicly available (and this has been cited in the manuscript):
14. Elneima, O. et al. Cohort Profile: Post-hospitalisation COVID-19 study (PHOSP-COVID). medRxiv 2023.05.08.23289442 (2023) doi:10.1101/2023.05.08.23289442.

The specific variables used to define symptom outcomes in our study are detailed in Supplementary table 5, and described in Methods (see Excerpt above).

The primary outcome measure was analysed via the Olink Explore inflammatory panel using best practice methods (Methods). The association between inflammatory profiles and methods were measured using a penalised logistic regression model (Methods and Supplementary, page 2).
